# Supplementary material for: Cost-effectiveness of a high-intensity versus a low-intensity smoking cessation intervention in a dental setting: long-term follow-up
Source: BMJ Open. 2019 Aug 15;9(8):e030934. doi: 10.1136/bmjopen-2019-030934 (PMC6701567; doi:10.1136/bmjopen-2019-030934)
Supplement: Author's manuscript [file bmjopen-2019-030934.draft_revisions.pdf]

BMJ Open is committed to open peer review. As part of this commitment we make the peer review history of every article we publish publicly available.

When an article is published we post the peer reviewers' comments and the authors' responses online. We also post the versions of the paper that were used during peer review. These are the versions that the peer review comments apply to.

The versions of the paper that follow are the versions that were submitted during the peer review process. They are not the versions of record or the final published versions. They should not be cited or distributed as the published version of this manuscript.

BMJ Open is an open access journal and the full, final, typeset and author-corrected version of record of the manuscript is available on our site with no access controls, subscription charges or pay-per-view fees (http://bmjopen.bmj.com).

If you have any questions on BMJ Open's open peer review process please email info.bmjopen@bmj.com

BMJ Open

A high-intensity smoking cessation programme is sustainably cost-effective in comparison with a low-intensity programme in long-term. A cost-effectiveness analysis of a smoking cessation study

Journal:	*BMJ Open*
Manuscript ID	bmjopen-2019-030934
Article Type:	Research
Date Submitted by the Author:	09-Apr-2019

Complete List of Authors:	Feldman, Inna; Uppsala Universitet, Department of Public Health and Caring Science<br>Helgason, Asgeir; Karolinska Institutet, Department of Public Health Sciences, Social Medicine<br>Johansson, Pia; Public Health & Economics<br>Tegelberg, åke; Centre for Clinical Research, Uppsala University, Hospital of Vastmanland<br>Nohlert, Eva; Centre for Clinical Research, Uppsala University and Region Vastmanland,

Keywords:	smoking cessation, cost-effectivenes, long-term sustainability

SCHOLARONE™
Manuscripts

A high-intensity smoking cessation programme is sustainably cost-effective in comparison with a low-intensity programme in long-term. A cost-effectiveness analysis of a smoking cessation study

Inna Feldman[1], Ásgeir R Helgason[2.3], Pia Johansson[4], Åke Tegelberg[5.6] and Eva Nohlert[5]

[1] - Department of Public Health and Caring Sciences, Uppsala University, Uppsala, Sweden

[2] - Karolinska Institutet, Stockholm, Sweden

[3] - Reykjavik University & Icelandic Cancer Society, Reykjavik, Iceland

[4] - Public Health & Economics, Kyrkängsbacken 8, Huddinge, Sweden

[5] - Centre for Clinical Research, Uppsala University, Hospital of Västmanland, Västeras. Sweden

[6] - Faculty of Odontology, Malmö University, Malmö, Sweden

Corresponding author: Inna Feldman. inna.feldman@pubcare.uu.se

For peer review only

Abstract

Objectives. There is strong evidence on the effectiveness of tobacco control programmes. Previously, we performed a randomised controlled trial (RCT) of a high- and a low-intensity treatment programme (HIT and LIT) for smoking cessation in a dental setting in Sweden, where effectiveness was assessed after 1 and 5–8 years. The aim of this study is to conduct a cost-effectiveness analysis of HIT and LIT using long-term follow-up effectiveness data and to validate the previous cost-effectiveness results based on short-term follow-up.

Methods. The economic evaluation, performed from a societal perspective, was based on treatment costs and number of abstinent participants after 1 and 5–8 years. Future disease-related costs (in Euro (€) 2014) and health effects (in quality-adjusted life-years, QALYs) were estimated using a Markov model. Treatments were explicitly compared in an incremental analysis, and the results were presented as an incremental cost-effectiveness ratio (ICER).

Results. The more costly HIT led to higher number of 6-month continuous abstinent participants after 1 year and higher number of sustained abstinent participants after 5–8 years, which translates into larger societal costs avoided and health gains than LIT. The incremental cost/QALY of HIT compared to LIT amounted to €936 and €1,021 using short- and long-term effectiveness respectively, which is considered very cost-effective in Sweden.

Conclusion. The cost-effectiveness of the HIT treatment compared to LIT increased over time. Cost-effectiveness analysis favours the more costly HIT if decision-makers are willing to spend at least €1,000/QALY for tobacco cessation treatment.

For peer review only

Strengths and limitations of this study

- The study presents cost-effectiveness analysis of a high- and a low-intensity smoking cessation programme in a dental setting using long-term (5–8 years) follow-up data.
- The analyses show that high-intensity programme was sustainably cost-effective in comparison with low-intensity programme in the long-term.
- The study supports that more intensive and costly smoking cessation provision is cost-effective.
- The calculation of the intervention costs for the cessation programmes was based on a trial protocol and might be overestimated in comparison with routine praxis.
- The effects of smoking cessation are probably underestimated since only three disease groups are modelled and no effects of passive smoking are included.

Introduction

Smoking is likely to remain the single most important preventable health risk in the world. Despite continuously declining prevalence in recent decades, one in ten adults in Sweden still smoke daily [1]. Cigarette smoking contributes to 7.5% of the burden of disease in Sweden [2] and was estimated to account for approximately €3,000,000 (31.5 billion Swedish krona, SEK), including €1,000,000 (11 billion SEK) in healthcare costs (15% of the national costs for health and welfare sector) and €1,500,000 (16 billion SEK) in productivity costs in year 2015 [3]. A decrease in prevalence of smoking to five per cent could save society €1,300,000 (14.3 billion SEK) per year.

For peer review only

Several smoking cessation interventions, targeted at current smokers, are available; furthermore, evaluations so far have confirmed the effectiveness of the majority of them. Additionally, some recent studies emphasise that higher level of intervention intensity, such as additional counselling sessions [4] and intensive support through a mobile application [5], resulted in the highest smoking cessation rates. However, due to increasing number of available interventions, decision-makers have to decide which intervention to implement, taking into account that intervention intensity reflects intervention costs. Relative costs and benefits of those interventions are important criteria, thus, increasing the attention on economic evaluations in recent years [6 7]. Economic evaluations combine the costs and outcomes of different interventions and aim to determine which intervention provides the best value for money [8]. Several studies on the cost-effectiveness of smoking cessation interventions comparing different intensity of support have been performed during the last few years. For example, Quit-and-Win programme [9], comparison of standard, enhanced and intensive smoking cessation interventions using cell phones [10], and two smoking cessation approaches of different level of intensity for cancer patients [11]. The results suggested that the higher intensive interventions are preferable from health economics point of view, but all those evaluations were based on 6- or 12-months follow-up, long-term follow-ups are scarce in randomised controlled trials.

The effects of smoking on health occur during many years because current smoking influences future health risks; similarly, a smoking cessation today will cause smoking related health risks to tail off gradually. Thus, in order to estimate cost-effectiveness of smoking cessation interventions, a lifetime perspective is necessary, taking into account a variety of different costs and effects [12]. Hence, the well-established method to perform cost-effectiveness analyses of smoking cessation interventions involves mathematical modelling of future events as consequences of smoking. Systematic reviews of model-based economic

For peer review only - http://bmjopen.bmj.com/site/about/guidelines.xhtml

evaluations in smoking cessation analysed different aspects, such as type of model, quality of the model, transferability, and comparison of the results in different studies [12-14]. Berg at al. [13] identified 64 economic evaluations in smoking cessation, and the state-transition Markov model was most frequently used. The majority of the models simulates the lifetime development of morbidity and mortality for smoker vs former smoker using relative risks for four diseases, chronic obstructive pulmonary disease (COPD), coronary heart disease (CHD), stroke, and lung cancer. The authors concluded that existing economic evaluations in smoking cessation vary in quality, resulting mainly from the way in which they selected their populations, measured costs and effects, and assessed the variability and generalisability of their own findings [13]. One of the reasons is that all those studies are based on short-term follow-up (from six months to one year), and they never had a chance to validate the sustainability of short-term effectiveness in real life; thus they cannot confirm the reported cost-effectiveness results and policy recommendations. Moreover, the long-term assumption might change the results of the smoking cessation cost-effectiveness [15].

Our previous economic evaluation of high- and low-intensity programmes (HIT and LIT) for smoking cessation in a dental setting was based on the reported number of quitters measured as point prevalence abstinent (not one puff of smoke during the past seven days prior to 1-year follow-up). The conclusion was that high-intensity treatment support is the preferred option if the decision-maker's willingness-to-pay exceeds €5,100 (50,000 SEK) per QALY. The base-case scenario of the analysis assumed a sustained abstinence for the quitters [16]. The long-term follow-up of the programmes was performed five to eight years later [17]. In this study, we used a unique opportunity to compare cost-effectiveness analyses of a high- and a low-intensity smoking cessation intervention in a dental setting, using data from short-term (1-year) and relatively long-term (5–8 years) follow-up.

For peer review only

We set out to: 1) perform a cost-effectiveness analysis of a high- and a low-intensity smoking cessation programme in a dental setting using long-term (5–8 years) follow-up data and 2) compare the cost-effectiveness results with the previous study based on short-term (1-year) follow-up.

Methods

Summary of the smoking cessation study

In the smoking cessation intervention study [18], between August 2003 and February 2005, 300 adult smokers recruited via direct inquiry or advertising in dental or general health care were offered smoking cessation support performed in a dental setting. Inclusion criteria were daily smokers over 20 years of age, while exclusion criteria were reading difficulties and problems with Swedish language. The participants were randomly assigned to two interventions; one received high-intensity and one low-intensity treatment support.

The high-intensity smoking cessation treatment, the HIT programme, comprised eight individual sessions, of in total 3.5 hr over a period of 4 months, and was based on behaviour therapy, coaching and pharmacological advice. The low-intensity smoking cessation treatment, the LIT programme, comprised one counselling session, of up to 45 min, introducing a conventional self-help programme running over 8 weeks. Both programmes were free of charge.

The participants answered a baseline questionnaire and a short-term (one year after the planned smoking cessation date) follow-up questionnaire. The effectiveness of the trial was reported elsewhere [18]. The analysis concluded that the more extensive and expensive HIT programme was more effective, in terms of proportion of smokers who were still smoke-free after one year [18]. The long-term follow-up was performed 5–8 years after the planned

For peer review only

smoking cessation date. The effectiveness analysis showed that the difference in outcome between the HIT and LIT programmes remained relatively constant and significant in favour of HIT, and that abstinence at 1-year follow-up was a good predictor for long-term abstinence [17]. All analyses were done using the "intention to treat" approach where non-responders were considered as smokers. The original study, as well as the long-term follow-up, was approved by the ethical committee at Uppsala University (Dnr:Ups 02–457, Dnr: 2010/172).

The mean age of the participants was 49 years, and 78% were women. Short-term follow-up (one year) questionnaire was answered by 84% of the randomised participants (88% for HIT vs 81% for LIT). Fourteen per cent (41 of the 300 participants) reported 6-month continuous abstinence (not one puff of smoke during the past 6 month); 27 (18%) individuals in HIT vs 14 (9%) in LIT. At long-term follow-up (5–8 years), 241 persons answered the questionnaire (80% for both HIT and LIT). Of those, 24 were sustained abstinent (17 vs 7 for HIT vs LIT) since the planned smoking cessation date. Characteristics of the study participants as well as abstinence at the 1-year and at the long-term follow-up are presented in Table 1.

Table 1. Characteristics of the study participants and programmes effectiveness at the 1- and 5-8-year follow-up, by treatment intensity.

	HIT N=150	LIT N=150	p-value
**Study participants**			
Baseline measures	146	148	
12-month follow-up measures	132	122	
Available at long-term follow-up	141	143	
Long-term follow-up measures	121	120	
**Participants characteristics**			
Gender:			
Men	26	32	
Women	115	111	.410
Age at baseline:			
mean (SD)	48.7 (9.6)	48.5 (11.0)	.825

For peer review only

median	48.0	49.0	
Education in years:			
0 - 9	25	36	
10-12	60	55	
>=13	52	50	.336
Number of smoked cigarettes/week at baseline:			
mean (SD)	106 (50)	105 (40)	
median	105	105	.794
**Intervention effectiveness**			
1-year follow-up:			
6-month continuous abstinence	27	14	**.034***
5-8 year follow-up:			
Sustained abstinence	17	7	**.030***

* significant differences in effectiveness between the programmes

Economic evaluation

Two economic evaluations were performed to obtain the cost-effectiveness of the more costly HIT programme in comparison to LIT:

1) Cost-effectiveness analyses (CEA) based on the number of 6-month continuous abstinent participants according to 1-year follow-up, CEA short-term; and

2) Cost-effectiveness analyses based on sustained abstinent participants since planned smoking cessation date according to 5–8 years follow-up, CEA long-term.

Both analyses used the same methodology described below.

Economic evaluations were based on the costs to implement the programmes, the number of abstinent participants and on a previously constructed Markov model that estimates the future health and cost consequences of smoking cessation. The cost-effectiveness analyses followed Swedish and international recommendations: costs were calculated from a societal perspective, health effects expressed as quality-adjusted life-years (QALYs), and programmes explicitly compared in an incremental analysis (incremental cost-effectiveness ratio [ICER]),

For peer review only

with discounting (3% per year) and sensitivity analyses [8 19]. The ICER was calculated by dividing the difference in total costs for the programmes (incremental cost) by the difference in the health outcomes in QALYs (incremental effect) to provide a ratio of extra cost per extra unit of health effect.

Intervention costs

The intervention costs were collected prospectively by interviewing the three dental hygienists who carried out the patient work as well as the project leader and the project coordinator. The costs were divided into joint costs for the two programmes and programme-specific costs, and undiscounted because of the short 3-year project time. The joint costs were assumed, divided equally between the programmes while the programme-specific costs included staff time for patient work, material, and participant costs. Estimation of the intervention costs has been described in detail previously [16]. All costs were measured in Swedish kronor (SEK) in the year 2004. The costs were inflated to reflect 2014 costs according to the Swedish consumer price index [20] and converted into 2014 Euro (€) using the purchasing power parity (PPP) estimates with CCEMG – EPPI-Centre Cost Converter (http://eppi.ioe.ac.uk/costconversion/default.aspx). Total programme-specific costs amounted to €105,951 for HIT and €25,287 for LIT.

Intervention effectiveness

For CEA short-term, we used 6-month continuous abstinence at 1-year follow-up reported by 41 participants (14 from HIT and 27 from LIT). For CEA long-term, we used sustained abstinence at 5–8 years reported by 24 participants (17 from HIT and 7 from LIT), see Table 1. Both measures were significant different between the treatment programs.

For peer review only

Markov model

A Markov model was used to estimate health consequences and societal costs of smoking cessation, further described in a technical report [21]. The model has been used in similar studies in Sweden [16 22 23], and the updated year 2015 version was used for the current analysis [21]. The model simulates the societal effects of quitting smoking on three disease groups: lung cancer, chronic obstructive pulmonary disease (COPD) and cardiovascular disease, including coronary heart disease (CHD) and stroke. Even though there are other smoking-related diseases, these conditions cover most of the health problems associated with smoking [24].The model incorporates the smoking-related disease risks, time-dependent remaining excess disease risks after quitting, the death risks for the specific and for unrelated diseases, as well as the societal costs of the diseases. All disease risks are annual age- and gender-specific excess incidence risks until death or the age of 95. The societal costs include costs associated with: medical treatment, community care, drugs, informal care and other expenditures for patients and relatives as well as morbidity productivity costs. Health outcomes are expressed in QALYs. The number of QALYs were calculated during healthy years and years spent with a disease, until death or the age of 95. The model and all the parameters are described in detail in a technical report [21] and Appendix 1.

Model simulation were performed according to gender and age groups. The simulations result in accumulated societal costs and health effects for life-long continuing smokers and quitters at a specific age and gender group, respectively. The differences in societal costs and health effects between smoking statuses at a certain age are then compared outside the model, and constitute the avoided costs and gained health effects from the tobacco quitting for the specified age and gender group.

For peer review only

Sensitivity analyses

Extensive sensitivity analyses on parameter values and methodological choices were reported in the model technical report [21]. The model estimates were, in general, insensitive to changes in parameter values, except the most conservative multivariate analysis where the costs were decreased by 25%, the disease risks by 50%, the death risks by 10%, and the risk fractions after quitting by 0.1. This low cost/low risk analysis led to substantial decreases in cost and QALY differences between quitters and smokers. This sensitivity analysis was applied to compare costs and effects between HIT and LIT, to validate the results of the CEA-long term. A probabilistic sensitivity analysis (PSA) was also conducted, based on the uncertainty of the difference in sustained abstinent participants in the two programmes. The effectiveness of LIT was fixed at the 7% quit rate, but the HIT quit rate was sampled from the 95% confidence interval (9% – 22%). The PSA was performed by 1000 runs, using the societal costs avoided and QALY gains for the group with the largest number of quitters, i.e. women aged 40–44 years. The PSA was presented as a cost-effectiveness acceptability curve, which indicates the probability that HIT is cost-effective versus LIT at different values of the willingness-to-pay for a QALY.

No Patient and Public Involvement

This research was done without patient involvement. Patients were not invited to comment on the study design and were not consulted to develop patient relevant outcomes or interpret the results. Patients were not invited to contribute to the writing or editing of this document for readability or accuracy.

For peer review only

12

Results

Model estimations

Model estimations for the CEA short-term and CEA long-term are presented in Table 2 (societal costs and QALYs). The second column in Table 2 under subtitle "Model estimation costs avoided" presents the estimation of avoided societal costs for a person with respective gender and age, who became sustained abstinent in comparison with a continuing smoker. For example, women in age group 25–29 years who quit smoking will cost society €9.345 less compared with women in the same age who continue to smoke. Using this data, we can estimate the societal cost avoided for respective treatment programme by multiplying number of 6-month continuous abstinent participants (n*) or number of sustained abstinent participants since planned smoking cessation date (n**) by societal costs avoided. Further, the second column in Table 2 under subtitle "Model estimation QALYs gained" presents the estimation of additional QALYs for a person with respective gender and age, who became sustained abstinent in comparison with a continuing smoker. For example, women in age group 25–29 years who quit smoking will get additional 0.65 QALYs until age 95 compared with women in the same age who continue to smoke. Using this data, we can estimate the QALYs gained for respective treatment by multiplying number of point prevalence abstinent participants (n*) or number of sustained abstinent participants since planned smoking cessation date (n**) by societal costs avoided.

For peer review only

Table 2. Model estimation of societal costs avoided and QALYs gained. Costs in Euro 2014. 3% discount rate.

Age group	Model estimation costs avoided	Social costs avoided: CEA[a]-short: HIT[b]		LIT[c]		Difference		CEA[a]-long: HIT[c]		LIT[b]		Difference	
		n*	Costs	n*	Costs	n*	Costs	n**	Costs	n**	Costs	n**	Costs
Women													
20-24	9,032	1	9,032		0	1	9,032		0		0	0	0
25-29	9,345		0	1	9,345	-1	-9,345		0		0	0	0
35-39	10,279	2	20,559	2	20,559	0	0	1	10,279	1	10,279	0	0
40-44	9,464	5	47,318		0	5	47,318	4	37,855		0	4	37,855
45-49	7,512	3	22,536	3	22,536	0	0	1	7,512	2	15,024	-1	-7,512
50-54	5,799	4	23,195	3	17,396	1	5,799	1	5,799	2	11,597	-1	-5,799
55-59	5,039	4	20,155	2	10,077	2	10,077	4	20,155	1	5,039	3	15,116
60-64	3,700	4	14,800		0	4	14,800	2	7,400		0	2	7,400
65-69	2,244		0	1	2,244	-1	-2,244		0		0	0	0
Men													
20-24	11,570	1	11,570	1	11,570	0	0	1	11,570		0	1	11,570
25-29	11,823		0		0	0	0		0		0	0	0
40-44	11,676	1	11,676		0	1	11,676	1	11,676		0	1	11,676
45-49	11,091	1	11,091	1	11,091	0	0	1	11,091	1	11,091	0	0
50-54	8,913		0	1	8,913	-1	-8,913		0		0	0	0
55-59	5,788		0		0	0	0		0		0	0	0
60-64	4,531	1	4,531		0	1	4,531	1	4,531		0	1	4,531
**Total**		**27**	**187,429**	**14**	**113,731**	**13**	**73,698**	**17**	**127,866**	**7**	**53,030**	**10**	**74,836**

For peer review only

Age group	Model estimation QALYs gained	QALYs [d] gained: CEA[a]-short HIT[b]		CEA[a]-short LIT[c]		CEA[a]-short Difference		CEA[a]-long HIT[b]		CEA[a]-long LIT[c]		CEA[a]-long Difference	
		n*	QALYs	n*	QALYs	n*	QALYs	n**	QALYs	n**	QALYs	n**	QALYs
Women													
20-24	0.61	1	0.00		0,61	1	-0.61						
25-29	0.65		0,65	1	0,00	-1	0.65		0.00		0.00	0	0.00
35-39	0.71	2	1.41	2	1,41	0	0.00	1	0.71	1	0.71	0	0.00
40-44	0.71	5	3.55		0,00	5	3.55	4	2.84		0.00	4	2.84
45-49	0.66	3	1.98	3	1,98	0	0.00	1	0.66	2	1.32	-1	-0.66
50-54	0.61	4	2.44	3	1,83	1	0.61	1	0.61	2	1.22	-1	-0.61
55-59	0.43	4	1.72	2	0,86	2	0.86	4	1.72	1	0.43	3	1.29
60-64	0.32	4	1.29		0,00	4	1.29	2	0.64		0.00	2	0.64
65-69	0.33		0.00	1	0,33	-1	-0.33		0.00		0.00	0	0.00
Men													
20-24	0.74	1	0.74		0,00	1	0.74	1	0.74		0.00	1	0.74
25-29	0.81		0.00		0,00	0	0.00		0.00		0.00	0	0.00
40-44	1.00	1	1.00		0,00	1	1.00	1	1.00		0.00	1	1.00
45-49	0.82	1	0.82	1	0,82	0	0.00	1	0.82	1	0.82	0	0.00
50-54	0.78		0.00	1	0,78	-1	-0.78		0.00		0.00	0	0.00
55-59	0.60		0.00		0,00	0	0.00		0.00		0.00	0	0.00
60-64	0.46	1	0.46		0,00	1	0.46	1	0.46		0.00	1	0.46
**Total**		**27**	**16.05**	**14**	**8.61**	**13**	**7.44**	**17**	**10.20**	**7**	**4.49**	**10**	**5.71**

For peer review only

n* - number of 6-month continuous abstinent participants according to 1-year follow-up
n** - number of sustained abstinent participants according to 5-8 year follow-up
[a] – Cost-effectiveness analysis
[b] – High-intensity smoking cessation treatment, the HIT programme
[c] – Low- intensity smoking cessation treatment, the LIT programme
[d] – Quality adjusted life years

The CEA short-term indicated that HIT led to additional avoided societal costs of €73.698 and additional 7.44 QALYs, compared with LIT. The CEA long-term reported the difference between HIT and LIT as additional avoided societal costs of €74.836 and additional 5.71 QALYs.

Cost-effectiveness analyses

The more costly HIT programme led to a higher number of 6-month continuous abstinent participants at 1-year follow-up (CEA short-term) as well as higher number of sustained abstinent participants at 5–8 year follow-up (CEA long-term), which translates into larger costs avoided and health gains than LIT, see Table 3. However, the difference in intervention costs were not fully balanced by the societal costs avoided, so HIT implied an incremental net cost of about €6,966 in CEA short-term and €5,828 in CEA long-term, compared with LIT. HIT was estimated to lead to more QALYs, so the incremental cost per QALY of HIT compared with LIT amounted to €936 for CEA short-term and €1,021 for CEA long-term, which is considered to be very cost-effective in Sweden [19]. The incremental analysis favours the more costly HIT, if decision-makers are willing to spend at least €1,000/QALY for tobacco cessation programmes.

For peer review only

Table 3. Incremental cost-effectiveness analyses, CEA, of the two smoking cessation treatments, HIT and LIT, for 6-month continuous abstinence at 1-year (CEA short-term), sustained abstinence at 5–8 year follow-up (CEA long-term), and sensitivity analysis for CEA long-term. Societal perspective, in Euro 2014.

**Intervention costs**	**CEA-short**		**CEA-long**		**CEA-long, sensitivity**	
HIT	105,951		105,951		105,951	
LIT	25,287		25,287		25,287	
Difference in intervention costs		*80,664*		*80,664*		*80,664*
Societal costs avoided						
HIT	187,429		127,866		66, 312	
LIT	113,731		53,030		30, 297	
Difference in societal costs avoided		*73,698*		*74,836*		*36,016*
**Incremental costs**		**6,966**		**5,828**		**44,648**
QALYs						
HIT	16.05		10.2		8.12	
LIT	8.61		4.49		3.3	
**Incremental QALYs**		**7.44**		**5.71**		**4.82**
**Incremental cost per QALY (ICER)**		**936**		**1,021**		**9,263**

* Incremental cost-effectiveness ratio (ICER) is calculated as incremental costs divided by incremental QALYs

[a] – Cost-effectiveness analysis
[b] – High-intensity smoking cessation treatment, the HIT programme
[c] - Low- intensity smoking cessation treatment, the LIT programme
[d] – Quality adjusted life years

Sensitivity analyses

The most conservative sensitivity analysis, a multivariate low cost/low risk analysis, was applied to CEA long-term. This analysis led to substantial decreases in avoided social costs and QALY gains for both HIT and LIT. At the same time, the incremental costs increased and

For peer review only

incremental QALYs slightly decreased which resulted in higher incremental cost of €9,263 per QALY, see Table 3.

At all values of willingness-to-pay for a QALY, including zero, the HIT was more cost-effective than the LIT, see the probabilistic sensitivity analysis in Figure 1.

(insert figure 1 here)

Figure 1. Probabilistic sensitivity analysis on the effectiveness (proportion of quitters) of high-intensity treatment (HIT) in comparison with low-intensity treatment (LIT), reported as cost-effectiveness acceptability curve, willingness-to-pay per quality-adjusted life-year (QALY), in Euro 2014.

Discussion

Main results

In this study, we performed a cost-effectiveness analysis using the long-term follow-up data from a RCT of a high- and a low-intensity treatment programme (HIT and LIT) for smoking cessation in a dental setting. We also validated the cost-effectiveness results of the previous study based on short-term follow-up. HIT was more effective in getting participants to quit smoking and to keep sustained abstinent, resulted in higher societal costs avoided and more QALYs gained among both men and women, compared with LIT and thus was more cost-effective. The incremental cost-effectiveness ratios (ICERs) were €936 and €1,020 using short- and long-term effectiveness, respectively, which are below the Swedish willingness-to-pay threshold of €50,000 per QALY [25], thus, indicating that the resource intensive HIT was highly cost-effective. The results also confirm the conclusions of the previous cost-effectiveness analyses based on short-term follow-up data, and we would recommend the use of the HIT programme as a cost-effective option for smoking cessation.

For peer review only

Notably, the usage of both the HIT and LIT programmes is not limited to dental settings and can be implemented in other healthcare sectors and delivered by trained nurses instead of dental hygienists. Since the salaries of registered nurses and dental hygienists are comparable, the conclusion of high cost-effectiveness of the HIT programme remains.

However, although HIT was shown to be cost-effective in comparison with LIT, the incremental QALYs gained for men receiving HIT over men receiving LIT were considerably lower than for women. The HIT programme was most beneficial to women.

Strength and limitations

The majority of cost-effectiveness analyses on smoking cessation use one year quit rates in their models; however, it is not uncommon that 6-month quit rates are used [12 26]. The question of how much we are able to trust the overall conclusions of such analyses always remains, because we do not know for sure what happens subsequently. To our knowledge, this is the first study that utilises a unique possibility to compare a previously conducted cost-effectiveness analyses based on 6-month continuous abstinent participants at 1-year follow-up with a new evaluation, based on sustained abstinence since the planned smoking cessation date up to 5–8 years. We were able to compare the results based on 6-month continuous abstinence (when some time-dependent excess disease risks remained for the first years after quitting) and sustained abstinence for 5–8 years (when the smoking-related excess disease risks had been reduced). A higher proportion of sustained abstinent participants in HIT compared to LIT contributed to a lower ICER for the long-term cost-effectiveness analyses. The effects of smoking cessation are probably underestimated since only three disease groups are modelled and no effects of passive smoking are included. Avoided costs (and thus decreased net costs) as well as QALYs gained could be higher; consequently, the costs per

For peer review only

QALY decreased for both programmes. As mentioned in our previous study [16], the Markov model indicates considerably lower smoking-related disease risks for women reported by large epidemiological studies (see model technical report for details) [21], and thus lower cost savings and health gains from tobacco cessation for women than for men. Finally, the intervention costs for the RCT study calculation was based on the trial protocol and might be overestimated in comparison with routine praxis; however, in the ICER, those extra costs were divided equally between the programmes, and thus disregarded.

Comparison with other studies

We could not find any cost-effectiveness analyses based on more than 1-year follow-up, and therefore we compared our results with other studies estimating cost-effectiveness of interventions with different level of intensity using 6- or 12-month follow-up. Thus, a cost-effectiveness analysis of high intensity multiple contests and low intensity enhanced contest of a Quit-and-Win programme reported that high intensity Quit-and-Win leads to an average gain of 0.03 QALYs and was cost-saving, in comparison with lower intensity [9]. Another study presented a cost-effectiveness analysis of three smoking cessation interventions with different intensity levels: Standard Care (SC) (brief advice to quit, nicotine replacement therapy and self-help written materials), Enhanced Care (EC) (SC plus cell phone-delivered messaging) and Intensive Care (IC) (EC plus cell phone-delivered counselling) [10]. The overall conclusion was that the higher intensive intervention (IC) was the most cost-effective strategy both for men and women, which is in line with our results. Additionally, a cost-effectiveness analysis of two smoking cessation approaches for cancer patients was presented in a study from Canada [11]. The basic programme consisted of screening for tobacco use, advice and referral, whereas the best practice programme included a basic programme and pharmacological therapy, counselling and follow-up. The incremental cost-effectiveness ratio of the best

For peer review only

practice programme compared to the basic programme was $3,367 per QALY gained for men, and $2,050 per QALY gained for women. These results are very similar to our findings. In our previous study [16], based on the same RCT and 1-year follow-up, a higher ICER of €9,900/QALY and €5,500 /QALY was calculated for point prevalence and continuous abstinence respectively, but the overall conclusion confirmed the cost-effectiveness of HIT at a willingness-to-pay of €10,000.

Conclusions

In conclusion, the more costly HIT smoking cessation programme has the potential to be an economically attractive option when compared to the LIT programme over a broad range of assumptions. The HIT programme was sustainable cost-effective in comparison with the LIT programme in the long-term. Cost-effectiveness analysis favours the more costly HIT if decision-makers are willing to spend at least €1,000/QALY for tobacco cessation treatment. These findings can support and guide implementation of smoking cessation programmes.

Contributors

IF and EN conceived and designed the study and drafted the manuscript. Economic evaluation was carried out by IF and PJ. AR, ÅT and EN were responsible for clinical evaluation of the smoking cessation study. All the authors (IF, AR, ÅT, PJ and EN) contributed to the writing process and have approved the final manuscript.

Funding

This study was funded by grants from the County Council of Västmanland, Sweden (LTV 3999) and Swedish Research Council for Health, Working Life and Welfare (FORTE), grant number 2014-1399.

For peer review only

Competing interests

None declared.

Ethics approval

The Ethical Committee Uppsala University gave clearance for the smoking cessation study Dnr: Ups 02-457.

Data sharing statement

Data is available from corresponding author (IF) on reasonable request.

References

1. [Folkhälsomyndigheten] PHAoS. Public Health Agency of Sweden [Folkhälsomyndigheten] Stockholm2018 [Available from: https://www.folkhalsomyndigheten.se/the-public-health-agency-of-sweden/living-conditions-and-lifestyle/alcohol-narcotics-doping-tobacco-and-gambling/tobacco/ accessed 2018-05-05 2018.
2. Institute for Health Metrics and Evaluation. Country Profiles: Sweden [Available from: http://www.healthdata.org/sweden accessed Nov 5 2015.
3. Andersson E, Toresson Grip E, Norrlid H, et al. Samhällskostnaden för rökningsrelaterad sjuklighet i Sverige [The social cost of smoking-related illness in Sweden]. In: IHE, ed. IHE Rapport 2017:4. Lund, Sweden, 2017.
4. Thomas JL, Luo X, Bengtson J, et al. Enhancing Quit & Win contests to improve cessation among college smokers: a randomized clinical trial. *Addiction* 2016;111(2):331-9. doi: 10.1111/add.13144 [published Online First: 2016/01/16]
5. Vidrine DJ, Fletcher FE, Danysh HE, et al. A randomized controlled trial to assess the efficacy of an interactive mobile messaging intervention for underserved smokers: Project ACTION. *BMC public health* 2012;12:696. doi: 10.1186/1471-2458-12-696 [published Online First: 2012/08/28]
6. McFarland A. Economic evaluation of interventions in health care. *Nursing standard* 2014;29(10):49-58. doi: 10.7748/ns.29.10.49.e9315
7. Anderson R. Systematic reviews of economic evaluations: utility or futility? *Health economics* 2010;19(3):350-64. doi: 10.1002/hec.1486
8. Drummond M, Sculpher M, Torrance G, et al. Methods for the economic evaluation of health care programmes. 3rd edn ed. Oxford: Oxford University Press 2005.
9. Popp J, Nyman JA, Luo X, et al. Cost-effectiveness of enhancing a Quit-and-Win smoking cessation program for college students. *The European journal of health economics : HEPAC : health*

For peer review only

economics in prevention and care 2018 doi: 10.1007/s10198-018-0977-z [published Online First: 2018/04/25]
10. Daly AT, Deshmukh AA, Vidrine DJ, et al. Cost-effectiveness analysis of smoking cessation interventions using cell phones in a low-income population. *Tobacco control* 2018 doi: 10.1136/tobaccocontrol-2017-054229 [published Online First: 2018/06/11]
11. Djalalov S, Masucci L, Isaranuwatchai W, et al. Economic evaluation of smoking cessation in Ontario's regional cancer programs. *Cancer medicine* 2018 doi: 10.1002/cam4.1495 [published Online First: 2018/07/19]
12. Bolin K. Economic evaluation of smoking-cessation therapies: a critical and systematic review of simulation models. *PharmacoEconomics* 2012;30(7):551-64. doi: 10.2165/11590120-000000000-00000
13. Berg ML, Cheung KL, Hiligsmann M, et al. Model-based economic evaluations in smoking cessation and their transferability to new contexts: a systematic review. *Addiction* 2017;112(6):946-67. doi: 10.1111/add.13748
14. Kirsch F. A systematic review of quality and cost-effectiveness derived from Markov models evaluating smoking cessation interventions in patients with chronic obstructive pulmonary disease. *Expert review of pharmacoeconomics & outcomes research* 2015;15(2):301-16. doi: 10.1586/14737167.2015.1001976 [published Online First: 2015/01/21]
15. Pennington B, Filby A, Owen L, et al. Smoking Cessation: A Comparison of Two Model Structures. *PharmacoEconomics* 2018 doi: 10.1007/s40273-018-0657-y [published Online First: 2018/05/08]
16. Nohlert E, Helgason AR, Tillgren P, et al. Comparison of the cost-effectiveness of a high- and a low-intensity smoking cessation intervention in Sweden: a randomized trial. *Nicotine & tobacco research : official journal of the Society for Research on Nicotine and Tobacco* 2013;15(9):1519-27. doi: 10.1093/ntr/ntt009
17. Nohlert E, Ohrvik J, Tegelberg A, et al. Long-term follow-up of a high- and a low-intensity smoking cessation intervention in a dental setting--a randomized trial. *BMC public health* 2013;13:592. doi: 10.1186/1471-2458-13-592 [published Online First: 2013/06/20]
18. Nohlert E, Tegelberg A, Tillgren P, et al. Comparison of a high and a low intensity smoking cessation intervention in a dentistry setting in Sweden: a randomized trial. *BMC public health* 2009;9:121. doi: 10.1186/1471-2458-9-121 [published Online First: 2009/05/02]
19. Pharmaceutical Benefits Board. General guidelines for economic evaluations from the Pharmaceutical Benefits Board, 2003.
20. Statistics Sweden. Konsumentprisindex [Available from: http://www.scb.se/pr0101/ accessed April 14, 2016.
21. Johansson P. A model for economic evaluations of smoking cessation interventions – technical report. Model version 3. Folkhälsa & Ekonomi (Public Health & Economics), 2015.
22. Johansson PM, Tillgren PE, Guldbrandsson KA, et al. A model for cost-effectiveness analyses of smoking cessation interventions applied to a Quit-and-Win contest for mothers of small children. *Scandinavian journal of public health* 2005;33(5):343-52.
23. Virtanen SE, Galanti MR, Johansson PM, et al. Economic evaluation of a brief counselling for smoking cessation in dentistry: a case study comparing two health economic models. *BMJ open* 2017;7(7):e016375. doi: 10.1136/bmjopen-2017-016375 [published Online First: 2017/07/22]
24. Gunning-Schepers LJ. The health benefits of prevention: a simulation approach. *Health policy and planning* 1989(12):1-256.
25. Svensson M, Nilsson FO, Arnberg K. Reimbursement Decisions for Pharmaceuticals in Sweden: The Impact of Disease Severity and Cost Effectiveness. *PharmacoEconomics* 2015;33(11):1229-36. doi: 10.1007/s40273-015-0307-6 [published Online First: 2015/06/22]

26. Chen YF, Madan J, Welton N, et al. Effectiveness and cost-effectiveness of computer and other electronic aids for smoking cessation: a systematic review and network meta-analysis. *Health technology assessment* 2012;16(38):1-205, iii-v. doi: 10.3310/hta16380 [published Online First: 2012/10/11]

For peer review only

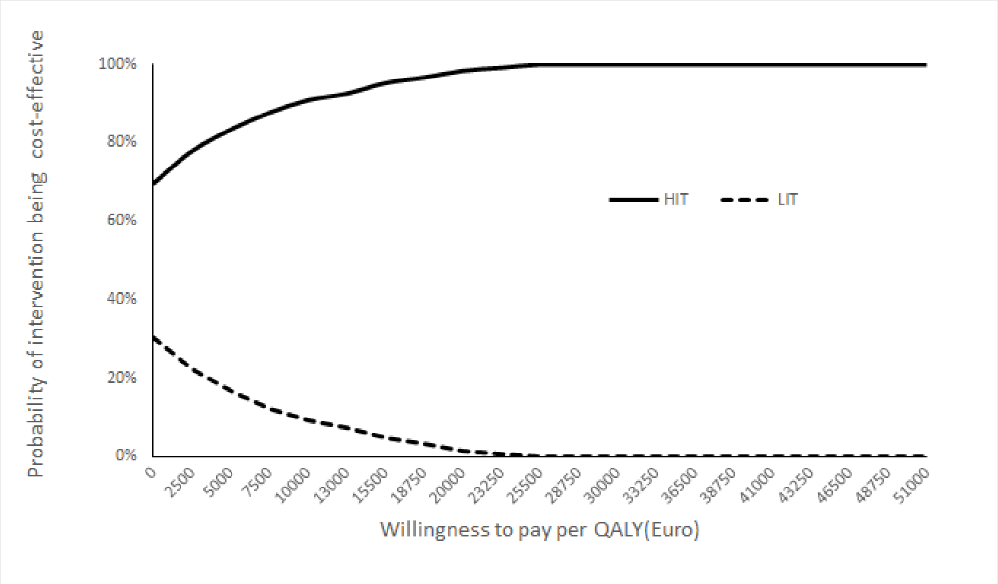

Figure 1. Probabilistic sensitivity analysis on the effectiveness (proportion of quitters) of high-intensity treatment (HIT) in comparison with low-intensity treatment (LIT), reported as cost-effectiveness acceptability curve, willingness-to-pay per quality-adjusted life-year (QALY), in Euro 2014.

A model for economic evaluations of smoking cessation interventions – technical report

Version 3 year 2015

Pia Johansson

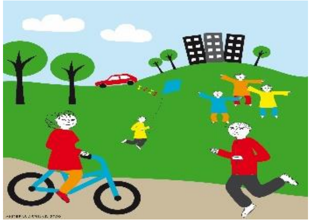

Folkhälsa&Ekonomi
PublicHealth&Economics

Stockholm 2015

Pia.johansson@folkhälsaetekonomi.se
Pia.johansson@publichealtheteconomics.se

This report is available at PublicHealth&Economics.se:
http://www.publichealtheteconomics.se

For peer review only

Content

TABLES **3**

INTRODUCTION **4**

METHOD **5**

THE DISEASES 5
THE MODEL 5

MATERIAL **7**

THE RISKS 7
Disease risks *7*
Death risks *10*
Changes in risk after quitting smoking *12*
THE SOCIETAL COSTS 12
Medical treatment costs *13*
Institutional care and technical aids costs *13*
Pharmaceutical costs *14*
Informal care and other patient and relatives' costs *14*
Productivity costs *15*
THE HEALTH EFFECTS 15
Life years lost *15*
QALYs *15*
SENSITIVITY ANALYSES 16
Univariate analyses *17*
Multivariate analyses *17*
Analyses on methodological issues *17*
Probabilistic analysis *17*

RESULTS **18**

THE MODEL ESTIMATES 18
SELECTED MODEL OUTCOMES 20
SENSITIVITY ANALYSES 22

DISCUSSION: MODEL VALIDITY **25**

THE STRUCTURE OF THE MODEL 25
THE INPUTS OF THE MODEL 26
THE RESULTS OF THE MODEL 28
THE VALUE OF THE MODEL TO THE DECISION-MAKER 29
THE UNCERTAINTY 30
CHECKING FOR TECHNICAL ERRORS 30
CONCLUSIONS 31

REFERENCES **32**

For peer review only

For peer review only - http://bmjopen.bmj.com/site/about/guidelines.xhtml

Tables

Table 1. The model diseases, with ICD-10 codes. 5
Figure 1. State-transition diagram 6
Table 2. Risks COPD. 7
Table 3. Risks lung cancer. 8
Table 4. Risks CHD and stroke. 8
Table 5. The annual risks of CHD. 9
Table 6. The annual risks of stroke. 9
Table 7. Distribution of diseases within CHD. 9
Table 8. Death risk lung cancer. 10
Table 9. Death risk AMI, 1st year. 10
Table 10. Death risk stroke, 1st year. 11
Table 11. Death risk CHF. 11
Table 12. Death risks, unrelated. 11
Table 13. Medical treatment costs. SEK 2014. 12
Table 14. Costs for institutional care and technical aids. SEK 2014. 13
Table 15. Pharmaceutical costs. SEK 2014. 14
Table 16. Informal care and other patient and relatives' costs. SEK 2014. 14
Table 17. Productivity costs, morbidity. SEK 2014. 15
Table 18. Average Swedish population QoL weights. 16
Table 19. QoL weights and QoL decrements due to disease. 16
Table 20. QALYs, until age 95 years, discounted 3%. 18
Table 21. Life years lost (YLS), before age 95 years. Discounted 3%. 19
Table 22. Life years lost (YLS), before age 95 years. Undiscounted. 19
Table 23. Societal costs. In SEK 2014 and discounted 3%. 20
Figure 2. The disease outcome, number of diseased and dead per 10 000 for smokers and quitters, women aged 50 years. 20
Figure 3. The disease outcome, number of diseased and dead per 10 000 for smokers and quitters, men aged 50 years. 21
Table 24. Societal cost savings, in SEK 2014. Women aged 50 years. 21
Table 25. Societal cost savings, in SEK 2014. Men aged 50 years. 22
Figure 4. Sensitivity analyses on societal cost and QALY differences between smokers and quitters, women aged 50 years. 22
Figure 5. Sensitivity analyses on societal cost and QALY differences between smokers and quitters, men aged 50 years. 23
Figure 6. The cost-effectiveness plane with resultat från bootstrap, women aged 50 years. 23
Figure 7. The cost-effectiveness plane with resultat from bootstrap, men aged 50 years. 24

For peer review only

For peer review only - http://bmjopen.bmj.com/site/about/guidelines.xhtml

Introduction

This is a technical report on an updated version of a model, originally developed in year 2004 (Johansson, 2004), to enable systematic cost-effectiveness analyses of tobacco cessation interventions in Sweden. It aims to follow international and Swedish recommendations of cost-effectiveness analyses in health and medicine. The model holds a societal perspective, aiming to incorporate available disease-specific costs for all sectors of Swedish society. The updated model contains more recent data on societal costs, disease and death risks, and quality-of life-estimates, to enable estimates that reflects current Swedish conditions.

The model simulates the lifetime societal effects of quitting smoking on three diseases: lung cancer, chronic obstructive pulmonary disease (COPD), and cardiovascular disease (CVD) including coronary heart disease (CHD) and stroke. The model incorporates the smoking-related disease risks, the remaining disease risks after tobacco quitting, the death risks in the diseases and unrelated diseases, as well as the societal effects of the diseases. The societal effects include medical treatment costs, costs for institutional care, drug costs, costs for informal care and other costs for patients and relatives, and morbidity productivity costs, as well as loss of life-years and quality-adjusted life-years (QALYs).

This technical report contains a description of the model structure, of all the data sources used and of the assumptions made. For validation purposes, it also reports model estimates for some selected age-groups and more detailed outcomes and sensitivity analyses for one age-group, men and women aged 50 years at the start of the simulations. To investigate model uncertainty, univariate and multivariate sensitivity analyses are reported, as well as a probabilistic analysis. The model validity is discussed in the final section of the report.

For peer review only

For peer review only - http://bmjopen.bmj.com/site/about/guidelines.xhtml

Method

The diseases

The model incorporates the three most smoking-related diseases: lung cancer, chronic obstructive pulmonary disease (COPD), and cardiovascular disease (CVD) including coronary heart disease (CHD) and stroke, see table 1. The model is restricted to the effects on the individual smoker/quitter, thus not incorporating any side-effects on other people.

The model

The stochastic model simulates the societal effects of smoking cessation on three smoking-related diseases. It is constructed as a Markov-cycle tree model appropriate for microsimulations.

The Markov model is a health state-transition model (Sonnenberg & Beck, 1993; Briggs & Sculpher, 1998) using probabilities for transitions between health states. These probabilities are the age- and gender-specific disease risks, conditional on smoking status and years since quitting, and age-, gender- and disease-specific death risks. The states are mutually exclusive and collectively exhaustive, and transitions between disease states are not allowed. The only exits from disease states are death, in the disease in question or in unrelated diseases, except for 5-year survivors in lung cancer which are assumed to recover to complete health. All other disease states are assumed to last life-long. See figure 1 for the state-transition diagram.

The Markov stages are one year-long, with no half-cycle correction. The starting point is the state healthy. The model covers the ages 15 to 95 years. The Markov-cycle tree has been created in Treeage Pro (Treeage Inc., 2015).

For peer review only

Table 1. The model diseases, with ICD-10 codes.

Disease	ICD-10
Lung cancer	C34
COPD	J44
Stroke	I61 I63 I64
Coronary heart disease, CHD:	
Acute myocardial infarction, AMI	I21 I22 I23
Congestive health failure, CHF	I50.
Ischemic heart disease, IHD	I20 I24 I25
Sudden death	I46.1

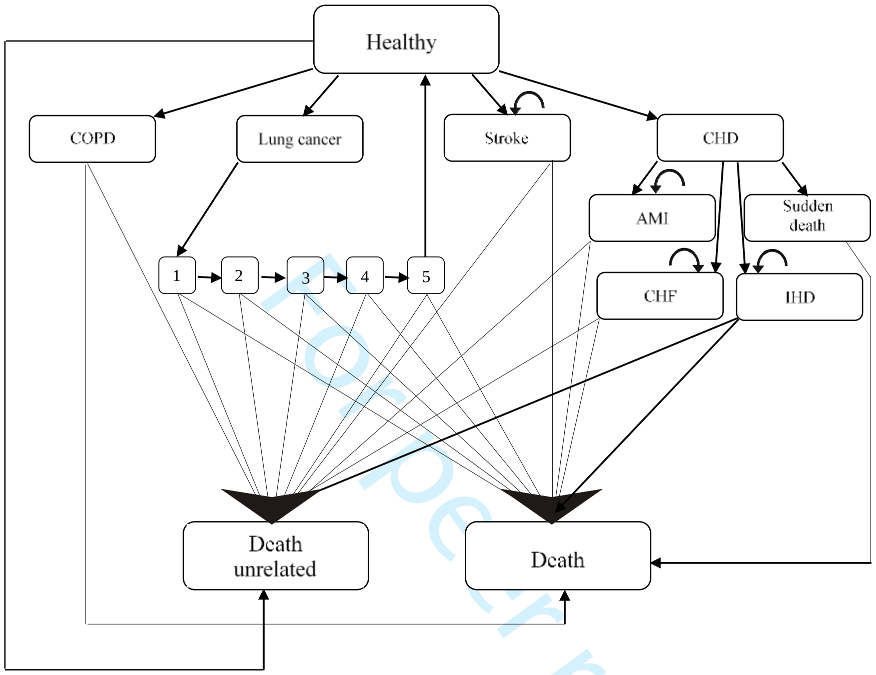

Figure 1. State-transition diagram

The model is set up with two reward sets; costs and effects. The incremental rewards are accumulated during time spent in the health states. The transitional rewards lost life years and some costs are recorded at transitions between healthy and disease state, and disease state and death.

The Markov-cycle tree is run as a microsimulation with 10 000 repetitions. The simulation ends at death or age 95 years. The model is run separately for age and gender groups. The result of each simulation is expected value, with accompanying distributions. The two simulations, the continuing smoker and the quitter, are compared outside the model. The results are presented as expected value per individual, specific for gender, age and smoking status.

For peer review only - http://bmjopen.bmj.com/site/about/guidelines.xhtml

Material

The model is based on principles for cost-effectiveness analysis in health and medicine (Gold et al, 1996; Drummond et al, 2005) and Swedish methodological recommendations (TLV, 2004). The model holds the societal perspective, aiming to incorporate disease-specific costs for all sectors of Swedish society.

The model uses Swedish register data and secondary data from previously published scientific articles. The secondary data was found through searches in the database MEDLINE and the reference lists of retrieved articles, choosing the data that is considered most relevant to present-day Swedish circumstances and the target group. No meta-analysis nor other synthesis of data was performed.

All costs are expressed in year 2014 SEK (USD 1=SEK 6.86; Euro 1=SEK 9.10), converted if necessary by the Swedish CPI (consumer price index). The annual discount rate is 3% for both costs and health effects.

The risks

Disease risks

All disease risks are annual age- and gender-specific excess incidence risk until the age of 95 years, see tables 2 to 5.

The COPD disease risk is taken from the Swedish population-based study Obstructive Lung Disease in Northern Sweden (OLIN), which was started in year 1985 (Lundbäck et al, 1991). The risk is the reported average excess seven-year incidence among smokers in three age groups, of which the youngest was 45 years at baseline, see table 2. COPD was defined according to the spirometer GOLD definition.

Table 2. Risks COPD.

	men & women	source
Disease risk		
Risk until age 45	0%	Lindberg et al, 2006
Excess annual risk for smokers, from age 46	1.6%	
Effect of quitting		
Risk fraction for quitters, years since quitting:		Inspired by Surgeon General, 1990
0-5	1	
6-15	0.5	
16-24	0.3	
>25	0.1	
Death risk		
Excess risk among diseased, as fraction of age-specific general death risk, by age:		Estimated from Lundbäck et al, 2009<br>Statistics Sweden, database
<58 years	1	
58-70 years	5	
>70 years	1	

For peer review only - http://bmjopen.bmj.com/site/about/guidelines.xhtml

For peer review only

Table 3. Risks lung cancer.

	men	women	source
Death risk			
Accumulated death risk until age 75			
Smokers	16.7%	10.4%	Peto et al, 2000
Non-smokers	0.4%	0.4%	
Risk for ages <40	0	0	Assumed, based on Peto et al, 2000
Smokers accumulated excess death risk until age 95	37.2%	23.1%	Interpolated, based on Peto et al, 2000
Age-adjusted conditional death risk	see table 8		
Disease risk			
Smokers accumulated excess disease risk until age 95	42.0%	26.3%	After interpolation, based on Peto et al, 2000 and Holm et al, 1995
Effect of quitting			
Risk fraction for quitters, years since quitting:			Peto et al, 2000
<10	0.66	0.69	
10-19	0.42	0.21	
20-29	0.18	0.05	
30-35	0.08	0	
>36	0	0	

The lung cancer disease risk is estimated from reports on lung cancer deaths until age 75 for smokers (15-24 cigarettes/day) and non-smokers, see table 3. The annual excess death risk is estimated by a quadratic function of the accumulated risk until age 75 years. The lung cancer death risk is assumed 0 until the age of 40 years, and assumed constant between ages 75 and 95. The disease risk is obtained by adjusting the annual death risk by the annual crude survival rate of lung cancer in Sweden for a similar time period as the Peto data, from Holm et al (1995).

Table 4. Risks CHD and stroke.

	men & women	source
Disease risk	Framingham, see tables 5-7	
Effect of quitting		
Risk fraction for quitters, years since quitting:		Surgeon General, 1990
on CHD:		
1	0.5	
>15	0	
on stroke:		
>10	0	
Death risk		
AMI, 1st year	see table 9	
Stroke, 1st year	see table 10	
CHF	see table 11	
Risks as fraction of age- and gender-specific general death risk:		Statistics Sweden
AMI, 2nd and following years, age 15-93 years	3	Henriksson et al, 2014
AMI, 2nd and following years, age >93 years	2	Assumed
Stroke, 2nd and following years, age 15-93 years	3	Henriksson et al, 2014
Stroke, 2nd and following years, age >93 years	2	Assumed
IHD, 1st year	2.5	Granström et al, 2012
IHD, 2nd and following years	2.15	Granström et al, 2012

For peer review only - http://bmjopen.bmj.com/site/about/guidelines.xhtml

Table 5. The annual risks of CHD.

μ_{chd} =5.5305+28.4441*Sex-1.479*Ln(Age)-14.4588*Ln(Age)*Sex+1.8515*(Ln(Age))²*Sex-0.9119*Ln(SBP)-0.2767*Smok-0.7181*Ln(Chol/HDL)-0.1759*Diabetes-0.1999*Diabetes*Sex

P_{chd} =1-Exp(-Exp((- μ_{chd})/ Exp(0.9145-0.2784* μ_{chd})))

Source: Caro et al, 2007; Anderson et al, 1991

Table 6. The annual risks of stroke.

μ_{str} =26.5116+0.2019*Sex-2.3741*Ln(Age)-2.4643*Ln(SBP)-0.3914*Smok-0.0229*Ln(Chol/HDL)-0.3087*Diabetes-0.2627*Diabetes*Sex

P_{str} = 1-Exp(-Exp((- μ_{str})/ Exp(-0.04312* μ_{str})))

Source: Caro et al, 2007; Anderson et al, 1991

The CHD and stroke disease risk estimates are based on the Framingham CVD risk function, see table 4 and tables 5-6. As the Framingham CHD risk function only calculates CHD events, the division of these events into the particular diseases are based on recent Swedish register data, see table 7. To avoid over-estimation of risks, the risk factors for CHD and stroke are evaluated at minimal-risk levels; 120 mmHg for systolic blood pressure (SBP), HDL-cholesterol (HDL) at 1.5 and cholesterol (Chol) at 4. Diabetes is set at 0, while the variable smoking (smok) is set at 1 for the smokers.

Table 7. Distribution of diseases within CHD.

	Age < 65 years		Age >65 years	
	men	women	men	women
AMI	0.42	0.40	0.31	0.31
IHD	0.40	0.39	0.21	0.29
CHF	0.16	0.19	0.46	0.38
Sudden death	0.02	0.02	0.02	0.02

Source: Swedish National Board of Health and Welfare, Statistics database, Diagnoses in inpatient care from the Hospital Discharge Register, year 2013.

For peer review only - http://bmjopen.bmj.com/site/about/guidelines.xhtml

Table 8. Death risk lung cancer.

Age group	Years since diagnosis 1	2	3	4	5
0-54	0.550	0.172	0.034	0.034	0.034
55-74	0.610	0.168	0.030	0.030	0.030
75-95	0.743	0.120	0.021	0.021	0.021

Source Based on Talbäck et al, 2004

Death risks

All death risks are age-and gender disease-specific conditional risks; in some cases estimated as fractions of the general population age- and gender-specific mortality risk, see tables 2 to 4, and in some cases based on Swedish register data, see tables 8 to 11.

The COPD death risk is estimated from the study Obstructive Lung Disease in Northern Sweden (OLIN), which reported the 20-year mortality in three age groups. Comparison with the general age-specific mortality risks revealed no excess risk of death among those younger than 58 years and older than 70 years, but a considerable increased risk among those aged 58-70 years at follow-up. The excess risk was estimated at on average around 5 times the age- and gender-specific general population death risk, see table 2.

The lung cancer death risk is based on survival data from the Swedish National Cancer Registry, see table 8. The death risks for year 3 and 4 after diagnosis are estimated by linear interpolation between years 2 to 5. Lung cancer survivors at 5 years are assumed recovered, and returned to the health state healthy.

The death risks from CHD and stroke are taken from Swedish registers, see tables 9 to 11, or published scientific reports, see table 5. The death risks for AMI, stroke and IHD are divided into risks the first year after the first event and the second and following years after first event.

Table 9. Death risk AMI, 1st year.

Age group	men	women
20-49	0.077	0.077
50-64	0.137	0.101
65-69	0.159	0.149
70-74	0.172	0.141
75-79	0.206	0.191
80-84	0.255	0.224
>84	0.327	0.331

Source: Swedish National Board of Health and Welfare, The Swedish AMI Statistics, year 2013

For peer review only - http://bmjopen.bmj.com/site/about/guidelines.xhtml

Table 10. Death risk stroke, 1st year.

Age group	men	women
20-49	0.031	0.038
50-54	0.059	0.051
55-59	0.044	0.064
60-64	0.046	0.061
65-69	0.062	0.066
70-74	0.077	0.085
75-79	0.097	0.109
80-84	0.148	0.157
>84	0.216	0.257

Source: Swedish National Board of Health and Welfare. The Swedish Stroke Statistics, year 2013

Table 11. Death risk CHF.

Age group	men	women
15-49	0	0
50-69	0.057	0.015
70-84	0.245	0.162
>84	0.340	0.281

Source: Swedish National Heart Failure Register, year 2012

The model also incorporates the possibility of dying in unrelated diseases. The death risk in the health state Healthy is the average 5-year age group- and gender-specific risk adjusted for all model disease deaths, the last column in table 12. In disease health states, the risk of dying in unrelated disease is the average 5-year age group- and gender-specific

Table 12. Death risks, unrelated.

Age Group	Not COPD		Not Lung cancer		Not AMI		Not CHF		Not IHD		Not Sudden death		Not Stroke		Not model disease	
	m	w	m	w	m	w	m	w	m	w	m	w	m	w	m	w
<39	0.001	0.000	0.001	0.000	0.001	0.000	0.001	0.000	0.001	0.000	0.001	0.000	0.001	0.000	0.001	0.000
40-44	0.001	0.001	0.001	0.001	0.001	0.001	0.001	0.001	0.001	0.001	0.001	0.001	0.001	0.001	0.001	0.001
45-49	0.002	0.001	0.002	0.001	0.002	0.001	0.002	0.001	0.002	0.001	0.002	0.001	0.002	0.001	0.002	0.001
50-54	0.003	0.002	0.003	0.002	0.003	0.002	0.003	0.002	0.003	0.002	0.003	0.002	0.003	0.002	0.003	0.002
55-59	0.005	0.003	0.004	0.003	0.004	0.003	0.005	0.003	0.004	0.003	0.005	0.003	0.005	0.003	0.005	0.003
60-64	0.008	0.005	0.007	0.005	0.007	0.005	0.008	0.005	0.007	0.005	0.008	0.005	0.008	0.005	0.008	0.005
65-69	0.013	0.008	0.012	0.008	0.012	0.009	0.013	0.009	0.012	0.009	0.013	0.009	0.013	0.009	0.013	0.008
70-74	0.021	0.013	0.020	0.013	0.019	0.014	0.021	0.014	0.020	0.013	0.021	0.014	0.020	0.014	0.021	0.013
75-79	0.037	0.023	0.036	0.023	0.035	0.024	0.037	0.024	0.035	0.023	0.038	0.024	0.036	0.023	0.037	0.023
>79	0.068	0.047	0.068	0.047	0.065	0.047	0.068	0.047	0.065	0.046	0.071	0.048	0.068	0.046	0.068	0.047

m=men, w=women

Source: Swedish National Board of Health and Welfare. The Swedish National Causes of Death Register, year 2014

For peer review only - http://bmjopen.bmj.com/site/about/guidelines.xhtml

risk adjusted for the deaths in each respective disease. For ages below 39 years the risk in the age group 35-39 years is used, and for ages 80-84 years the risk >79 years. For ages above 84 years, the general population age-and gender specific death risk is used for the unrelated death risk. As the lung cancer death risks are so high, the unrelated death risks for lung cancer among individuals aged above 84 years had to be adjusted, by deducting 0.05. For those aged below 85 years, the age- and gender-specific general population risk of death is only used for calculating some disease-specific death risks, see tables 2 and 4. The risk is taken from the Swedish national mortality statistics for the year 2014 (Statistics Sweden, 2015).

Changes in risk after quitting smoking

The excess disease risks for smokers are not eliminated immediately after quitting smoking. This "lead time" is 36 years for lung cancer, 16 years for CHD, and 11 years for stroke, while for COPD some excess risk remain life-long, see heading effect of quitting in tables 2 to 4. The disease risks after quitting are constructed by adjusting the smokers' risks by the remaining risk. The remaining risk is modelled as fractions of risk, given the number of years since quitting. The annual remaining risks are estimated by linear interpolation. The effects on the risk for CHD and stroke are modelled on the dummy variable smoking, adjusting the value of 1 by the remaining risk fraction.

The societal costs

The model is reflecting the societal perspective, including disease-related costs for all sectors of the Swedish society. The costs included are medical treatment costs, costs for institutional care and technical aids, pharmaceutical costs, informal care and other patient and relatives' costs, and morbidity productivity costs.

Most of the data on societal costs are taken from Swedish studies published during the 2010s. Data reported as distributions, i.e. with the Gamma parameters for costs, or bootstrapped 95 percent confidence interval were preferred and used in the model to

Table 13. Medical treatment costs. SEK 2014.

	mean	95% confidence interval	distribution	source	comment
Lung cancer	76 096	-	-	KPP register, SALAR 2015	Only inpatient care
COPD	10 120	6 120 - 14 920	-	Jansson et al, 2013	Moderate COPD
AMI year 1	171 660	-	Gamma 106;1622	Henriksson et al, 2014	
AMI year 2+	45 740	-	Gamma 17;2698	Henriksson et al, 2014	
CHF	33 850	-	-	Agvall et al, 2005	
IHD	51 610	-	-	Mourad et al, 2013	Angina pectoris
Stroke year 1	142 280	-	Gamma 114;1244	Henriksson et al, 2014	
Stroke year 2+	38 450	-	Gamma 48;800	Henriksson et al, 2014	

For peer review only - http://bmjopen.bmj.com/site/about/guidelines.xhtml

enable stochastic estimation. If data was reported as mean and standard deviation, the Gamma distribution was simulated employing the Treeage function. In one case, data was reported as fraction of patients consuming a specific resource, which was used for sampling within the model. Otherwise the reported point estimate, usually the average cost across the patient group, was used. If no Swedish data on a cost item was found, the cost was taken from studies reporting data from settings assumed similar to the Swedish.

All costs are reported in SEK year 2014 (USD 1=SEK 6.86; Euro 1=SEK 9.10), adjusted when necessary with the Swedish CPI. To adjust reported Gamma distributed parameters to the price level, only the second parameter, i.e. the scale parameter, was adjusted.

Medical treatment costs

Recent Swedish estimates on medical treatment costs were possible to obtain for all model diseases, see table 13. The costs are paid by the regional healthcare authorities.

Institutional care and technical aids costs

These costs include rehabilitation, terminal care, old age homes, support for individuals living at home, transportation and technical aids. In Sweden, institutional care and technical aids used by patients in their homes are the responsibility of the local authorities (municipalites, in Swedish: kommuner). The costs are not fully represented for any disease, see table 14. Estimates are not available for lung cancer and the only available costs for IHD are outdated, so the institutional care costs are probably underestimated.

Table 14. Costs for institutional care and technical aids. SEK 2014.

	mean	95% confidence interval	distribution	source	comment
Lung cancer	0	-	-		
COPD	0	-	-		Oxygen theraphy included in medical treatment costs
AMI year 1	16 680	-	Gamma 11;1502	Henriksson et al, 2014	Home care and nursing home
AMI year 2+	8 340	-	Gamma 11;751	Henriksson et al, 2014	Home care and nursing home
CHF	2 200	-	-	Agvall et al, 2005	Nursing home
IHD, age <65	3 140	-	-	Andersson & Kartman, 1995	Social services and aids, angina pectoris
IHD, age >64	8 260	-	-	Andersson & Kartman, 1995	Social services and aids, angina pectoris
Stroke year 1	82 130	-	Gamma 11;7184	Henriksson et al, 2014	Home care and nursing home
Stroke year 2+	41 070	-	Gamma 11;3593	Henriksson et al, 2014	Home care and nursing home

For peer review only - http://bmjopen.bmj.com/site/about/guidelines.xhtml

Table 15. Pharmaceutical costs. SEK 2014.

	mean	95% confidence interval	distribution	source	comment
Lung cancer	0	-	-		
COPD	0	-	-		included in medical treatment costs
AMI year 1	11 960	-	-	Mourad et al, 2013	
AMI year 2+	9 250	-	-	Mourad et al, 2013	
CHF	8 420	-	-	Agvall et al, 2005	
IHD	12 690	-	-	Mourad et al, 2013	Angina pectoris
Stroke year 1	2 120	-	-	Ghatnekar et al, 2013	
Stroke year 2+	2 820	-	-	Ghatnekar et al, 2013	

Pharmaceutical costs

Costs for pharmaceuticals in Sweden ought to be divided between the county councils and the patients, as patients pay a considerable share in co-payment. This is however not possible, given the data available. Table 15 therefore presents the drug costs to the regional healthcare authorities. The costs of pharmaceuticals dispensed during hospital stays are included in the medical treatment costs.

Informal care and other patient and relatives' costs

These costs include the value of care given to patients by relatives and other costs for patients or relatives, such as time, co-payments paid for health care and drugs as well as costs for transportation, modifications at home etc. Complete estimates could not be obtained for any disease, see table 16, except IHD which however might be outdated. Informal care in present-day Sweden probably constitute a sizeable part of total societal costs.

Table 16. Informal care and other patient and relatives' costs. SEK 2014.

	Mean	95% confidence interval	distribution	source	comment
Lung cancer	140 810	-	-	Gridelli et al, 2007	Informal care, estimated from 3 months home care
COPD	0	-	-		
AMI year 1	2 090	-	Gamma 44;48	Henriksson et al, 2014	Informal care
AMI year 2+	1 050	-	Gamma 44;24	Henriksson et al, 2014	Informal care
CHF	0	-	-		
IHD, age <65	5 180	-	-	Andersson & Kartman, 1995	Travel and time costs for healthcare contacts, angina pectoris
IHD, age 65+	2 500	-	-	Andersson & Kartman, 1995	Travel and time costs for healthcare contacts, angina pectoris
IHD	680	-	-	Andersson & Kartman, 1995	Informal care, angina pectoris
Stroke year 1	28 260	-	Gamma 44;636	Henriksson et al, 2014	Informal care
Stroke year 2+	14 130	-	Gamma 44;308	Henriksson et al, 2014	Informal care

For peer review only - http://bmjopen.bmj.com/site/about/guidelines.xhtml

Table 17. Productivity costs, morbidity. SEK 2014.

	mean	95% confidence interval	sd	distribution	source	comment
Lung cancer	0	-	-	-	Ford et al, 1999	Simulated in model: 9% of pat. 100% disability 20% of pat. 80% disability 40% of pat. 50% disability 31% of pat. 20% disability
					Statistics Sweden	Age- and gender-specific mean wages year 2014
COPD	21 800	6 011 - 42 583	-	-	Jansson et al, 2013	Moderate COPD
AMI year 1	38 180	-	-	Gamma 9;4242	Henriksson et al, 2014	
AMI year 2+	19 090	-	-	Gamma 9;2121	Henriksson et al, 2014	
CHF	29 880	-	49 210	-	Zethraeus et al, 1999	Difference year before and after disease onset
IHD	121 020	-	99 880	-	Mourad et al, 2013	Angina pectoris
Stroke year 1	194 100	-	-	Gamma 9;21567	Henriksson et al, 2014	
Stroke year 2+	97 050	-	-	Gamma 9;10783	Henriksson et al, 2014	

Productivity costs

The productivity costs only value the lost production because of morbidity before the age of 66 years, not mortality. The productivity costs for lung cancer is simulated within the model, via sampling from the fraction of patients on sick leave and combined with age- and gender-specific average monthly wages, including 40% employer taxes. Remaining data is taken from the literature, see table 17, and most estimates are recent. The costs are valued by the human capital method, and thus only include losses in salaried work before the official age of retirement.

The health effects

Life years lost

The number of life years lost (YLS) are calculated until the age of 95 years, and only for individuals dead in the modelled diseases. Life years lost are presented both discounted 3% and undiscounted.

QALYs

The number of quality-adjusted life years (QALYs) are calculated during healthy years and years spent diseased, until death or the age of 95 years.

The QoL weights used during healthy years are mean age group- and gender-specific population weights, see table 18. The data is somewhat dated, but it is the only general population QoL weights available in Sweden. The QoL of the age group 20-29 years is used

For peer review only - http://bmjopen.bmj.com/site/about/guidelines.xhtml

Table 18. Average Swedish population QoL weights.

Age group	men	women
20-29	0.91	0.88
30-39	0.90	0.86
40-49	0.86	0.85
50-59	0.84	0.82
60-69	0.83	0.78
70-79	0.81	0.78
80-88	0.74	0.74

Source: Burström et al, 2001

For peer review only

also for younger ages, and the QoL of the age group 80-88 years is used for those aged 89-95 years. This last assumption is probably an overestimate.

The disease-specific QoL used in the health states are all, except one, modelled as decrements from the average population age-group and gender-specific QoL, see table 19. For lung cancer no data was available on the marginal effect of the disease on the population average QoL, so a fixed value over the ages and genders had to be used.

Sensitivity analyses

Several univariate and multivariate sensitivity analyses have been performed. Analyses on some methodological issues, as well as a probabilistic sensitivity analysis, have also been performed. The analyses are reported for men and women aged 50 years.

To give another measure of the uncertainty surrounding the cost-effectiveness ratio, the 95% confidence interval for the difference between smokers and quitters is reported, calculated from the standard deviation of outcomes.

Table 19. QoL weights and QoL decrements due to disease.

	QoL	source
Health state weight		
Lung cancer	0.653	Nafees et al, 2008
Decrement from average QoL		
COPD	0.0142	Sullivan et al, 2005
AMI	0.0627	Henriksson et al, 2014
CHF	0.0700	Granström et al, 2012
IHD	0.0900	Granström et al, 2012
Stroke	0.1384	Henriksson et al, 2014

For peer review only - http://bmjopen.bmj.com/site/about/guidelines.xhtml

Univariate analyses

Univariate analyses have been performed on all model parameters:

A. disease risks: +100%, -50%

B. death risks: +-10%. (As the unrelated death risks for those aged over 84 years are so high they had to be adjusted by deducting 0.05 for the diseases stroke, IHD and AMI, and omitted for lung cancer, to enable the simulation.)

C. risk fractions of disease after quitting: +-0.1

D. all disease costs: +-25%

E. QoL weights: QoL weight 1 during healthy years

Multivariate analyses

Two sets of multivariate analyses have been performed:

F. high risk – low risk: death risks +100%, disease risks +10% and risk fractions +0.1 *vs* death risks -50%, disease risks –10% and risk fraction –0.1

G. high risk, high costs – low risk, low costs: death risks +100%, disease risks +10%, risk fraction +0.1 and all costs +25% *vs* death risks -50%, disease risks –10%, risk fractions –0.1 and all costs –25%

Analyses on methodological issues

Three analyses have been performed on methodological issues:

H. discount rate: 5%, 0%

I. perspective: healthcare and personal social services perspective (UK NICE perspective); excludes informal care and other patient and relatives' costs and productivity costs

J. recent Swedish data: only includes data from a Swedish context from year 2005 onwards. Excludes the data from Andersson & Kartman (1995) on institutional care and patient and relatives' costs for IHD, from Gridelli et al (2007) on lung cancer patient and relatives' care, from Ford et al (1999) for lung cancer productivity costs and from Zethraeus et al (1999) on CHF productivity costs

Probabilistic analysis

A bootstrap sampling was performed using the smoker and quitter Monte Carlo simulations of 10 000 runs. A sample of 1 000 from each simulation was drawn, with replacement, performed in Microsoft Excel. The mean of the difference in costs and QALYs between smokers and quitters was then calculated. This was replicated 1 000 times. The bootstrap is represented as a scatterplot in the cost-effectiveness plane.

For peer review only

For peer review only - http://bmjopen.bmj.com/site/about/guidelines.xhtml

Results

In this chapter, the model estimates of QALYs, YLS and societal costs are presented for men and women in some selected ages, mainly for validation purposes. More detailed simulation outcomes as well as the results of the sensitivity analyses are presented for men and women at age 50 years. Model estimates can be obtained for men and women for all ages between 15 and 95 years.

The model estimates

In table 20 the simulation results for QALYs (quality-adjusted life-years) experienced until the age of 95 years are presented, for the selected ages 15, 30, 50 and 70 years at the start of the simulations. As can be expected, the number of QALYs are highest in the younger age groups, and somewhat higher for women in most age groups. In the selected age groups, the differences between smokers and quitters are at a maximum at age 30; 0.68 for females and 0.81 for males. The confidence intervals, calculated via the mean and standard deviation (sd) from the 10 000 model runs, indicate that there are differences in QALYs between smokers and quitters.

The YLS (life-years saved) lost before the age of 95 years are presented in tables 21 and 22, discounted 3% and undiscounted. The differences in discounted YLS between smokers and quitters are somewhat higher than the differences in QALYs. The undiscounted YLS in table 22 show the number of years that smokers and quitters are expected to lose before the age of 95 years. For the ages 15, 30, and 50 the number of lost life-years is estimated at around 6 years for women smokers and 9 years for men, implying that the female smokers are estimated to live until age 89 and the male until age 86. In the oldest age group presented here, age 70, the number of lost life-years are only 1-2 years. The quitters are estimated to lose considerably fewer life-years; 1-4 years for the women and 3-5 years for

Table 20. QALYs, until age 95 years, discounted 3%.

age	smoker		quitter		difference			
	mean	sd	mean	sd	mean	95% CI		
women								
15	23.20	2.26	23.70	2.28	0.50	0.44	-	0.57
30	20.02	2.85	20.71	2.82	0.68	0.60	-	0.76
50	14.15	4.19	14.76	4.15	0.61	0.49	-	0.73
70	8.24	3.75	8.50	3.82	0.26	0.16	-	0.37
men								
15	23.21	2.84	23.83	2.70	0.63	0.55	-	0.70
30	19.65	3.20	20.46	3.19	0.81	0.72	-	0.90
50	13.18	4.34	13.95	4.47	0.77	0.65	-	0.89
70	6.78	3.61	7.15	3.76	0.37	0.27	-	0.48

For peer review only - http://bmjopen.bmj.com/site/about/guidelines.xhtml

Table 21. Life years lost (YLS), before age 95 years. Discounted 3%.

age	smoker		quitter		difference			
	mean	sd	mean	sd	mean	95% CI		
women								
15	0.97	1.90	0.23	0.87	0.74	0.70	-	0.78
30	1.55	3.02	0.51	1.83	1.04	0.97	-	1.11
50	2.35	4.82	1.49	4.09	0.86	0.74	-	0.99
70	1.22	3.31	0.92	2.98	0.30	0.22	-	0.39
men								
15	1.42	2.25	0.43	1.21	0.99	0.94	-	1.04
30	2.18	3.44	0.79	2.15	1.40	1.32	-	1.48
50	3.51	5.57	2.09	4.69	1.41	1.27	-	1.56
70	2.22	4.30	1.68	3.94	0.53	0.42	-	0.65

the men. As expected, the difference between smokers and quitters diminish with age, with a maximum at around 5 years for the females and around 6 years for the males at age 15.

The societal costs estimated for the smokers and quitters for the selected age groups are presented in table 23. The highest costs are found for age 50; 200 000 SEK and 250 000 SEK for the smokers and 130 000 and 170 000 for the quitters, in both cases higher among the men. The highest difference between smokers and quitters is however found at age 30, with a difference of 100 000 among the females and 120 000 among the males. The difference among the eldest, at age 70, is around 20 000 SEK. These cost differences reflect the amount that tobacco cessation interventions could spend on achieving one quitter and still be cost-saving.

Table 22. Life years lost (YLS), before age 95 years. Undiscounted.

age	smoker		quitter		difference			
	mean	sd	mean	sd	mean	95% CI		
women								
15	6.46	11.80	1.68	5.86	4.78	4.52	-	5.04
30	6.58	11.93	2.22	7.25	4.37	4.09	-	4.64
50	5.67	10.94	3.55	9.19	2.12	1.84	-	2.40
70	1.97	5.18	1.47	4.64	0.50	0.37	-	0.64
men							-	
15	9.25	13.51	3.05	7.89	6.20	5.89	-	6.50
30	9.21	13.39	3.51	8.68	5.70	5.39	-	6.02
50	8.42	12.57	5.01	10.53	3.40	3.08	-	3.73
70	3.56	6.70	2.68	6.11	0.87	0.70	-	1.05

For peer review only - http://bmjopen.bmj.com/site/about/guidelines.xhtml

Table 23. Societal costs. In SEK 2014 and discounted 3%.

age	smoker		quitter		difference			
	mean	sd	mean	sd	mean	95% CI		
women								
15	113 097	278 446	40 761	207 879	72 337	65 526	-	79 147
30	170 047	386 905	71 569	293 477	98 478	88 960	-	107 996
50	201 760	415 452	133 902	366 313	67 858	57 002	-	78 714
70	85 818	189 827	63 824	171 358	21 994	16 981	-	27 006
men								
15	145 233	320 143	54 148	227 222	91 085	83 390	-	98 779
30	216 626	453 147	92 782	349 085	123 844	112 632	-	135 055
50	254 279	484 787	168 598	434 603	85 681	72 920	-	98 442
70	101 358	188 991	80 927	184 794	20 431	15 250	-	25 611

Selected model outcomes

The underlying estimated disease outcome is presented in figures 2 and 3, for the age 50 years. For both women and men, there is a marked decrease for quitters in the number of diseased and dead in the model diseases, which is somewhat offset by an increase in the number of deaths in unrelated diseases. The number of diseased and deaths are higher for

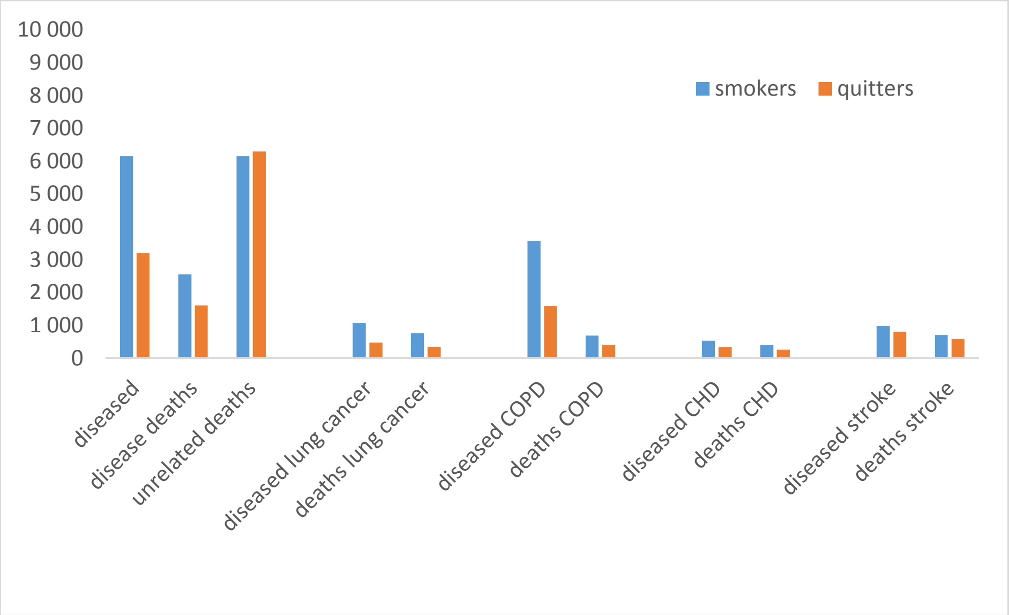

Figure 2. The disease outcome, number of diseased and dead per 10 000 for smokers and quitters, women aged 50 years.

For peer review only - http://bmjopen.bmj.com/site/about/guidelines.xhtml

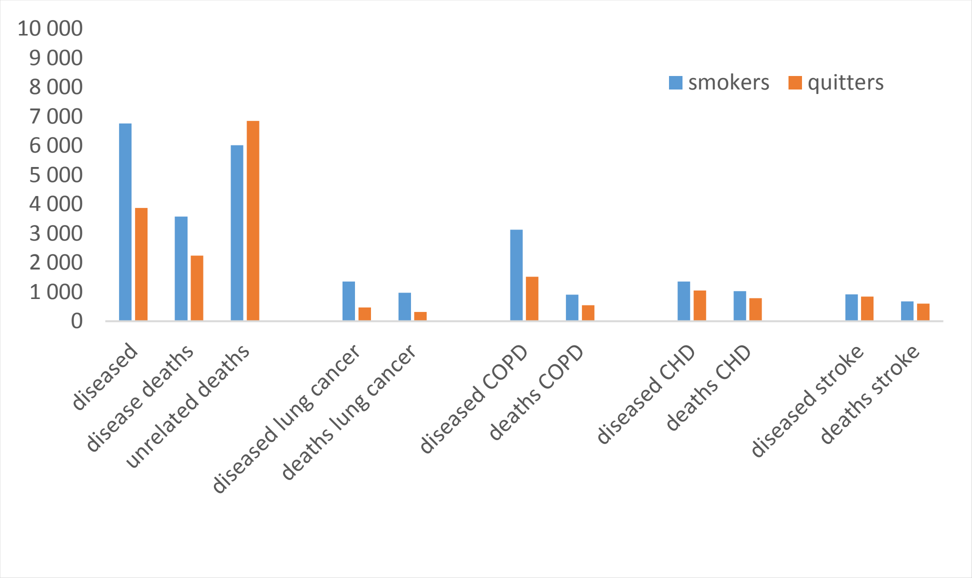

Figure 3. The disease outcome, number of diseased and dead per 10 000 for smokers and quitters, men aged 50 years.

men, mainly originating from CHD. The model disease with the highest smoking-related incidence is COPD, for both genders. The increase in unrelated deaths for the quitters is an example of competing risks, which decreases the difference in life-years and QALYs between smokers and quitters.

Table 24 and 25 shows the full model simulation results of the societal cost savings because of tobacco quitting at age 50 years. For women, the highest estimated savings are found in lung cancer, COPD and stroke at around 15-20 000 SEK per quitter. For men the cost savings because of lung cancer are considerable higher, at around 35 000, due to the higher incidence among the men. The cost item with the largest cost savings are medical treatment costs for both genders, at around 30 000 SEK. Most of the difference in savings between men and women originate from the productivity costs, possibly reflecting disease onset at younger ages among men. Note that a cost saving of zero means that no cost is being modelled, as cost data was lacking.

Table 24. Societal cost savings, in SEK 2014. Women aged 50 years.

	Lung cancer	COPD	AMI	CHF	IHD	Stroke	Sum
Medical treatment	5 171	13 573	2 337	439	3 410	5 500	30 430
Institutional care and technical aids	0	0	365	29	408	4 880	5 681
Pharmaceuticals	0	0	361	109	838	306	1 615
Informal care and other patient and relatives' costs	9 569	0	44	12	282	1 673	11 580
Productivity costs	3 971	6 456	192	243	3 228	4 462	18 552
Sum	18 711	20 029	3 300	832	8 166	16 821	67 858

For peer review only - http://bmjopen.bmj.com/site/about/guidelines.xhtml

Table 25. Societal cost savings, in SEK 2014. Men aged 50 years.

	Lung cancer	COPD	AMI	CHF	IHD	Stroke	Sum
Medical treatment	8 477	11 478	3 203	596	4 738	3 907	32 399
Institutional care and technical aids	0	0	456	39	596	3 379	4 470
Pharmaceuticals	0	0	473	148	1 165	214	2 000
Informal care and other patient and relatives' costs	15 685	0	59	16	377	1 164	17 301
Productivity costs	13 002	8 357	319	400	3 785	3 649	29 511
Sum	37 164	19 835	4 510	1 199	10 661	12 312	85 681

Sensitivity analyses

The results of the sensitivity analyses are presented on women and men at starting age 50 years. Figure 4 shows the results for women and figure 5 for men.

All analyses show a similar pattern between men and women, and also similar ranges. The univariate sensitivity analyses on the model parameters, analyses A to E, result in small changes in costs and QALYs. Also the multivariate analyses F and G, which are constructed as scenarios that allow the risk parameters to vary consistently upwards or downwards, and along with the costs in analysis G, show moderate changes from the base case estimates. The methodological choices have a more pronounced effect, as the largest difference in QALYs is achieved by varying the discount rate (analysis H) between 0 and 5%, which also affects the costs substantially. The two analyses that reflect the choices of which costs to include in the estimates, analysis I that reflects the UK NICE health care and social services perspective and analysis J that only include Swedish data published since the year 2005, both decrease the cost differences between smokers and quitters.

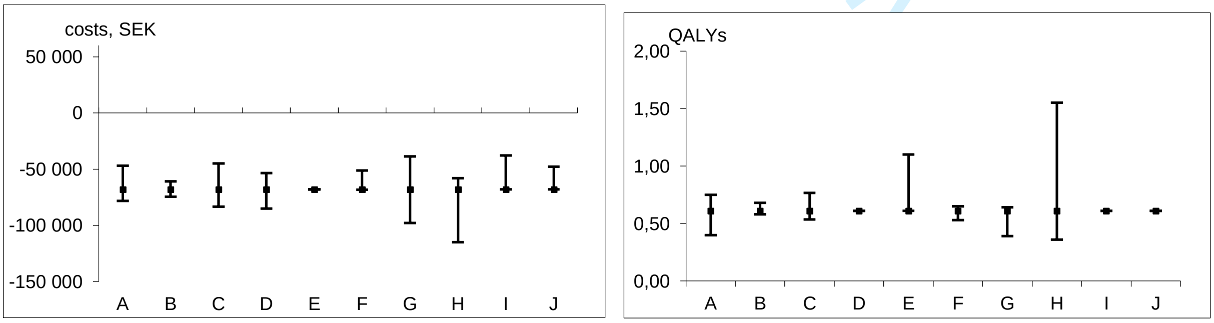

Figure 4. Sensitivity analyses on societal cost and QALY differences between smokers and quitters, women aged 50 years.

Notes: **A**. disease risks. **B**. death risks. **C**. risk fractions of disease after quitting. **D.** all costs. **E**. QoL weights. **F**. high risk – low risk. **G**. high risk, high costs – low risk, low costs. **H**. discount rate. **I**. perspective. **J.** recent Swedish data.

For peer review only - http://bmjopen.bmj.com/site/about/guidelines.xhtml

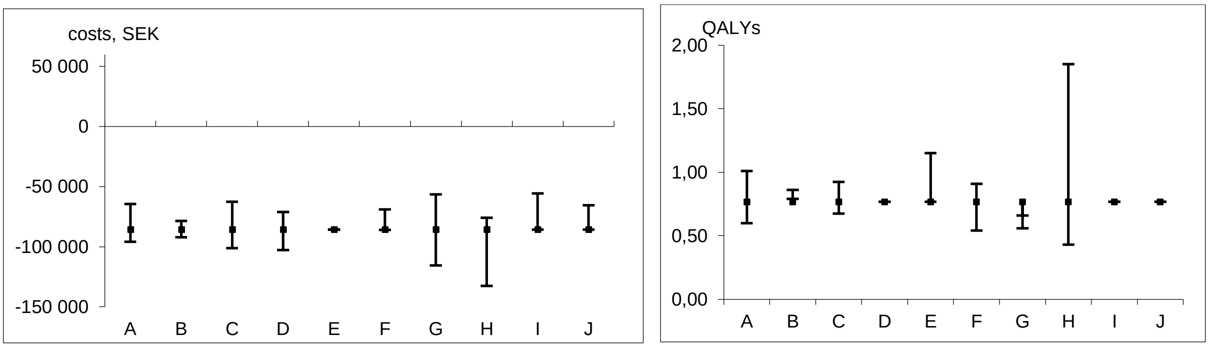

Figure 5. Sensitivity analyses on societal cost and QALY differences between smokers and quitters, men aged 50 years.

Notes: **A**. disease risks. **B**. death risks. **C**. risk fractions of disease after quitting. **D.** all costs. **E**. QoL weights. **F**. high risk – low risk. **G**. high risk, high costs – low risk, low costs. **H**. discount rate. **I**. perspective. **J.** recent Swedish data.

For peer revi

The scatter plot of the bootstrap analysis based on the microsimulation results for women and men aged 50 are shown in figures 6 and 7. The uncertainty is higher for the men, as the plots are more scattered. All plots are however situated in the cost decrease and QALY increase quadrant, with costs below -20 000 SEK and QALYs over 0.2.

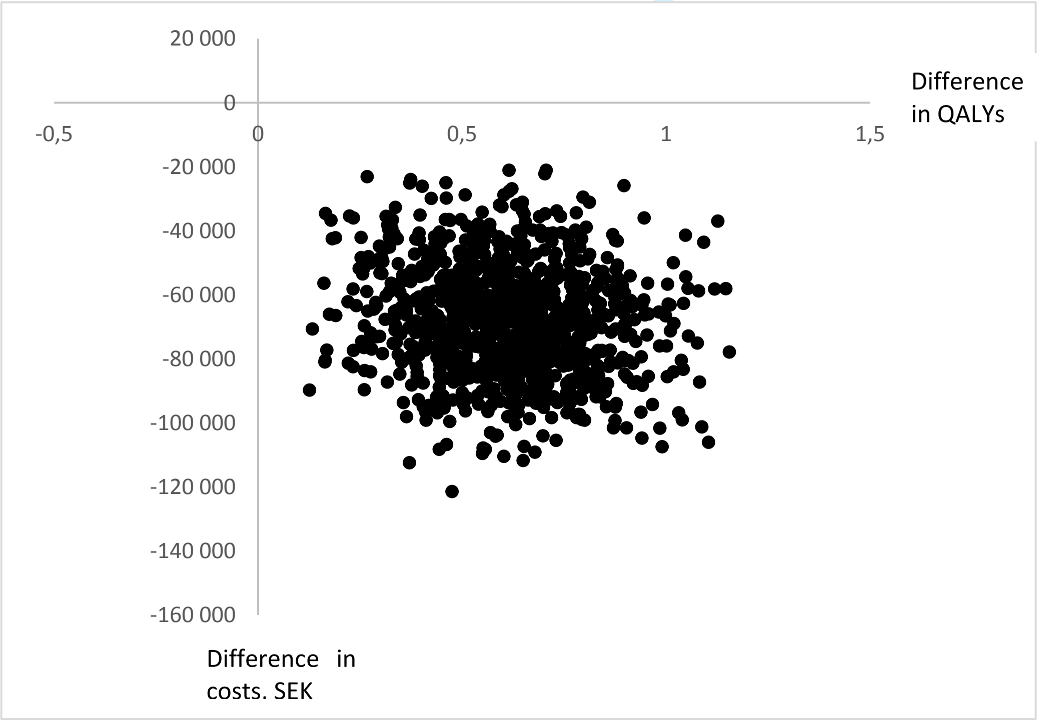

Figure 6. The cost-effectiveness plane with resultat från bootstrap, women aged 50 years.

For peer review only - http://bmjopen.bmj.com/site/about/guidelines.xhtml

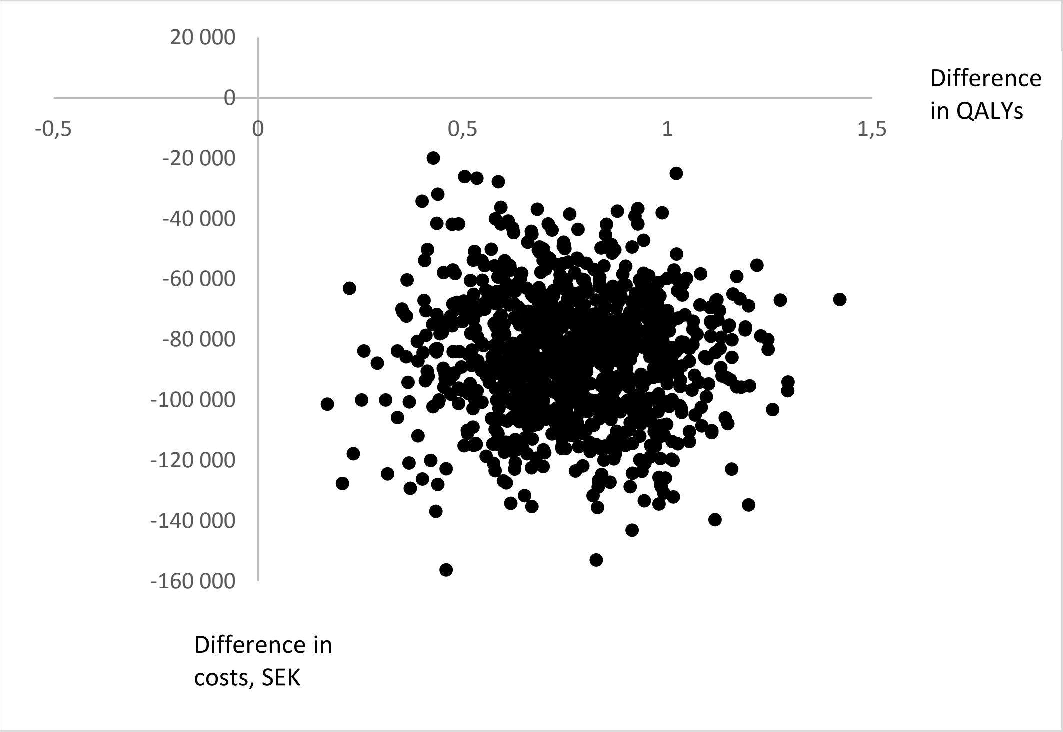

Figure 7. The cost-effectiveness plane with resultat from bootstrap, men aged 50 years.

For peer review only - http://bmjopen.bmj.com/site/about/guidelines.xhtml

Discussion: Model validity

The discussion of the model validity is structured around four aspects as proposed by McCabe & Dixon (2000): the structure of the model, the inputs to the model, the results of the model and the value of the model to the decision-maker.

The structure of the model

The structure of the model is a Markov model constructed for microsimulations, on the three most smoking-related disease groups; lung cancer, COPD, and CVD including stroke and CHD. The present updated version of the model includes one less CHD disease compared to the first version of the model, as unrecognized acute myocardial infarction now is included in the IHD disease, mainly because the disease definition is rarely used nowadays. Choosing only three disease groups is a clear simplification as smoking is known to cause hundreds of different diseases. The effects from smoking, and thus quitting, are furthermore confined to the individuals themselves; no side-effects on other individuals such as environmental tobacco smoke or smoking uptake are included. These two features leads to an underestimate of the true effects of tobacco quitting.

The same disease-specific approach has been taken by most other tobacco cessation models (Bolin, 2012), even though some of them include more diseases, such as asthma. Another approach would be to use the overall differences in mortality between current, former, and never-smokers taken from large US studies, as some early tobacco cessation models did (Secker-Walker et al, 1997; Tengs et al, 2001). In order not to overestimate the effects of quitting tobacco, we chose to model the smoking-related risk for certain diseases instead, as it is improbable that all differences in mortality and morbidity between smokers and former smokers are due to the smoking habit (Doll et al, 1994).

The model aims to reflect disease onset related to smoking tobacco. As disease in all the three disease groups included in model may be caused by other factors than smoking only the excess risks for smokers are modelled. For the diseases lung cancer and COPD this implies that the risk for smokers found in epidemiological studies is adjusted by the risk found for non-smokers. For the disease group CHD and stroke, where a large fraction of disease onset is caused by other factors than smoking, this adjustment for smokers´ excess risk was performed by setting the other risk factors in the risk function at minimal risk levels. This is an underestimate, as the risk factor levels among smokers can be expected to be at least as elevated as among the general population. The underestimate is aggravated by the fact that the functional form of the risk function results in a multiplier effect of the risk factors.

The present version of the model includes seven health states: lung cancer, COPD, stroke, and CHD divided into four diseases. This is a clear simplification, as the costs and QoL can be expected to vary considerable between patients with different severity levels within the diseases. This is particularly true for COPD which is a chronic progressive disease, i.e. the

For peer review only - http://bmjopen.bmj.com/site/about/guidelines.xhtml

diseased get more severely ill over time. However, a model with 7 health states with accompanying disease-specific death risks, costs and QoL weights is fairly complex as well as data-demanding. For the purposes of this study´s model, the division of diseases into severity levels was not deemed necessary.

An obvious problem with the model, inherent in all Markov models, are the mutually exclusive health states; any individual can only contract one disease, and once diseased the individual never recovers (apart from the very rare 5 year survivors in lung cancer). This feature implies both an overestimate and an underestimate of the true effects. The underestimate stems from the fact that co-morbidity is very common, especially among the individuals with the chronic diseases COPD, CHD, and stroke. The overestimate of costs and effects arise as individuals stay in the health states until death. If the costs and outcomes associated with the health states are taken from severely ill individuals, then these become grossly overestimated. This overestimate is partly offset by the use of separate costs for the first and subsequent years, for all societal costs due to AMI and stroke. In order not to overestimate the numbers of years spent in disease states, the possibility of dying in unrelated diseases is present in all health states. This feature is also included in the CHD Policy Model (Weinstein et al, 1987).

Most tobacco cessation models are built for cohort estimation (Bolin, 2012), but this model is constructed for individual-level estimation using the microsimulation methodology. As the data available admitted a microsimulation structure, e.g. the risk functions, the methodology was chosen as the advantages to model and to obtain a richer data set with results that reflect the heterogeneity of outcomes between individuals was deemed to offset the disadvantages of calculation burden. The use of the software Treeage also facilitates the use of microsimulation. Age- and gender-specific estimates can thus be obtained from the model, between ages 15 and 95 years.

The model stages are one-year long, which seems accurate given the risk estimates and the long time horizon of the model. The reason for the model maximum age of 95 years is the lack of risk estimates for older ages. Some extrapolations of risk estimates to the age of 95 years indeed resulted problematic, as some disease-specific death risks expressed as multipliers of the average age-specific death risk resulted in risks above 1. Further extrapolations beyond the age of 95 years were deemed unnecessary, as most of the relevant differences between smokers and quitters would have arisen by that age.

The inputs of the model

The second aspect of model validity is the inputs of the model. The model contains a large number of data taken from different sources. This is of course a threat to the internal validity of the model, shared with most models. However, the data have been chosen to reflect current Swedish circumstances. The current updated version of the model has exchanged almost all cost data, if more recent estimates were available, and all death risks to recent Swedish register data. As the number of studies on any particular data items are few, no meta-analysis or any other synthesis of data was carried out.

For peer review only - http://bmjopen.bmj.com/site/about/guidelines.xhtml

The disease risks are of course are pivotal for the result. The lung cancer disease risks are probably the best that can be obtained, from a large epidemiological study (Peto et al, 2000). The risk equation used for CHD and stroke is taken from the Framingham studies, and even though there are more recent risk scores developed from the study (D'Agostini et al, 2008), the Anderson et al (1991) risk functions are still frequently employed. The disease COPD has been the subject of a large long term epidemiological study in Sweden, The Obstructive Lung Disease in Northern Sweden (OLIN) (Lundbäck et al, 1991), which is thus the most relevant data source for the model.

In the model, there is an increased risk for a smoking-related disease remaining for some years after the tobacco cessation, in accordance with epidemiological evidence (Surgeon General, 1990; Omenn et al, 1990). The feature is also considered a marker of high quality tobacco cessation models (Bolin et al, 2012).

The majority of the cost data are taken from Swedish studies published during the 2010s. To take fully advantage of the microsimulation structure and to obtain stochastic estimates, the preferred data sources were the ones reported as distributions, i.e. as Gamma parameters or bootstrapped 95 percent confidence intervals. If no Swedish data was found, an international estimate was instead used in order to seek to represent the full societal costs. However, apart from certain cost items and for some of the diseases, the lack of data results in considerable underestimates of the true societal costs. This is particularly true in the cases of the costs for care, both institutional and informal. The institutional care could amount to considerable costs, exemplified by the costs for stroke and AMI patients, see table 14. In particular for lung cancer the lack of data results in considerable underestimates of the true disease-related costs. This is why the possible overestimate of the informal care for the disease, obtained from an Italian study, probably does not bias the overall result. To investigate the issue, one sensitivity analysis only included recent Swedish data. The analysis lead to decreases in cost savings for quitters aged 50 years of around 30%.

The QoL estimates are constructed as disease-specific decrements from the average age- and gender-specific QoL, except for lung cancer for which no QoL decrement could be found (De Geer et al, 2013). The average population age- and gender-specific QoL weights, which are certainly not 1, are also used during healthy years for the base case estimates. This means that the model assumes that an individual that avoids the smoking-related diseases is not having perfect health, but the health of an average Swede at the same age, as recommended (Gold et al, 1996).

The stated purpose of the model is to reflect the societal perspective, which for Sweden includes the morbidity productivity costs, but not the productivity costs resulting from mortality. All the model data on productivity costs value them according to the human capital approach for individuals under the age of 65, the customary Swedish age of retirement.

A full societal perspective might also include other aspects, considering that this is a model on individuals that are participating in an intervention that aims to change their lifestyle. The previous version of the tobacco cessation model, version 1 (Johansson, 2004), reported

For peer review only - http://bmjopen.bmj.com/site/about/guidelines.xhtml

sensitivity analyses that modelled some effects on the tobacco quitters, by including savings from cigarette purchases and a decreased QoL because of withdrawal effects during the first year. When that analysis was applied to an intervention, a decreased QoL during the first year was also deducted for the smokers that failed to quit, as the failure to achieve a personal goal might to lead to a decrease in QoL.

The results of the model

The third aspect of model validity is the results of the model, e.g. a comparison with reality or with other study results. A direct comparison with reality is not possible, since the model covers the ages 15-95 years, with a follow-up time of 80 years for the youngest age group.

The model estimates that around 60% of the women and 70% of the men aged 50 at the start of the simulations will contract one of the modelled diseases, and that around 50% of those will die in the diseases before the age of 95 years. The disease risks for the quitters at age 50 are not eliminated; 30-40% of them will still contract the smoking-related diseases because of remaining disease risks after quitting. As expected, the unrelated deaths increase among the quitters, in sum leading to an increase in YLS (undiscounted) of 2-3 years for those quitting at age 50, compared with continuing smokers. The increases in QALYs (discounted 3%) are smaller because of less-than-perfect health among those aged 50 years and above; 0.61 for women and 0.77 for men. The disease outcomes are fairly similar to the estimates from the previous versions of the model, but because of decreased death risks, the outcomes in terms of YLS and QALYs are considerably higher. The 2004 version of the model estimated an increased YLS of 0.93 and of 1.66 for women and men aged 50-54 years, and QALY gains of 0.36 and 0.71, respectively. The differences are due to the longer time perspective of the present version, 95 years versus 85 years, and the somewhat decreased case-fatality risk (i.e. the mortality risk among those with disease) because of improvements in medical technologies during the past decade.

Apart from increases in health, the societal cost savings because of quitting smoking are considerable. For men, the cost savings amount to around 100 000 SEK for quitters aged between 15 and 50 years, and around 70-90 000 SEK for women. Even in the age group 70 years there are estimated cost savings of around 20 000 SEK per individual quitter. This implies that substantial funds could be invested in smoking cessation interventions, and the interventions would still be cost-effective, or even cost-saving. The cost savings in the present model are considerably higher than those of the previous model, in part due to changes in price year.

Comparisons of model estimates with other models' are difficult to perform, as the time horizon, costs included, jurisdiction, and the diseases included differ. Among the recently reported model estimates (Bolin, 2012), there are two Australian models. The model developed within ACE (Bertram et al, 2007) report estimates of life-years saved that are considerable higher than the present model's; 5.7 years for men and 6.6 years for women in age group 50-54 years. That model time horizon is however 100 year, but it is unlikely

For peer review only - http://bmjopen.bmj.com/site/about/guidelines.xhtml

that the feature fully explains the difference between the model estimates. The estimates of average health care cost saved per quitter (inferred from table 3) however seems to be very similar to the present model's; around 33 000 SEK. The other Australian model, the Quit Benefits Model (Hurley & Mathews, 2007), reports considerably lower estimates of both life-years and health care costs saved, e.g. 0.1 – 0.2 YLS and QALYs saved for men and women quitters. The lower estimates, in comparison with both the present model and the ACE model, are probably partly explained by the time horizon of only ten years.

There have been two, to my knowledge, reports of tobacco cessation model estimates for Sweden, one using the Benesco model (Bolin et al, 2007) and one using an extended version of the HECOS model (Bolin et al, 2006). Comparison with those model estimates are unfortunately not possible, due to lack of reporting detail. However, estimates from the previous version of this model were fairly consistent with the HECOS model estimates (Orme et al, 2001) for Sweden, available at the time (Johansson, 2004).

The value of the model to the decision-maker

The fourth aspect of validity is the value of the model to the decision-maker. There are several models on tobacco cessation that conforms to international recommendations on how to perform cost-effectiveness analyses (Bolin, 2012). This model however reflect Swedish circumstances, with Swedish cost and QoL data, why the model might be useful for Swedish decision-makers.

We hope that the model will be used to perform economic evaluations of a range of tobacco cessation interventions. For tobacco prevention interventions, i.e. prevention of initiation of smoking, another model version, version 2, has been constructed and is available for analyses. The use of these models will in time enable incremental and marginal calculations of the cost-effectiveness of different tobacco interventions and their components and suitable target groups. The basis for decisions on which tobacco cessation and prevention interventions to implement will then be more comprehensive.

Another frequent use of models is to forecast future events. This model is not suitable for estimating what the costs of smoking will be in the future. The reason is that the model does not incorporate any adjustments of possible future developments. The risk of smoking is based on studies with follow-up periods of sometimes 30 years, which means that the risks are reflecting the smoking behaviour among smokers 30 years ago. The changes in cigarette content and in the frequency of smoking might lead to changes in disease risk in the future. Also the costs for the smoking-related diseases might change in the future, because of changes in health care technology. Another example would be the value of the morbidity productivity costs, as well as informal care, as wages and productivity often are expected to increase in the future.

Nevertheless, the model actually forecasts what the costs for smokers and quitters will be in 80 years' time, for the youngest age group. That implies that we know that the model forecasts will be wrong, but it is of minor significance as the model is constructed to be used for comparisons between two groups, smokers and quitters, thus eliminating some

For peer review only - http://bmjopen.bmj.com/site/about/guidelines.xhtml

of the biases. Furthermore, the model is constructed to be used now, for present-day decisions, which have to be based on present-day information.

The uncertainty

Another aspect of model validity is the uncertainty surrounding the model estimates.

The univariate sensitivity analyses on the model parameters (analyses A-F in figures 4 and 5 for men and women aged 50) show minor deviations from the base case result, while the multivariate analysis on costs and risks combined (analysis G) affects in particular the cost estimates. The methodological choices affect the results to a greater extent, with the discount rate (H) heavily influencing the QALYs and the more restricted perspective (I) decreasing the cost-savings. The multivariate analysis that only include higher-quality data (J) also imply decreases in the cost differences between smokers and quitters, but the difference remains substantial; around 50 000 SEK for females aged 50 years and 60 000 SEK for men, respectively. The overall conclusion from the parameter sensitivity analyses is that the QALY gains are at least 0.35 and 0.40 and the cost savings at least SEK 35 000, for female and male quitters aged 50, respectively.

The probabilistic analysis shows no uncertainty whether quitting tobacco leads to cost-savings and increases in QALYs, as all bootstraps are placed in the southeast quadrant of the cost-effectiveness plane. The bootstrap results exhibit a mixture of first and second order uncertainty, as it reflects both the probabilistic structure of the Markov model and the simulation of some parameter values (Briggs, 2000).

Another measure of uncertainty is the confidence intervals around the estimated mean differences, reported in tables 20-23. However, that measure is not fully appropriate as the large sample sizes of the Monte Carlo simulation (10 000 runs) diminishes the standard error of the mean (Briggs, 2000).

The structural uncertainty of the model, i.e. whether the results would be different if the model would have been constructed in another way, have not been studied. Alternatives to the chosen model structure could have been deterministic or discreet event simulations, more or less health states, other functional forms of risk functions, and other subgroups than men and women and five-year age-groups model results. The flaw is however shared with most tobacco quitting models (Bolin, 2012).

Checking for technical errors

The model contains a large number of trackers, i.e. variables that count events, to enable checking for technical errors. Tentative runs were executed after the introduction of every new variable, with cost items undiscounted, and the simulation results examined manually. Thus, the model has been thoroughly checked for technical errors.

For peer review only - http://bmjopen.bmj.com/site/about/guidelines.xhtml

Conclusions

The aim of this study is to develop a model predicting health and economic consequences of smoking cessation, to be used for cost-effectiveness analyses of smoking cessation interventions. The updated model strives to incorporate data that is recent, accurate and appropriate for Sweden in year 2015. The model also adhere to Swedish recommendations on how to perform cost-effectiveness analyses within the health care sector. Data is however lacking to completely fulfil these requirements. Many model parameters are based on very few studies. Some information just does not exist, at least not accessible to us.

These are issues shared with most model, however. The purpose of modelling is to assemble the most accurate information at a point of time, to enable decision-making at that particular point of time. This is in accordance with one of the fundamentals of economics: decision-making under uncertainty, which implies that decisions have to be made even if there is no full information. We hope that the model will be applied to a range of different tobacco cessation interventions, which in time will enable a more comprehensive basis for decision-making.

For peer review only

For peer review only - http://bmjopen.bmj.com/site/about/guidelines.xhtml

References

Agvall B, Borgquist L, Foldevi M, Dahlström M. Cost of heart failure in Swedish primary healthcare. Scandinavian Journal of Primary Health Care 2005;23:227-232.

Anderson KM, Odell PM, Wilson PWF, Kannel WB. Cardiovascular disease risk profiles. American Heart Journal 1991;121:293-298.

Andersson F, Kartman B. The cost of angina pectoris in Sweden. Pharmacoeconomics 1995;8:233-44.

Bertram MY, Lim SS, Wallace AL, Vos T. Costs and benefits of smoking cessation aids: making a case for public reimbursement of nicotine replacement therapy in Australia. Tobacco Control 2007; 16:255-260.

Bolin K. Economic evaluation of smoking-cessation therapies -A critical and systematic review of simulation models. Pharmacoeconomics 2012;30: 551-556.

Bolin K, Lindgren B, Willers S. The cost utility of bupropion in smoking cessation health programs. Simulation model results for Sweden. Chest 2006;129:651-660.

Bolin K, Wilson K, Benhaddi H, de Nigris E, Marbaix S, Mörk A-C, Aubin H-J. Cost-effectiveness of varenicline compared with nicotine patches for smoking cessation –results from four European countries. European Journal of Public Health 2007;19:650-654.

Briggs A, Sculpher M. An introduction to Markov modelling for economic evaluation. Pharmacoeconomics 1998;13:397-409.

Briggs AH. Handling uncertainty in cost-effectiveness models. Pharmacoeconomics 2000;17:479-500.

Burström K, Johannesson M, Diderichsen F. Swedish population health-related quality of life results using the EQ-5D. Quality of Life Research 2001;10:621-635.

Caro JJ, O´Brien JA, Hollenbeak CS, Spackman E, Ben-Joseph R, Okamoto LJ, Paramore LC. Economic burden and risk of cardiovascular disease and diabetes in patients with different cardiometabolic risk profiles. Value in Health 2007;10:S12-S20.

D'Agostino RB, Vasan RS, Pencina MJ, Wolf PA, Cobain M, Massaro JM, Kannel WB. General cardiovascular risk profile for use in primary care: The Framingham Heart Study. Circulation 2008;117:743-753.

De Geer A, Eriksson J, Finnern HW. A cross-country review of data collected on non-small cell lung cancer (NSCLC) patients in cancer registries, databases, retrospective and non-randomized prospective studies. Journal of Medical Economics 2013:16; 134–149.

Doll R, Peto R, Wheatley K, Gray R, Sutherland I. Mortality in relation to smoking: 40 years' observation on male British doctors. BMJ 1994;309:901-911.

Drummond MF, Sculpher MJ, Torrance GW, O´Brien B, Stoddart GL. Methods for the economic evaluation of health care programmes. (3rd edition). Oxford: Oxford University Press; 2005.

For peer review only - http://bmjopen.bmj.com/site/about/guidelines.xhtml

Ford ES, Kelly AE, Teutsch SM, Thacker SB, Garbe PL. Radon and lung cancer: A cost-effectiveness analysis. American Journal of Public Health 1999;89:351-357.

Ghatnekar O, Asplund, K, Glader E-K, Persson U. Costs for stroke in Sweden 2009 and developments since 1997. International Journal of Technology Assessments in Health Care 2014;30:203–209.

Gold MR, Siegel JE, Russell LB, Weinstein MC, eds. Cost-effectiveness in health and medicine. New York: Oxford University Press; 1996.

Granström O, Levin L-Å, Henriksson M. Cost-effectiveness of candesartan versus losartan in the primary preventive treatment of hypertension. ClinicoEconomics and Outcomes Research 2012;4:313–322.

Gridelli C, Ferrara C, Guerriero C, Palazzo S, Grasso G, Pavese I, Satta F, Bajetta E, Cortinovis D, Barbieri F, Gebbia V, Grossi F, Novello S, Baldini E, Gasparini G, Latino W, Durante E, Giustini L, Negrini C. Informal caregiving burden in advanced non-small cell lung cancer: The HABIT study. Journal of Thoracic Oncology 2007;2:475–480.

Henriksson M, Nikolic E, Ohna A, Wallentin L, Janzon M. Ticagrelor treatment in patients with acute coronary syndrome is cost-effective in Sweden and Denmark. Scandinavian Cardiovascular Journal 2014;48:138–147.

Holm LE, Gunnarskog J, Stenbeck M. In Stenbeck M, Rosén M (eds). Cancer survival in Sweden in 1961-1991. Acta Oncologica 1995; 34(suppl 4): 39-47.

Jansson S-A, Backman H, Stenling A, Lindberg A, Rönmark E, Lundbäck B. Health economic costs of COPD in Sweden by disease severity –has it changed during a ten years period? Respiratory Medicine 2013;107:1931-1938.

Johansson P. A model for economic evaluations of smoking cessation interventions – technical report. Version 1. Centre for Public Health, Stockholm county council; 2004. Available at: http://www.publichealtheteconomics.se/wp-content/uploads/2016/06/smoking-cessation-cea-model.pdf

Lindberg A, Eriksson B, Larsson L-G, Rönmark E, Sandström T, Lundbäck B. Seven-year cumulative incidence of COPD in an age-stratified general population sample. Chest 2006;129:879–885.

Lundbäck B, Nyström L, Rosenhall L, Stjernberg N. Obstructive lung disease in northern Sweden: respiratory symptoms assessed in a postal review. European Respiratory Journal 1991; 4:257–266.

Lundbäck B, Eriksson B, Lindberg A, Ekerljung L, Muellerova H, Larsson L-G, Rönmark E. A 20-year follow-up of a population study-based COPD cohort -Report from the Obstructive Lung Disease in Northern Sweden Studies. COPD 2009;6:4,263-271.

McCabe C, Dixon S. Testing the validity of cost-effectiveness models. Pharmacoeconomics 2000;17:501-513.

Mourad G, Alwin J, Strömberg A, Jaarsma T. Societal costs of non-cardiac chest pain compared with ischemic heart disease –a longitudinal study. BMC Health Services Research 2013;13:403.

For peer review only - http://bmjopen.bmj.com/site/about/guidelines.xhtml

Nafees B, Stafford M, Gavriel S, Bhalla S, Watkins J. Health state utilities for non small cell lung cancer. Health and Quality of Life Outcomes 2008;6:84.

Omenn GS, Anderson KW, Kronmal RA, Vlietstra RE. The temporal pattern of reduction of mortality risk after smoking cessation. American Journal of Preventive Medicine 1990;6:251-257.

Orme ME, Hogue SL, Kennedy LM, Paine AC, Godfrey C. Development of the health and economic consequences of smoking interactive model. Tobacco Control 2001;10:55-61.

Peto R, Darby S, Deo H, Silcocks P, Whitley E, Doll R. Smoking, smoking cessation, and lung cancer in the UK since 1950: combination of national statistics with two case-control studies. BMJ 2000;321:323-9. (Data supplement).

Secker-Walker RH, Worden JK, Holland RR, Flynn BS, Detsky AS. A mass media programme to prevent smoking among adolescents: costs and cost effectiveness. Tobacco Control 1997;6:207-212.

Sonnenberg FA, Beck JR. Markov models in medical decision making: A practical guide. Medical Decision Making 1993;13:322-38.

Statistics Sweden (SCB). Statistics database. www.statistikdatabasen.scb.se Accessed October 2015.

Sullivan PW, Lawrence WF, Ghushchyan V. A national catalogue of preference-based scores for chronic conditions in the United States. Medical Care 2005;43:736-749.

Surgeon General. The health benefits of smoking cessation. U.S. Department of Health and Human Services, Public Health Service, Centres for Disease Control, Center for Chronic Disease Prevention and Health Promotion, Office on Smoking and Health. DHHS Publication No. (CDC) 90-8416. 1990.

Swedish Association of Local Authorities and Regions (SALAR). KPP- database. Accessed 2015-10-01 at https://stat.skl.se/kpp/index.htm

Swedish National Board of Health and Welfare. Statistics database. Diagnoses in inpatient care from the Hospital Discharge Register. Accessed 2015-10-01 at www.socialstyrelsen.se/statistik/statistikdatabas/diagnoserislutenvard

Swedish National Board of Health and Welfare. The Swedish AMI Statistics. Accessed 2015-10-01 at www.socialstyrelsen.se/statistik/statistikdatabas/hjartinfarkter

Swedish National Board of Health and Welfare. The Swedish Stroke Statistics. Accessed 2015-10-01 at www.socialstyrelsen.se/statistik/statistikdatabas/stroke

Swedish National Board of Health and Welfare. The Swedish Causes of Death Register. Accessed 2015-10-01 at www.socialstyrelsen.se/statistik/statistikdatabas/dodsorsaker

Swedish National Heart Failure Register. Accessed 2015-10-01 at http://www.ucr.uu.se/rikssvikt/

Talbäck M, Rosén M, Stenbeck M, Dickman PW. Cancer patient survival in Sweden at the beginning of the third millennium -Predictions using period analysis. Cancer Causes and Control 2004;15:967-976. Accessed 2015-10-10 at www.socialstyrelsen.se/statistics/cancersurvival

For peer review only - http://bmjopen.bmj.com/site/about/guidelines.xhtml

Tengs TO, Osgood ND, Chen LL. The cost-effectiveness of intensive national school-based anti-tobacco education: Results from the Tobacco Policy Model. Preventive Medicine 2001;33:558-570.

TLV (Swedish Dental and Pharmaceutical Benefits Agency). General guidelines for economic evaluations from the Pharmaceutical Benefits Board (LFNAR 2003:2). Accessed 2016-04-24 at http://tlv.se/Upload/English/Guidelines-for-economic-evaluations-LFNAR-2003-2.pdf

Weinstein MC, Coxson PG, Williams LW, Pass TM, Stason WB, Goldman. L. Forecasting coronary heart disease incidence, mortality and cost: The Coronary Heart Disease Policy Model. American Journal of Public Health 1987;77:1417-1426.

Zethraeus N, Molin T, Henriksson P, Jönsson B. Costs of coronary heart disease and stroke: the case of Sweden. Journal of Internal Medicine 1999;246:151-159.

For peer review only

Consolidated Health Economic Evaluation Reporting Standards (CHEERS) statement

Section/item	Item No	Recommendation	Reported on page No/ line No
**Title and abstract**			
Title	1	Identify the study as an economic evaluation or use more specific terms such as "cost-effectiveness analysis", and describe the interventions compared.	Title, page 1
Abstract	2	Provide a structured summary of objectives, perspective, setting, methods (including study design and inputs), results (including base case and uncertainty analyses), and conclusions.	Abstract, page 3
**Introduction**			
Background and objectives	3	Provide an explicit statement of the broader context for the study. Present the study question and its relevance for health policy or practice decisions	Page 6, lines 1-15
**Methods**			
Target population and subgroups	4	Describe characteristics of the base case population and subgroups analysed, including why they were chosen.	Page 7-8
Setting and location	5	State relevant aspects of the system(s) in which the decision(s) need(s) to be made.	Page 6, lines 22-27<br>Page 7, lines 1-6
Study perspective	6	Describe the perspective of the study and relate this to the costs being evaluated.	Page 9, lines 1-15<br>Page 10, lines 14-24
Comparators	7	Describe the interventions or strategies being compared and state why they were chosen.	Page 8. lines 1112, Page 9, lines 1-15
Time horizon	8	State the time horizon(s) over which costs and consequences are being evaluated and say why appropriate.	Page 10, lines 26-27<br>Page 11. Lines 1-2
Discount rate	9	Report the choice of discount rate(s) used for costs and outcomes and say why appropriate.	Page 9, line 12
Choice of health outcomes	10	Describe what outcomes were used as the measure(s) of benefit in the evaluation and their relevance for the type of analysis performed.	Page 10, lines 5-10
Measurement of effectiveness	11a	*Single study-based estimates:* Describe fully the design features of the single effectiveness study and why the single study was a sufficient source of clinical effectiveness data.	Page 7, lines 18-25<br>Page 8, table 1

For peer review only

Section/item	Item No	Recommendation	Reported on page No/ line No
	11b	*Synthesis-based estimates*: Describe fully the methods used for identification of included studies and synthesis of clinical effectiveness data.	Not applicable
Measurement and valuation of preference based outcomes	12	If applicable, describe the population and methods used to elicit preferences for outcomes.	Not applicable
Estimating resources and costs	13a	*Single study-based economic evaluation:* Describe approaches used to estimate resource use associated with the alternative interventions. Describe primary or secondary research methods for valuing each resource item in terms of its unit cost. Describe any adjustments made to approximate to opportunity costs.	Not applicable
	13b	*Model-based economic evaluation:* Describe approaches and data sources used to estimate resource use associated with model health states. Describe primary or secondary research methods for valuing each resource item in terms of its unit cost. Describe any adjustments made to approximate to opportunity costs.	Page 9, lines 6-15<br>Page 10, lines 12-27<br>Page 11, lines 1-8<br>Appendix 1
Currency, price date, and conversion	14	Report the dates of the estimated resource quantities and unit costs. Describe methods for adjusting estimated unit costs to the year of reported costs if necessary. Describe methods for converting costs into a common currency base and the exchange rate.	Page 9, lines 25-26<br>Page 10, Lines 1-4
Choice of model	15	Describe and give reasons for the specific type of decision-analytical model used. Providing a figure to show model structure is strongly recommended.	Page 5, lines 8-17<br>Page10, lines 14-23<br>Appendix 1
Assumptions	16	Describe all structural or other assumptions underpinning the decision-analytical model.	Appendix 1
Analytical methods	17	Describe all analytical methods supporting the evaluation. This could include methods for dealing with skewed, missing, or censored data; extrapolation methods; methods for pooling data; approaches to validate or make adjustments (such as half cycle corrections) to a model; and methods for handling population heterogeneity and uncertainty.	Page 11, lines 3-8<br>Appendix 1
**Results**			

For peer review only

Section/item	Item No	Recommendation	Reported on page No/ line No
Study parameters	18	Report the values, ranges, references, and, if used, probability distributions for all parameters. Report reasons or sources for distributions used to represent uncertainty where appropriate. Providing a table to show the input values is strongly recommended.	Appendix 1
Incremental costs and outcomes	19	For each intervention, report mean values for the main categories of estimated costs and outcomes of interest, as well as mean differences between the comparator groups. If applicable, report incremental cost-effectiveness ratios.	Page 12-13, Table 2<br>Page 16, Table 3
Characterising uncertainty	20a	*Single study-based economic evaluation:* Describe the effects of sampling uncertainty for the estimated incremental cost and incremental effectiveness parameters, together with the impact of methodological assumptions (such as discount rate, study perspective).	Not applicable
	20b	*Model-based economic evaluation:* Describe the effects on the results of uncertainty for all input parameters, and uncertainty related to the structure of the model and assumptions.	Page 17, lines 17-21<br>Page 18, lines 1-5<br>Page 18, figure 1
Characterising heterogeneity	21	If applicable, report differences in costs, outcomes, or cost-effectiveness that can be explained by variations between subgroups of patients with different baseline characteristics or other observed variability in effects that are not reducible by more information.	Not applicable
**Discussion**			
Study findings, limitations, generalisability, and current knowledge	22	Summarise key study findings and describe how they support the conclusions reached. Discuss limitations and the generalisability of the findings and how the findings fit with current knowledge.	Pages 17-21
**Other**			
Source of funding	23	Describe how the study was funded and the role of the funder in the identification, design, conduct, and reporting of the analysis. Describe other non-monetary sources of support.	Page 21 "Funding"
Conflicts of interest	24	Describe any potential for conflict of interest of study contributors in accordance with journal policy. In the absence of a journal policy, we recommend authors comply with International Committee of Medical Journal Editors recommendations.	Page 21 "Competing interests"

The CHEERS statement checklist format is based on the format of the CONSORT statement checklist

For peer review only

For peer review only

BMJ Open

Cost-effectiveness of a high- vs a low-intensity smoking cessation intervention in a dental setting: long-term follow up

Journal:	*BMJ Open*
Manuscript ID	bmjopen-2019-030934.R1
Article Type:	Research
Date Submitted by the Author:	19-Jun-2019
Complete List of Authors:	Feldman, Inna; Uppsala Universitet, Department of Public Health and Caring Science<br>Helgason, Asgeir; Karolinska Institutet, Department of Public Health Sciences, Social Medicine<br>Johansson, Pia; Public Health & Economics<br>Tegelberg, åke; Centre for Clinical Research, Uppsala University, Hospital of Vastmanland<br>Nohlert, Eva; Centre for Clinical Research, Uppsala University and Region Vastmanland,
**Primary Subject Heading**:	Health economics
Secondary Subject Heading:	Dentistry and oral medicine, Health services research
Keywords:	smoking cessation, cost-effectivenes, long-term sustainability

SCHOLARONE™
Manuscripts

1

Cost-effectiveness of a high- vs a low-intensity smoking cessation intervention in a dental setting: long-term follow up.

Inna Feldman[1], Ásgeir R Helgason[2.3], Pia Johansson[4], Åke Tegelberg[5.6] and Eva Nohlert[5]

[1] - Department of Public Health and Caring Sciences, Uppsala University, Uppsala, Sweden

[2] - Karolinska Institutet, Stockholm, Sweden

[3] - Reykjavik University & Icelandic Cancer Society, Reykjavik, Iceland

[4] - Public Health & Economics, Kyrkängsbacken 8, Huddinge, Sweden

[5] - Centre for Clinical Research, Uppsala University, Hospital of Västmanland, Västeras. Sweden

[6] - Faculty of Odontology, Malmö University, Malmö, Sweden

Corresponding author: Inna Feldman. inna.feldman@pubcare.uu.se

For peer review only

Abstract

Objectives. The aim of this study was to conduct a cost-effectiveness analysis of a high- and a low-intensity smoking cessation treatment programme (HIT and LIT) using long-term follow-up effectiveness data and to validate the cost-effectiveness results based on short-term follow-up.

Design and outcome measures. Intervention effectiveness was estimated in a randomized controlled trial as numbers of abstinent participants after 1 and 5–8 years follow-up. The economic evaluation was performed from a societal perspective using a Markov model by estimating future disease-related costs (in Euro (€) 2018) and health effects (in quality-adjusted life-years, QALYs). Programmes were explicitly compared in an incremental analysis, and the results were presented as an incremental cost-effectiveness ratio (ICER).

Setting. Dental clinics in Sweden.

Participants. 294 smokers aged 19–71 years.

Interventions. Behaviour therapy, coaching and pharmacological advice (HIT) was compared with one counselling session introducing a conventional self-help programme (LIT).

Results. The more costly HIT led to higher number of 6-month continuous abstinent participants after 1 year and higher number of sustained abstinent participants after 5–8 years, which translates into larger societal costs avoided and health gains than LIT. The incremental cost/QALY of HIT compared to LIT amounted to €918 and €3,786 using short- and long-term effectiveness respectively, which is considered very cost-effective in Sweden.

Conclusion. Cost-effectiveness analysis favours the more costly HIT if decision-makers are willing to spend at least €4,000/QALY for tobacco cessation treatment.

For peer review only

Strengths and limitations of this study

- This study utilises a unique possibility to compare cost-effectiveness analyses based on 1-year and 5-8 years follow-up data.
- This economic evaluation clearly supports that more intensive and costly smoking cessation provision is cost-effective.
- The calculation of the intervention costs for the cessation programmes was based on a trial protocol and might be overestimated in comparison with routine practice.
- The effects of smoking cessation are probably underestimated since only three disease groups are modelled and no effects of passive smoking are included.

For peer review only

4

Introduction

Smoking is likely to remain the single most important preventable health risk in the world. Despite continuously declining prevalence in recent decades, one in ten adults in Sweden still smokes daily [1]. Cigarette smoking contributes to 7.5% of the burden of disease in Sweden [2] and was estimated to account for approximately €3,000,000 (31.5 billion Swedish krona, SEK), including €1,000,000 (11 billion SEK) in healthcare costs (15% of the national costs for health and welfare sector) and €1,500,000 (16 billion SEK) in productivity costs in year 2015 [3]. A decrease in prevalence of smoking to five per cent could save society €1,300,000 (14.3 billion SEK) per year.

Several smoking cessation interventions, targeted at current smokers, are available; furthermore, evaluations so far have confirmed the effectiveness of the majority of them. Additionally, some recent studies emphasise that higher level of intervention intensity, such as additional counselling sessions [4] and intensive support through a mobile application [5], resulted in the highest smoking cessation rates. However, due to increasing number of available interventions, decision-makers have to decide which intervention to implement, taking into account that intervention intensity increases intervention costs. Relative costs and benefits of those interventions are important criteria, thus, increasing the attention on economic evaluations in recent years [6 7]. Economic evaluations combine the costs and outcomes of different interventions and aim to determine which intervention provides the best value for money [8]. Several studies on the cost-effectiveness of smoking cessation interventions comparing different intensity of support have been performed during the last few years. For example, Quit-and-Win programme [9], comparison of standard, enhanced and intensive smoking cessation interventions using cell phones [10], and two smoking cessation

For peer review only

5

approaches of different level of intensity for cancer patients [11]. The results suggested that the higher intensive interventions are preferable from health economics point of view, but all those evaluations were based on 6- or 12-months follow-up, long-term follow-ups are scarce in randomised controlled trials.

The effects of smoking on health occur during many years because current smoking influences future health risks; similarly, a smoking cessation today will cause smoking related health risks to tail off gradually. Thus, in order to estimate cost-effectiveness of smoking cessation interventions, a lifetime perspective is necessary, taking into account a variety of different costs and effects [12]. Hence, the well-established method to perform cost-effectiveness analyses of smoking cessation interventions involves mathematical modelling of future events as consequences of smoking. Systematic reviews of model-based economic evaluations in smoking cessation analysed different aspects, such as type of model, quality of the model, transferability, and comparison of the results in different studies [12-14]. Berg at al. [13] identified 64 economic evaluations in smoking cessation, and the state-transition Markov model was most frequently used. The majority of the models simulates the lifetime development of morbidity and mortality for smoker vs former smoker using relative risks for four diseases, chronic obstructive pulmonary disease (COPD), coronary heart disease (CHD), stroke, and lung cancer. The authors concluded that existing economic evaluations in smoking cessation vary in quality, resulting mainly from the way in which they selected their populations, measured costs and effects, and assessed the variability and generalisability of their own findings [13]. One of the reasons of the quality issues is that all those studies are based on short-term follow-up (from six months to one year), and they have no possibilities to validate the sustainability of short-term effectiveness in real life; thus, they cannot confirm the reported cost-effectiveness results and policy recommendations. Moreover, the long-term

For peer review only

assumption, such as relapse rate, might change the results of the smoking cessation cost-effectiveness [15].

Our previous economic evaluation of high- and low-intensity programmes (HIT and LIT) for smoking cessation in a dental setting was based on the reported number of quitters measured as point prevalence abstinent (not one puff of smoke during the past seven days prior to 1-year follow-up). The conclusion was that high-intensity treatment support is the preferred option if the decision-makers' willingness-to-pay exceeds €5,100 (50,000 SEK) per QALY. The base-case scenario of the analysis assumed a sustained abstinence for the quitters [16]. The long-term follow-up of the programmes was performed five to eight years later [17]. In this study, we used a unique opportunity to compare cost-effectiveness analyses of a high- and a low-intensity smoking cessation intervention in a dental setting, using data from short-term (1-year) and long-term (5–8 years) follow-up.

We set out to: 1) perform a cost-effectiveness analysis of a high- and a low-intensity smoking cessation programme in a dental setting using long-term (5–8 years) follow-up data and 2) compare the cost-effectiveness results with the previous study based on short-term (1-year) follow-up.

Methods

Summary of the smoking cessation study

In the smoking cessation intervention study [18], between August 2003 and February 2005, 300 adult smokers recruited via direct inquiry or advertising in dental or general health care were offered smoking cessation support performed in a dental setting. Inclusion criteria were daily smokers over 20 years of age, while exclusion criteria were reading difficulties and problems

For peer review only

with Swedish language. The participants were randomly assigned to two interventions; one received high-intensity and one low-intensity treatment support.

The high-intensity smoking cessation treatment, the HIT programme, comprised eight individual sessions, of in total 3.5 hr over a period of 4 months, and was based on behaviour therapy, coaching and pharmacological advice. The low-intensity smoking cessation treatment, the LIT programme, comprised one counselling session, of up to 45 min, introducing a conventional self-help programme running over 8 weeks. Both programmes were free of charge.

The participants answered a baseline questionnaire and a short-term (one year after the planned smoking cessation date) follow-up questionnaire. Demographic characteristics such as gender, age and education level were also collected. The effectiveness of the trial was reported elsewhere [18]. The analysis concluded that the more extensive and expensive HIT programme was more effective and cost-effective, in terms of proportion of smokers who were still smoke-free after one year [16 18]. The long-term follow-up was performed 5–8 years after the planned smoking cessation date. The effectiveness analysis showed that the difference in outcome between the HIT and LIT programmes remained relatively constant and significant in favour of HIT, and that abstinence at 1-year follow-up was a good predictor for long-term abstinence [17]. All analyses were done using the "intention to treat" approach where non-responders were considered as smokers. Mortality and morbidity data for the participants were not collected either by questionnaire or through the registers. The original study, as well as the long-term follow-up, was approved by the ethical committee at Uppsala University (Dnr:Ups 02–457, Dnr: 2010/172).

The mean age of the participants was 49 years, and 78% were women. Short-term follow-up (one year) questionnaire was answered by 84% of the randomised participants (88% for HIT vs 81% for LIT). Fourteen per cent (41 of the 300 participants) reported 6-month continuous

For peer review only

abstinence (not one puff of smoke during the past 6 month); 27 (18%) individuals in HIT vs 14 (9%) in LIT. At long-term follow-up (5–8 years), 241 persons answered the questionnaire (80% for both HIT and LIT). Of those, 24 were sustained abstinent (17 vs 7 for HIT vs LIT) since the planned smoking cessation date. Relapse rate was 26% and 50% for participants reported 6-month continuous abstinence at 1-year follow-up in HIT and LIT respectively, but the difference was not statistically significant**.** Characteristics of the study participants as well as abstinence at the 1-year and at the long-term follow-up are presented in Table 1.

Table 1. Characteristics of the study participants and programme effectiveness at the 1- and 5-8-year follow-up, by treatment intensity.

	HIT N=150	LIT N=150	p-value
Study participants (number)			
Baseline measures	146	148	
12-month follow-up measures	132	122	
Available at long-term follow-up	141	143	
Long-term follow-up measures	121	120	
Participants characteristics			
Gender (number):			
Men	26	32	
Women	115	111	.410
Age at baseline (age):			
mean (SD)	48.7 (9.6)	48.5 (11.0)	
median	48.0	49.0	.825
Education (in years) (number):			
0 - 9	25	36	
10 - 12	60	55	
>=13	52	50	.336
Number of smoked cigarettes/week at baseline:			
mean (SD)	106 (50)	105 (40)	
median	105	105	.794
Intervention effectiveness (number)			
1-year follow-up:			
6-month continuous abstinence	27	14	.034*
5-8 year follow-up:			

For peer review only

Sustained abstinence	17	7	.030*
Relapse rate (%)	26	50	.345

* statistical significant differences at 0.05 level in effectiveness between the programmes

Economic evaluation

Two economic evaluations were performed to obtain the cost-effectiveness of the more costly HIT programme in comparison to LIT:

1) Cost-effectiveness analyses (CEA) based on the number and characteristics of 6-month continuous abstinent participants according to 1-year follow-up, CEA short-term; and

2) Cost-effectiveness analyses based on the number and characteristics of sustained abstinent participants since planned smoking cessation date according to 5–8 years follow-up, CEA long-term.

Both analyses used the same methodology described below.

Economic evaluations were based on the costs to implement the programmes, the number and characteristics of abstinent participants and on a previously constructed Markov model that estimates the future health and cost consequences of smoking cessation. All costs were inflated to reflect 2018 costs according to the Swedish consumer price index [19] and converted into 2018 Euro (€) using the purchasing power parity (PPP) estimates with CCEMG – EPPI-Centre Cost Converter (http://eppi.ioe.ac.uk/costconversion/default.aspx). The cost-effectiveness analyses followed Swedish and international recommendations: costs were calculated from a societal perspective, health effects expressed as quality-adjusted life-years (QALYs), and programmes explicitly compared in an incremental analysis (incremental cost-effectiveness ratio (ICER), with discounting (3% per year) and sensitivity analyses [8 20]. The ICER was calculated by dividing the difference in total costs for the programmes (incremental

For peer review only

cost) by the difference in the health outcomes in QALYs (incremental effect) to provide a ratio of extra cost per extra unit of health effect.

Intervention costs

The intervention costs were collected prospectively by interviewing the three dental hygienists who carried out the patient work as well as the project leader and the project coordinator. The costs were divided into joint costs for the two programmes and programme-specific costs, and undiscounted because of the short 3-year project time. The joint costs were assumed, divided equally between the programmes while the programme-specific costs included staff time for patient work, material, and participant costs. Estimation of the intervention costs has been described in detail previously [16]. Total programme-specific costs amounted to €117,011; €801 per participant for HIT and €27,927; €189 per participant for LIT.

For peer review only

Intervention effectiveness

For CEA short-term, we used 6-month continuous abstinence at 1-year follow-up reported by 41 participants (14 from HIT and 27 from LIT). For CEA long-term, we used sustained abstinence at 5–8 years reported by 24 participants (17 from HIT and 7 from LIT), see Table 1. Both measures were statistically significant different between the treatment programs. In order to generalize the long-term effectiveness of our study, we performed a logistic regression analysis to calculate the probability of sustained abstinence depending on programme (HIT vs LIT), participant's gender and age, see Table 2.

Table 2. Logistic regression analysis of factors associated with sustained abstinence at 5–8 years follow-up

	Coefficient	p-value	OR#	95% CI##

HIT programme	1.001	0.03*	2.72	1.09-6.80
Mail gender	-0.077	0.88	0.93	0.32-2.64
Age	0.005	0.82	1.00	0.96-1.05
Constant	-3,124	0.01	0.04	

* statistical significant at 0.05 level

$^{\#}$ - Odds Ratio

$^{\#\#}$ - Confidence Interval

The type of the programme (HIT vs LIT) was significantly associated with sustained abstinence while gender and age were not. The regression equation [1] demonstrates dependence between “abstinence” (1 - abstinence, 0 - no abstinence) and “programme” (1 - HIT, 0 - LIT), “gender” (1 - male, 0 - female) and “age” (19-71):

$$\text{abstinence} = -3.124 + 1.001*\text{programme} - 0.077*\text{gender} + 0.005*\text{age} \qquad [1]$$

Equation [1] allows us to calculate the probability of long-term abstinence, $P_{q,}$ for a random participant (a random man/woman from a population of interest, smoker between 19 and 71 years old) in respective programme, see equation [2].

$$P_q = EXP(\text{abstinence}) / (1+EXP(\text{abstinence})) \qquad [2]$$

Markov model

A Markov model was used to estimate health consequences and societal costs of smoking cessation, further described in a technical report [21]. The model has been used in similar studies in Sweden [16 22 23], and the updated year 2015 version was used for the current analysis [21]. The model simulates the societal effects of quitting smoking on three disease groups: lung cancer, chronic obstructive pulmonary disease (COPD) and cardiovascular disease, including

For peer review only

coronary heart disease (CHD) and stroke. Even though there are other smoking-related diseases, these conditions cover most of the health problems associated with smoking [24]. The model incorporates the smoking-related disease risks, time-dependent remaining excess disease risks after quitting, the death risks for the specific and for unrelated diseases, as well as the societal costs of the diseases. All disease risks are annual age- and gender-specific excess incidence risks until death or the age of 95. This lifetime horizon was recommended for modelling of smoking cessation interventions [12] because smoking cessation reduces smoking-related health risks gradually during a long period. Notably, the model does not contain the risk for relapse in smoking among the quitters. The societal costs include costs associated with: medical treatment, community care, drugs, informal care and other expenditures for patients and relatives as well as morbidity productivity costs. Health outcomes are expressed in QALYs. The number of QALYs were calculated during healthy years and years spent with a disease, until death or the age of 95. The model and all the parameters are described in detail in a technical report [21] and Appendix 1.

Model simulation were performed according to gender and 5-year age groups. The simulations result in accumulated societal costs and health effects for life-long continuing smokers and quitters at a specific age and gender group, respectively. The differences in societal costs and health effects between smoking statuses at a certain age are then compared outside the model, and constitute the avoided costs and gained health effects from the tobacco quitting for the specified age and gender group

Sensitivity analyses

Extensive sensitivity analyses on parameter values and methodological choices were reported in the model technical report [21]. The model estimates were, in general, insensitive to changes in parameter values, except the most conservative multivariate analysis where the costs were

For peer review only

decreased by 25%, the disease risks by 50%, the death risks by 10%, and the risk fractions after quitting by 0.1. This low cost/low risk analysis led to substantial decreases in cost and QALY differences between quitters and smokers. This sensitivity analysis was applied to compare costs and effects between HIT and LIT, to validate the results of the CEA long-term. To increase the generalizability of the cost-effectiveness results, we have also applied the probabilities of long-term abstinence depending on programme (HIT vs LIT), participant's gender and age on the modelling results. We estimated the avoided social costs and gained QALYs for a random quitter from our sample and then adjusted the results to the probability to quit (Abstinence), calculated in [1]. Cost-effectiveness was estimated for men and women separately.

Further, a probabilistic sensitivity analysis (PSA) was conducted, based on the uncertainty of the difference in sustained abstinent participants in the two programmes. The effectiveness of LIT was fixed at the 7% quit rate, but the HIT quit rate was sampled from the 95% confidence interval (9% – 22%). The PSA was performed by 10,000 runs, using the societal costs avoided and QALY gains for the group with the largest number of quitters, i.e. women aged 40–44 years. The PSA was presented as a cost-effectiveness acceptability curve, which indicates the probability that HIT is cost-effective versus LIT at different values of the willingness-to-pay for a QALY.

Patient and Public Involvement

This research was done without patient involvement. Patients were not invited to comment on the study design and were not consulted to develop patient relevant outcomes or interpret the results. Patients were not invited to contribute to the writing or editing of this document for readability or accuracy.

For peer review only

Results

Model estimates

Model estimates for the CEA short-term and CEA long-term are presented in Table 3 (societal costs and QALYs). The second and third columns in Table 3 present the estimation of avoided societal costs and QALYs gained for a person with respective gender and age, who became sustained abstinent in comparison with a continuing smoker. Using this data, we can estimate the difference in societal cost avoided and QALYs gained by multiplying difference in numbers of 6-month continuous abstinent participants between the treatment programmes (N*) or difference in numbers of sustained abstinent participants since planned smoking cessation date between the treatment programmes (N**) by societal costs avoided and QALYs gained.

For peer review only

15

Table 3. Model estimates of societal costs avoided and QALYs gained. Costs in **Euro 2018**. 3 % discount rate.

	Model estimates		CEA[a]-short					CEA[a]-long				
			HIT[b]	LIT[c]	Difference			HIT[b]	LIT[c]	Difference		
Gender/ Age group	Costs avoided	QALYs[d] gained	N_{Hp}[e]	N_{Lp}[f]	N*	Costs	QALYs[d]	N_{Hs}[g]	N_{Ls}[h]	N**	Costs	QALYs[d]
Women												
20-24	8142	0.61		1	-1	-8142	-0.61					
25-29	8425	0.65	1		1	8425	0.65					
35-39	9267	0.71	2	2	0			1	1	0		
40-44	8532	0.71	5		5	42658	3.55	4		4	34126	2.84
45-49	6772	0.66	3	3	0			1	2	-1	-6772	-0.66
50-54	5228	0.61	4	3	1	5228	0.61	1	2	-1	-5228	-0.61
55-59	4542	0.43	4	2	2	9085	0.86	4	1	3	13627	1.29
60-64	3336	0. 32	4		4	13342	1.29	2		2	6671	0.64
65-69	2023	0.33		1	-1	-2023	-0.33					
Men												
20-24	10430	0.74	1		1	10430	0.74	1		1	10430	0.74
40-44	10526	1.00	1		1	10526	1.00	1		1	10526	1.00
45-49	11416	0.82	1	1	0			1	1	0		
50-54	11360	0.78		1	-1	-11360	-0.78					
65-69	4084	0.46	1		1	4084	0.46	1		1	4084	0.46
Total			27	14	13	82253	7.44	17	7	10	67466	5.71

For peer review only

[a] Cost-effectiveness analysis
[b] High-intensity smoking cessation treatment, the HIT programme
[c] Low-intensity smoking cessation treatment, the LIT programme
[d] Quality-adjusted life-years
[e] N_{Hp} – number of 6-month continuous abstinent participants HIT treatment programme according to 1-year follow-up
[f] N_{Lp} – number of 6-month continuous abstinent participants LIT treatment programme according to 1-year follow-up
[g] N_{Hs} – number of sustained abstinent participants HIT treatment according to 5-8 year follow-up
[h] N_{Ls} – number of sustained abstinent participants LIT treatment according to 5-8 year follow-up
N*– difference in numbers of 6-month continuous abstinent participants between the treatment programmes according to 1-year follow-up
N**– difference in number of sustained abstinent participants between the treatment programmes according to 5-8 year follow-up

The CEA short-term indicated that HIT led to additional avoided societal costs of €**82,253** and additional 7.44 QALYs compared with LIT. The CEA long-term reported the difference between HIT and LIT as additional avoided societal costs of €**67,466** and additional 5.71 QALYs.

Cost-effectiveness analyses

The more costly HIT programme led to a higher number of 6-month continuous abstinent participants at 1-year follow-up (CEA short-term) as well as higher number of sustained abstinent participants at 5–8 year follow-up (CEA long-term), which translates into larger costs avoided and health gains than LIT, see Table 4. However, the difference in intervention costs were not fully balanced by the societal costs avoided, so HIT implied an incremental net cost of about €6,832 in CEA short-term and €21,619 in CEA long-term, compared with LIT. HIT was estimated to lead to more QALYs, so the incremental cost per QALY of HIT compared with LIT amounted to €918 for CEA short-term and €3,786 for CEA long-term, which is considered to be very cost-effective in Sweden [20]. The incremental analysis favours the more costly HIT, if decision-makers are willing to spend at least €4,000/QALY for tobacco cessation programmes.

Table 4. Incremental cost-effectiveness analyses, CEA, of the two smoking cessation treatments, HIT and LIT, for 6-month continuous abstinence at 1-year (CEA short-term), sustained abstinence at 5–8 year follow-up (CEA long-term), and sensitivity analyses for CEA long-term. Societal perspective, in Euro 2018.

				CEA[a] -long, population level, per person	
Intervention costs	CEA[a]-short	CEA[a] -long	CEA[a] -long, sensitivity	Men	Women
HIT[b]	117011	117011	117011	801	801
LIT[c]	27927	27927	27927	189	189
Difference in intervention costs	89085	89085	89085	612	612
Difference in societal costs avoided	82253	67466	32 469	779	502
Incremental costs	6832	21619	56616	-167	110
Incremental QALYs[d]	7,44	5,71	4,82	0,0664	0,0462
Incremental cost per QALY[d] (ICER*)	918	3786	11746	<0	2391

* Incremental cost-effectiveness ratio (ICER) is calculated as incremental costs divided by incremental QALYs

[a] – Cost-effectiveness analysis
[b] – High-intensity smoking cessation treatment, the HIT programme
[c] – Low-intensity smoking cessation treatment, the LIT programme
[d] – Quality-adjusted life-years

Sensitivity analyses

The most conservative sensitivity analysis, a multivariate low cost/low risk analysis, was applied to CEA long-term. This analysis led to substantial decreases in avoided social costs and QALY gains for both HIT and LIT. At the same time, the incremental costs increased and incremental QALYs slightly decreased which resulted in higher incremental cost of €**11,746** per QALY see Table 4.

The probability of sustained abstinence varies between 0.11 and 0.13 for men and between 0.12 and 0.14 for women in HIT in different ages. The corresponding numbers are 0.4-0.5 for men and 0.5-0.6 for women in LIT. The model estimates for random man and woman were 9,740/0.83 and €7,165 /0.66 for avoided societal costs/QALYs gained. Given the probability

For peer review only

18

of abstinence, the difference in avoided societal costs per person between HIT and LIT was estimated as €779 for men and €502 for women and the correspondent difference in QALYs gained was 0.0664 for men and 0.0462 for women. The incremental cost-effectiveness ration (ICER) was negative for men (HIT was cost saving and entailed positive health outcomes in comparison to LIT) but amounted to €2,391 for women, which is close to our base-case analysis, see Table 4.

At all values of willingness-to-pay for a QALY, including zero, the HIT was more cost-effective than the LIT, see the probabilistic sensitivity analysis on the HIT quit rate in Figure 1.

(insert figure 1 here)

Figure **1.** Probabilistic sensitivity analysis on the effectiveness (proportion of quitters) of high-intensity treatment (HIT) in comparison with low-intensity treatment (LIT), reported as cost-effectiveness acceptability curve, willingness-to-pay per quality-adjusted life-year (QALY), in Euro 2018.

Discussion

Main results

In this study, we performed a cost-effectiveness analysis using the long-term follow-up data from a RCT of a high- and a low-intensity treatment programme (HIT and LIT) for smoking cessation in a dental setting. We also validated the cost-effectiveness results of the previous study based on short-term follow-up [16]. HIT was more effective in getting participants to quit smoking and to keep sustained abstinent, resulted in higher societal costs avoided and more QALYs gained among both men and women, compared with LIT and thus can be considered cost-effective. The incremental cost-effectiveness ratios (ICERs) were €918 and €3,786 using short- and long-term effectiveness, respectively, which are below the Swedish willingness-to-pay threshold of €50,000 per QALY [25], thus, indicating that the resource intensive HIT was cost-effective compared to the less resource demanding LIT. The results also confirm the

For peer review only

conclusions of the previous cost-effectiveness analyses based on short-term follow-up data, and we would recommend the use of the HIT programme as a cost-effective option for smoking cessation.

Notably, the usage of both the HIT and LIT programmes is not limited to dental settings and can be implemented in other healthcare sectors and delivered by trained nurses instead of dental hygienists. Since the salaries of registered nurses and dental hygienists are comparable, the conclusion of high cost-effectiveness of the HIT programme remains.

However, although HIT was shown to be cost-effective in comparison with LIT, the sensitivity analysis using the probability of abstinence suggested that HIT dominated over LIT for men (saved societal costs and generated more QALYs). In our sample the majority of study participants were women, that is why the results of the sensitivity analysis for women was very close to our base-case analysis.

Strength and limitations

The majority of cost-effectiveness analyses on smoking cessation use one year quit rates in their models; however, it is not uncommon that 6-month quit rates are used [12 26]. The question of how much we can trust the overall conclusions of such analyses always remains, because we do not know for sure what happens subsequently. To our knowledge, this is the first study that utilises a unique possibility to compare a previously conducted cost-effectiveness analyses based on 6-month continuous abstinent participants at 1-year follow-up with a new evaluation, based on sustained abstinence since the planned smoking cessation date up to 5–8 years. We had the possibility to compare the results based on 6-month continuous abstinence (when some time-dependent excess disease risks remained for the first years after quitting) and sustained abstinence for 5–8 years (when the smoking-related excess disease risks had

For peer review only

been reduced). A higher proportion of sustained abstinent participants in HIT compared to LIT contributed to a low ICER for the long-term cost-effectiveness analyses.

The effects of smoking cessation are certainly underestimated in the model estimates since only three disease groups including lung cancer are modelled and no effects of passive smoking are included, but smoking is causally related to at least 15 other types of cancer [33]. In addition, quitting smoking reduced the rate of incidence diabetes to that of non-smokers after five years in women and after 10 years in men [27]. The model does not include the health problems related to passive smoking, such as risk of CHDs in offspring [28] and increase in risk for breast cancer [29]. That makes our estimations more conservative with respect to cost savings and QALYs, although these three diseases do account for over 80% of morbidity (and mortality) associated with smoking and are frequently used in similar studies [15 30]. Another limitation is that the model does not include the relapse rate among the quitters. This tends to overestimate the health and cost consequences of the tobacco quitting based on short-term outcomes, because the relapse rate is presumably higher among the short-term quitters. On the other hand, the relapse rate might be negligibly low among individuals that quit smoking 5-8 years ago and thus not important for the modelling results. Additionally, as mentioned in our previous study [16], the Markov model indicates considerably lower smoking-related disease risks for women reported by large epidemiological studies (see model technical report for details) [21], and thus lower cost savings and health gains from tobacco cessation for women than for men. Finally, the intervention costs for the RCT study calculation was based on the trial protocol and might be overestimated in comparison with routine practice; however, in the ICER, those extra costs were divided equally between the programmes, and thus disregarded.

Comparison with other studies

For peer review only

We could not find any cost-effectiveness analyses based on more than 1-year follow-up, and therefore we compared our results with other studies estimating cost-effectiveness of interventions with different level of intensity using 6- or 12-month follow-up. Thus, a cost-effectiveness analysis of high intensity multiple contests and low intensity enhanced contest of a Quit-and-Win programme reported that high intensity Quit-and-Win leads to an average gain of 0.03 QALYs and was cost-saving, in comparison with lower intensity [9]. Another study presented a cost-effectiveness analysis of three smoking cessation interventions with different intensity levels: Standard Care (SC) (brief advice to quit, nicotine replacement therapy and self-help written materials), Enhanced Care (EC) (SC plus cell phone-delivered messaging) and Intensive Care (IC) (EC plus cell phone-delivered counselling) [10]. The overall conclusion was that the higher intensive intervention (IC) was the most cost-effective strategy both for men and women, which is in line with our results. Additionally, a cost-effectiveness analysis of two smoking cessation approaches for cancer patients was presented in a study from Canada [11]. The basic programme consisted of screening for tobacco use, advice and referral, whereas the best practice programme included a basic programme and pharmacological therapy, counselling and follow-up. The incremental cost-effectiveness ratio of the best practice programme compared to the basic programme was $3,367 per QALY gained for men, and $2,050 per QALY gained for women. These results are very similar to our findings. In our previous study [16], based on the same RCT and 1-year follow-up, a higher ICER of €9,900/QALY and €5,500 /QALY was calculated for point prevalence and continuous abstinence respectively, but the overall conclusion confirmed the cost-effectiveness of HIT at a willingness-to-pay of €10,000.

Conclusions

In conclusion, the more costly HIT smoking cessation programme is an economically attractive option when compared to the LIT programme over a broad range of assumptions.

For peer review only

Cost-effectiveness analysis favours the more costly HIT if decision-makers are willing to spend at least €4,000/QALY for tobacco cessation treatment. These findings can support and guide implementation of smoking cessation programmes.

Contributors

IF and EN conceived and designed the study and drafted the manuscript. Modelling and economic evaluation was carried out by IF and PJ. AR, ÅT and EN were responsible for clinical evaluation of the smoking cessation study. All the authors (IF, AR, ÅT, PJ and EN) contributed to the writing process and have approved the final manuscript.

Funding

This study was funded by grants from the County Council of Västmanland, Sweden (LTV 3999) and Swedish Research Council for Health, Working Life and Welfare (FORTE), grant number 2014-1399.

Competing interests

None declared.

Ethics approval

The Ethical Committee Uppsala University gave clearance for the smoking cessation study Dnr: Ups 02-457.

For peer review only

Data sharing statement

Data is available from corresponding author (IF) on reasonable request.

References

1. [Folkhälsomyndigheten] PHAoS. Public Health Agency of Sweden [Folkhälsomyndigheten] Stockholm2018 [Available from: https://www.folkhalsomyndigheten.se/the-public-health-agency-of-sweden/living-conditions-and-lifestyle/alcohol-narcotics-doping-tobacco-and-gambling/tobacco/ accessed 2018-05-05 2018.
2. Institute for Health Metrics and Evaluation. Country Profiles: Sweden [Available from: http://www.healthdata.org/sweden accessed Nov 5 2015.
3. Andersson E, Toresson Grip E, Norrlid H, et al. Samhällskostnaden för rökningsrelaterad sjuklighet i Sverige [The social cost of smoking-related illness in Sweden]. In: IHE, ed. IHE Rapport 2017:4. Lund, Sweden, 2017.
4. Thomas JL, Luo X, Bengtson J, et al. Enhancing Quit & Win contests to improve cessation among college smokers: a randomized clinical trial. *Addiction* 2016;111(2):331-9. doi: 10.1111/add.13144 [published Online First: 2016/01/16]
5. Vidrine DJ, Fletcher FE, Danysh HE, et al. A randomized controlled trial to assess the efficacy of an interactive mobile messaging intervention for underserved smokers: Project ACTION. *BMC public health* 2012;12:696. doi: 10.1186/1471-2458-12-696 [published Online First: 2012/08/28]
6. McFarland A. Economic evaluation of interventions in health care. *Nursing standard* 2014;29(10):49-58. doi: 10.7748/ns.29.10.49.e9315
7. Anderson R. Systematic reviews of economic evaluations: utility or futility? *Health economics* 2010;19(3):350-64. doi: 10.1002/hec.1486
8. Drummond M, Sculpher M, Torrance G, et al. Methods for the economic evaluation of health care programmes. 3rd edn ed. Oxford: Oxford University Press 2005.
9. Popp J, Nyman JA, Luo X, et al. Cost-effectiveness of enhancing a Quit-and-Win smoking cessation program for college students. *The European journal of health economics : HEPAC : health economics in prevention and care* 2018 doi: 10.1007/s10198-018-0977-z [published Online First: 2018/04/25]
10. Daly AT, Deshmukh AA, Vidrine DJ, et al. Cost-effectiveness analysis of smoking cessation interventions using cell phones in a low-income population. *Tobacco control* 2018 doi: 10.1136/tobaccocontrol-2017-054229 [published Online First: 2018/06/11]
11. Djalalov S, Masucci L, Isaranuwatchai W, et al. Economic evaluation of smoking cessation in Ontario's regional cancer programs. *Cancer medicine* 2018 doi: 10.1002/cam4.1495 [published Online First: 2018/07/19]
12. Bolin K. Economic evaluation of smoking-cessation therapies: a critical and systematic review of simulation models. *PharmacoEconomics* 2012;30(7):551-64. doi: 10.2165/11590120-000000000-00000
13. Berg ML, Cheung KL, Hiligsmann M, et al. Model-based economic evaluations in smoking cessation and their transferability to new contexts: a systematic review. *Addiction* 2017;112(6):946-67. doi: 10.1111/add.13748
14. Kirsch F. A systematic review of quality and cost-effectiveness derived from Markov models evaluating smoking cessation interventions in patients with chronic obstructive pulmonary disease. *Expert review of pharmacoeconomics & outcomes research* 2015;15(2):301-16. doi: 10.1586/14737167.2015.1001976 [published Online First: 2015/01/21]

For peer review only

24

15. Pennington B, Filby A, Owen L, et al. Smoking Cessation: A Comparison of Two Model Structures. *PharmacoEconomics* 2018 doi: 10.1007/s40273-018-0657-y [published Online First: 2018/05/08]
16. Nohlert E, Helgason AR, Tillgren P, et al. Comparison of the cost-effectiveness of a high- and a low-intensity smoking cessation intervention in Sweden: a randomized trial. *Nicotine & tobacco research : official journal of the Society for Research on Nicotine and Tobacco* 2013;15(9):1519-27. doi: 10.1093/ntr/ntt009
17. Nohlert E, Ohrvik J, Tegelberg A, et al. Long-term follow-up of a high- and a low-intensity smoking cessation intervention in a dental setting--a randomized trial. *BMC public health* 2013;13:592. doi: 10.1186/1471-2458-13-592 [published Online First: 2013/06/20]
18. Nohlert E, Tegelberg A, Tillgren P, et al. Comparison of a high and a low intensity smoking cessation intervention in a dentistry setting in Sweden: a randomized trial. *BMC public health* 2009;9:121. doi: 10.1186/1471-2458-9-121 [published Online First: 2009/05/02]
19. Statistics Sweden. Konsumentprisindex [Available from: http://www.scb.se/pr0101/ accessed April 14, 2016.
20. Pharmaceutical Benefits Board. General guidelines for economic evaluations from the Pharmaceutical Benefits Board, 2003.
21. Johansson. A model for economic evaluations of smoking cessation interventions – technical report. Model version 3. Folkhälsa & Ekonomi (Public Health & Economics), 2015.
22. Johansson PM, Tillgren PE, Guldbrandsson KA, et al. A model for cost-effectiveness analyses of smoking cessation interventions applied to a Quit-and-Win contest for mothers of small children. *Scandinavian journal of public health* 2005;33(5):343-52.
23. Virtanen SE, Galanti MR, Johansson PM, et al. Economic evaluation of a brief counselling for smoking cessation in dentistry: a case study comparing two health economic models. *BMJ open* 2017;7(7):e016375. doi: 10.1136/bmjopen-2017-016375 [published Online First: 2017/07/22]
24. Gunning-Schepers LJ . The health benefits of prevention: a simulation approach. *Health policy and planning* 1989(12):1-256.
25. Svensson M, Nilsson FO, Arnberg K. Reimbursement Decisions for Pharmaceuticals in Sweden: The Impact of Disease Severity and Cost Effectiveness. *PharmacoEconomics* 2015;33(11):1229-36. doi: 10.1007/s40273-015-0307-6 [published Online First: 2015/06/22]
26. Chen YF, Madan J, Welton N, et al. Effectiveness and cost-effectiveness of computer and other electronic aids for smoking cessation: a systematic review and network meta-analysis. *Health technology assessment* 2012;16(38):1-205, iii-v. doi: 10.3310/hta16380 [published Online First: 2012/10/11]
27. Will JC, Galuska DA, Ford ES, et al. Cigarette smoking and diabetes mellitus: evidence of a positive association from a large prospective cohort study. *International journal of epidemiology* 2001;30(3):540-6. doi: 10.1093/ije/30.3.540 [published Online First: 2001/06/21]
28. Zhao L, Chen L, Yang T, et al. Parental smoking and the risk of congenital heart defects in offspring: An updated meta-analysis of observational studies. *European journal of preventive cardiology* 2019:2047487319831367. doi: 10.1177/2047487319831367 [published Online First: 2019/03/25]
29. Macacu A, Autier P, Boniol M, et al. Active and passive smoking and risk of breast cancer: a meta-analysis. *Breast cancer research and treatment* 2015;154(2):213-24. doi: 10.1007/s10549-015-3628-4 [published Online First: 2015/11/08]
30. Hurley SF, Matthews JP. The Quit Benefits Model: a Markov model for assessing the health benefits and health care cost savings of quitting smoking. *Cost effectiveness and resource allocation : C/E* 2007;5:2. doi: 10.1186/1478-7547-5-2

For peer review only

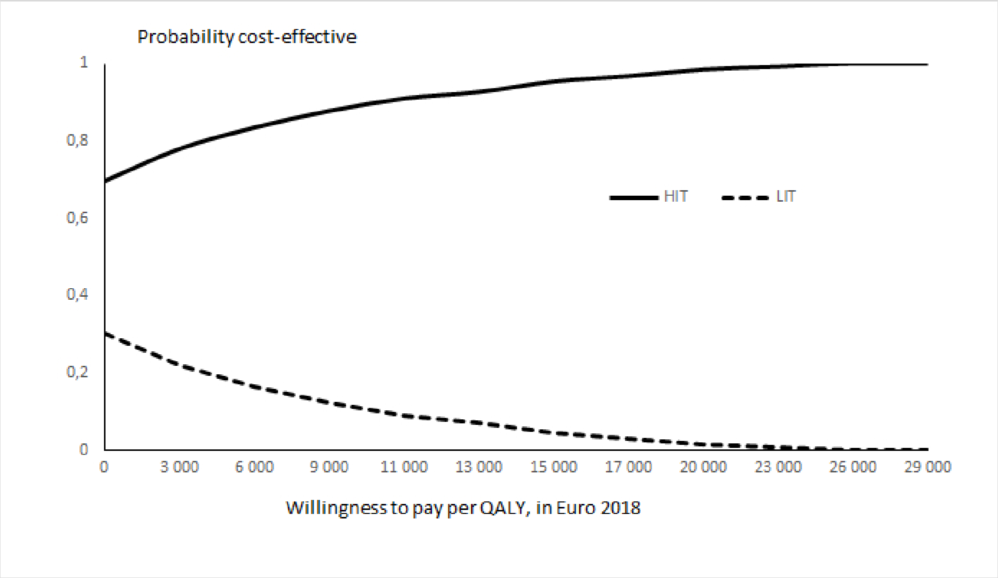

Figure 1. Probabilistic sensitivity analysis on the effectiveness (proportion of quitters) of high-intensity treatment (HIT) in comparison with low-intensity treatment (LIT), reported as cost-effectiveness acceptability curve, willingness-to-pay per quality-adjusted life-year (QALY), in Euro 2018.

A model for economic evaluations of smoking cessation interventions – technical report

Version 3 year 2015

Pia Johansson

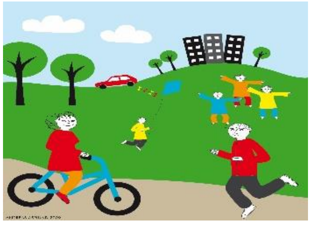

Folkhälsa&Ekonomi
PublicHealth&Economics

Stockholm 2015

Pia.johansson@folkhälsaetekonomi.se
Pia.johansson@publichealtheteconomics.se

This report is available at PublicHealth&Economics.se:
http://www.publichealtheteconomics.se

For peer review only

Content

TABLES **3**

INTRODUCTION **4**

METHOD **5**

THE DISEASES 5
THE MODEL 5

MATERIAL **7**

THE RISKS 7
Disease risks *7*
Death risks *10*
Changes in risk after quitting smoking *12*
THE SOCIETAL COSTS 12
Medical treatment costs *13*
Institutional care and technical aids costs *13*
Pharmaceutical costs *14*
Informal care and other patient and relatives' costs *14*
Productivity costs *15*
THE HEALTH EFFECTS 15
Life years lost *15*
QALYs *15*
SENSITIVITY ANALYSES 16
Univariate analyses *17*
Multivariate analyses *17*
Analyses on methodological issues *17*
Probabilistic analysis *17*

RESULTS **18**

THE MODEL ESTIMATES 18
SELECTED MODEL OUTCOMES 20
SENSITIVITY ANALYSES 22

DISCUSSION: MODEL VALIDITY **25**

THE STRUCTURE OF THE MODEL 25
THE INPUTS OF THE MODEL 26
THE RESULTS OF THE MODEL 28
THE VALUE OF THE MODEL TO THE DECISION-MAKER 29
THE UNCERTAINTY 30
CHECKING FOR TECHNICAL ERRORS 30
CONCLUSIONS 31

REFERENCES **32**

For peer review only

Tables

Table 1. The model diseases, with ICD-10 codes. ... 5
Figure 1. State-transition diagram ... 6
Table 2. Risks COPD. ... 7
Table 3. Risks lung cancer. ... 8
Table 4. Risks CHD and stroke. ... 8
Table 5. The annual risks of CHD. ... 9
Table 6. The annual risks of stroke. ... 9
Table 7. Distribution of diseases within CHD. ... 9
Table 8. Death risk lung cancer. ... 10
Table 9. Death risk AMI, 1st year. ... 10
Table 10. Death risk stroke, 1st year. ... 11
Table 11. Death risk CHF. ... 11
Table 12. Death risks, unrelated. ... 11
Table 13. Medical treatment costs. SEK 2014. ... 12
Table 14. Costs for institutional care and technical aids. SEK 2014. ... 13
Table 15. Pharmaceutical costs. SEK 2014. ... 14
Table 16. Informal care and other patient and relatives' costs. SEK 2014. ... 14
Table 17. Productivity costs, morbidity. SEK 2014. ... 15
Table 18. Average Swedish population QoL weights. ... 16
Table 19. QoL weights and QoL decrements due to disease. ... 16
Table 20. QALYs, until age 95 years, discounted 3%. ... 18
Table 21. Life years lost (YLS), before age 95 years. Discounted 3%. ... 19
Table 22. Life years lost (YLS), before age 95 years. Undiscounted. ... 19
Table 23. Societal costs. In SEK 2014 and discounted 3%. ... 20
Figure 2. The disease outcome, number of diseased and dead per 10 000 for smokers and quitters, women aged 50 years. ... 20
Figure 3. The disease outcome, number of diseased and dead per 10 000 for smokers and quitters, men aged 50 years. ... 21
Table 24. Societal cost savings, in SEK 2014. Women aged 50 years. ... 21
Table 25. Societal cost savings, in SEK 2014. Men aged 50 years. ... 22
Figure 4. Sensitivity analyses on societal cost and QALY differences between smokers and quitters, women aged 50 years. ... 22
Figure 5. Sensitivity analyses on societal cost and QALY differences between smokers and quitters, men aged 50 years. ... 23
Figure 6. The cost-effectiveness plane with resultat från bootstrap, women aged 50 years. ... 23
Figure 7. The cost-effectiveness plane with resultat from bootstrap, men aged 50 years. 24

For peer review only

For peer review only - http://bmjopen.bmj.com/site/about/guidelines.xhtml

Introduction

This is a technical report on an updated version of a model, originally developed in year 2004 (Johansson, 2004), to enable systematic cost-effectiveness analyses of tobacco cessation interventions in Sweden. It aims to follow international and Swedish recommendations of cost-effectiveness analyses in health and medicine. The model holds a societal perspective, aiming to incorporate available disease-specific costs for all sectors of Swedish society. The updated model contains more recent data on societal costs, disease and death risks, and quality-of life-estimates, to enable estimates that reflects current Swedish conditions.

The model simulates the lifetime societal effects of quitting smoking on three diseases: lung cancer, chronic obstructive pulmonary disease (COPD), and cardiovascular disease (CVD) including coronary heart disease (CHD) and stroke. The model incorporates the smoking-related disease risks, the remaining disease risks after tobacco quitting, the death risks in the diseases and unrelated diseases, as well as the societal effects of the diseases. The societal effects include medical treatment costs, costs for institutional care, drug costs, costs for informal care and other costs for patients and relatives, and morbidity productivity costs, as well as loss of life-years and quality-adjusted life-years (QALYs).

This technical report contains a description of the model structure, of all the data sources used and of the assumptions made. For validation purposes, it also reports model estimates for some selected age-groups and more detailed outcomes and sensitivity analyses for one age-group, men and women aged 50 years at the start of the simulations. To investigate model uncertainty, univariate and multivariate sensitivity analyses are reported, as well as a probabilistic analysis. The model validity is discussed in the final section of the report.

For peer review only

For peer review only - http://bmjopen.bmj.com/site/about/guidelines.xhtml

Method

The diseases

The model incorporates the three most smoking-related diseases: lung cancer, chronic obstructive pulmonary disease (COPD), and cardiovascular disease (CVD) including coronary heart disease (CHD) and stroke, see table 1. The model is restricted to the effects on the individual smoker/quitter, thus not incorporating any side-effects on other people.

The model

The stochastic model simulates the societal effects of smoking cessation on three smoking-related diseases. It is constructed as a Markov-cycle tree model appropriate for microsimulations.

The Markov model is a health state-transition model (Sonnenberg & Beck, 1993; Briggs & Sculpher, 1998) using probabilities for transitions between health states. These probabilities are the age- and gender-specific disease risks, conditional on smoking status and years since quitting, and age-, gender- and disease-specific death risks. The states are mutually exclusive and collectively exhaustive, and transitions between disease states are not allowed. The only exits from disease states are death, in the disease in question or in unrelated diseases, except for 5-year survivors in lung cancer which are assumed to recover to complete health. All other disease states are assumed to last life-long. See figure 1 for the state-transition diagram.

The Markov stages are one year-long, with no half-cycle correction. The starting point is the state healthy. The model covers the ages 15 to 95 years. The Markov-cycle tree has been created in Treeage Pro (Treeage Inc., 2015).

For peer review only

Table 1. The model diseases, with ICD-10 codes.

Disease	ICD-10
Lung cancer	C34
COPD	J44
Stroke	I61 I63 I64
Coronary heart disease, CHD:	
Acute myocardial infarction, AMI	I21 I22 I23
Congestive health failure, CHF	I50.
Ischemic heart disease, IHD	I20 I24 I25
Sudden death	I46.1

For peer review only - http://bmjopen.bmj.com/site/about/guidelines.xhtml

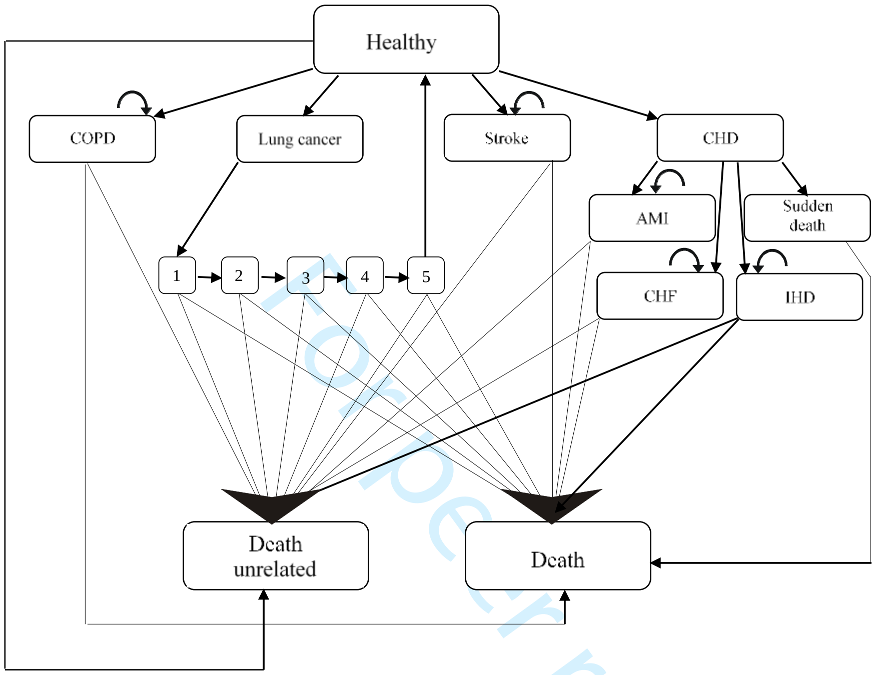

Figure 1. State-transition diagram

The model is set up with two reward sets; costs and effects. The incremental rewards are accumulated during time spent in the health states. The transitional rewards lost life years and some costs are recorded at transitions between healthy and disease state, and disease state and death.

The Markov-cycle tree is run as a microsimulation with 10 000 repetitions. The simulation ends at death or age 95 years. The model is run separately for age and gender groups. The result of each simulation is expected value, with accompanying distributions. The two simulations, the continuing smoker and the quitter, are compared outside the model. The results are presented as expected value per individual, specific for gender, age and smoking status.

For peer review only - http://bmjopen.bmj.com/site/about/guidelines.xhtml

Material

The model is based on principles for cost-effectiveness analysis in health and medicine (Gold et al, 1996; Drummond et al, 2005) and Swedish methodological recommendations (TLV, 2004). The model holds the societal perspective, aiming to incorporate disease-specific costs for all sectors of Swedish society.

The model uses Swedish register data and secondary data from previously published scientific articles. The secondary data was found through searches in the database MEDLINE and the reference lists of retrieved articles, choosing the data that is considered most relevant to present-day Swedish circumstances and the target group. No meta-analysis nor other synthesis of data was performed.

All costs are expressed in year 2014 SEK (USD 1=SEK 6.86; Euro 1=SEK 9.10), converted if necessary by the Swedish CPI (consumer price index). The annual discount rate is 3% for both costs and health effects.

The risks

Disease risks

All disease risks are annual age- and gender-specific excess incidence risk until the age of 95 years, see tables 2 to 5.

The COPD disease risk is taken from the Swedish population-based study Obstructive Lung Disease in Northern Sweden (OLIN), which was started in year 1985 (Lundbäck et al, 1991). The risk is the reported average excess seven-year incidence among smokers in three age groups, of which the youngest was 45 years at baseline, see table 2. COPD was defined according to the spirometer GOLD definition.

Table 2. Risks COPD.

	men & women	source
Disease risk		
Risk until age 45	0%	Lindberg et al, 2006
Excess annual risk for smokers, from age 46	1.6%	
Effect of quitting		
Risk fraction for quitters, years since quitting:		Inspired by Surgeon General, 1990
0-5	1	
6-15	0.5	
16-24	0.3	
>25	0.1	
Death risk		
Excess risk among diseased, as fraction of age-specific general death risk, by age:		Estimated from Lundbäck et al, 2009<br>Statistics Sweden, database
<58 years	1	
58-70 years	5	
>70 years	1	

For peer review only - http://bmjopen.bmj.com/site/about/guidelines.xhtml

Table 3. Risks lung cancer.

	men	women	source
Death risk			
Accumulated death risk until age 75			
Smokers	16.7%	10.4%	Peto et al, 2000
Non-smokers	0.4%	0.4%	
Risk for ages <40	0	0	Assumed, based on Peto et al, 2000
Smokers accumulated excess death risk until age 95	37.2%	23.1%	Interpolated, based on Peto et al, 2000
Age-adjusted conditional death risk	see table 8		
Disease risk			
Smokers accumulated excess disease risk until age 95	42.0%	26.3%	After interpolation, based on Peto et al, 2000 and Holm et al, 1995
Effect of quitting			
Risk fraction for quitters, years since quitting:			Peto et al, 2000
<10	0.66	0.69	
10-19	0.42	0.21	
20-29	0.18	0.05	
30-35	0.08	0	
>36	0	0	

The lung cancer disease risk is estimated from reports on lung cancer deaths until age 75 for smokers (15-24 cigarettes/day) and non-smokers, see table 3. The annual excess death risk is estimated by a quadratic function of the accumulated risk until age 75 years. The lung cancer death risk is assumed 0 until the age of 40 years, and assumed constant between ages 75 and 95. The disease risk is obtained by adjusting the annual death risk by the annual crude survival rate of lung cancer in Sweden for a similar time period as the Peto data, from Holm et al (1995).

Table 4. Risks CHD and stroke.

	men & women	source
Disease risk	Framingham, see tables 5-7	
Effect of quitting		
Risk fraction for quitters, years since quitting:		Surgeon General, 1990
on CHD:		
1	0.5	
>15	0	
on stroke:		
>10	0	
Death risk		
AMI, 1st year	see table 9	
Stroke, 1st year	see table 10	
CHF	see table 11	
Risks as fraction of age- and gender-specific general death risk:		Statistics Sweden
AMI, 2nd and following years, age 15-93 years	3	Henriksson et al, 2014
AMI, 2nd and following years, age >93 years	2	Assumed
Stroke, 2nd and following years, age 15-93 years	3	Henriksson et al, 2014
Stroke, 2nd and following years, age >93 years	2	Assumed
IHD, 1st year	2.5	Granström et al, 2012
IHD, 2nd and following years	2.15	Granström et al, 2012

For peer review only - http://bmjopen.bmj.com/site/about/guidelines.xhtml

For peer review only

Table 5. The annual risks of CHD.

μ_{chd} =5.5305+28.4441*Sex-1.479*Ln(Age)-14.4588*Ln(Age)*Sex+1.8515*(Ln(Age))²*Sex-0.9119*Ln(SBP)-0.2767*Smok-0.7181*Ln(Chol/HDL)-0.1759*Diabetes-0.1999*Diabetes*Sex

P_{chd} =1-Exp(-Exp((- μ_{chd})/ Exp(0.9145-0.2784* μ_{chd})))

Source: Caro et al, 2007; Anderson et al, 1991

Table 6. The annual risks of stroke.

μ_{str} =26.5116+0.2019*Sex-2.3741*Ln(Age)-2.4643*Ln(SBP)-0.3914*Smok-0.0229*Ln(Chol/HDL)-0.3087*Diabetes-0.2627*Diabetes*Sex

P_{str} = 1-Exp(-Exp((- μ_{str})/ Exp(-0.04312* μ_{str})))

Source: Caro et al, 2007; Anderson et al, 1991

The CHD and stroke disease risk estimates are based on the Framingham CVD risk function, see table 4 and tables 5-6. As the Framingham CHD risk function only calculates CHD events, the division of these events into the particular diseases are based on recent Swedish register data, see table 7. To avoid over-estimation of risks, the risk factors for CHD and stroke are evaluated at minimal-risk levels; 120 mmHg for systolic blood pressure (SBP), HDL-cholesterol (HDL) at 1.5 and cholesterol (Chol) at 4. Diabetes is set at 0, while the variable smoking (smok) is set at 1 for the smokers.

Table 7. Distribution of diseases within CHD.

	Age < 65 years		Age >65 years	
	men	women	men	women
AMI	0.42	0.40	0.31	0.31
IHD	0.40	0.39	0.21	0.29
CHF	0.16	0.19	0.46	0.38
Sudden death	0.02	0.02	0.02	0.02

Source: Swedish National Board of Health and Welfare, Statistics database, Diagnoses in inpatient care from the Hospital Discharge Register, year 2013.

For peer review only - http://bmjopen.bmj.com/site/about/guidelines.xhtml

Table 8. Death risk lung cancer.

Age group	Years since diagnosis 1	2	3	4	5
0-54	0.550	0.172	0.034	0.034	0.034
55-74	0.610	0.168	0.030	0.030	0.030
75-95	0.743	0.120	0.021	0.021	0.021

Source Based on Talbäck et al, 2004

Death risks

All death risks are age-and gender disease-specific conditional risks; in some cases estimated as fractions of the general population age- and gender-specific mortality risk, see tables 2 to 4, and in some cases based on Swedish register data, see tables 8 to 11.

The COPD death risk is estimated from the study Obstructive Lung Disease in Northern Sweden (OLIN), which reported the 20-year mortality in three age groups. Comparison with the general age-specific mortality risks revealed no excess risk of death among those younger than 58 years and older than 70 years, but a considerable increased risk among those aged 58-70 years at follow-up. The excess risk was estimated at on average around 5 times the age- and gender-specific general population death risk, see table 2.

The lung cancer death risk is based on survival data from the Swedish National Cancer Registry, see table 8. The death risks for year 3 and 4 after diagnosis are estimated by linear interpolation between years 2 to 5. Lung cancer survivors at 5 years are assumed recovered, and returned to the health state healthy.

The death risks from CHD and stroke are taken from Swedish registers, see tables 9 to 11, or published scientific reports, see table 5. The death risks for AMI, stroke and IHD are divided into risks the first year after the first event and the second and following years after first event.

Table 9. Death risk AMI, 1st year.

Age group	men	women
20-49	0.077	0.077
50-64	0.137	0.101
65-69	0.159	0.149
70-74	0.172	0.141
75-79	0.206	0.191
80-84	0.255	0.224
>84	0.327	0.331

Source: Swedish National Board of Health and Welfare, The Swedish AMI Statistics, year 2013

For peer review only - http://bmjopen.bmj.com/site/about/guidelines.xhtml

Table 10. Death risk stroke, 1st year.

Age group	men	women
20-49	0.031	0.038
50-54	0.059	0.051
55-59	0.044	0.064
60-64	0.046	0.061
65-69	0.062	0.066
70-74	0.077	0.085
75-79	0.097	0.109
80-84	0.148	0.157
>84	0.216	0.257

Source: Swedish National Board of Health and Welfare. The Swedish Stroke Statistics, year 2013

Table 11. Death risk CHF.

Age group	men	women
15-49	0	0
50-69	0.057	0.015
70-84	0.245	0.162
>84	0.340	0.281

Source: Swedish National Heart Failure Register, year 2012

The model also incorporates the possibility of dying in unrelated diseases. The death risk in the health state Healthy is the average 5-year age group- and gender-specific risk adjusted for all model disease deaths, the last column in table 12. In disease health states, the risk of dying in unrelated disease is the average 5-year age group- and gender-specific

Table 12. Death risks, unrelated.

Age Group	Not COPD		Not Lung cancer		Not AMI		Not CHF		Not IHD		Not Sudden death		Not Stroke		Not model disease	
	m	w	m	w	m	w	m	w	m	w	m	w	m	w	m	w
<39	0.001	0.000	0.001	0.000	0.001	0.000	0.001	0.000	0.001	0.000	0.001	0.000	0.001	0.000	0.001	0.000
40-44	0.001	0.001	0.001	0.001	0.001	0.001	0.001	0.001	0.001	0.001	0.001	0.001	0.001	0.001	0.001	0.001
45-49	0.002	0.001	0.002	0.001	0.002	0.001	0.002	0.001	0.002	0.001	0.002	0.001	0.002	0.001	0.002	0.001
50-54	0.003	0.002	0.003	0.002	0.003	0.002	0.003	0.002	0.003	0.002	0.003	0.002	0.003	0.002	0.003	0.002
55-59	0.005	0.003	0.004	0.003	0.004	0.003	0.005	0.003	0.004	0.003	0.005	0.003	0.005	0.003	0.005	0.003
60-64	0.008	0.005	0.007	0.005	0.007	0.005	0.008	0.005	0.007	0.005	0.008	0.005	0.008	0.005	0.008	0.005
65-69	0.013	0.008	0.012	0.008	0.012	0.009	0.013	0.009	0.012	0.009	0.013	0.009	0.013	0.009	0.013	0.008
70-74	0.021	0.013	0.020	0.013	0.019	0.014	0.021	0.014	0.020	0.013	0.021	0.014	0.020	0.014	0.021	0.013
75-79	0.037	0.023	0.036	0.023	0.035	0.024	0.037	0.024	0.035	0.023	0.038	0.024	0.036	0.023	0.037	0.023
>79	0.068	0.047	0.068	0.047	0.065	0.047	0.068	0.047	0.065	0.046	0.071	0.048	0.068	0.046	0.068	0.047

m=men, w=women

Source: Swedish National Board of Health and Welfare. The Swedish National Causes of Death Register, year 2014

For peer review only

risk adjusted for the deaths in each respective disease. For ages below 39 years the risk in the age group 35-39 years is used, and for ages 80-84 years the risk >79 years. For ages above 84 years, the general population age-and gender specific death risk is used for the unrelated death risk. As the lung cancer death risks are so high, the unrelated death risks for lung cancer among individuals aged above 84 years had to be adjusted, by deducting 0.05. For those aged below 85 years, the age- and gender-specific general population risk of death is only used for calculating some disease-specific death risks, see tables 2 and 4. The risk is taken from the Swedish national mortality statistics for the year 2014 (Statistics Sweden, 2015).

Changes in risk after quitting smoking

The excess disease risks for smokers are not eliminated immediately after quitting smoking. This "lead time" is 36 years for lung cancer, 16 years for CHD, and 11 years for stroke, while for COPD some excess risk remain life-long, see heading effect of quitting in tables 2 to 4. The disease risks after quitting are constructed by adjusting the smokers' risks by the remaining risk. The remaining risk is modelled as fractions of risk, given the number of years since quitting. The annual remaining risks are estimated by linear interpolation. The effects on the risk for CHD and stroke are modelled on the dummy variable smoking, adjusting the value of 1 by the remaining risk fraction.

The societal costs

The model is reflecting the societal perspective, including disease-related costs for all sectors of the Swedish society. The costs included are medical treatment costs, costs for institutional care and technical aids, pharmaceutical costs, informal care and other patient and relatives' costs, and morbidity productivity costs.

Most of the data on societal costs are taken from Swedish studies published during the 2010s. Data reported as distributions, i.e. with the Gamma parameters for costs, or bootstrapped 95 percent confidence interval were preferred and used in the model to

Table 13. Medical treatment costs. SEK 2014.

	mean	95% confidence interval	distribution	source	comment
Lung cancer	76 096	-	-	KPP register, SALAR 2015	Only inpatient care
COPD	10 120	6 120 - 14 920	-	Jansson et al, 2013	Moderate COPD
AMI year 1	171 660	-	Gamma 106;1622	Henriksson et al, 2014	
AMI year 2+	45 740	-	Gamma 17;2698	Henriksson et al, 2014	
CHF	33 850	-	-	Agvall et al, 2005	
IHD	51 610	-	-	Mourad et al, 2013	Angina pectoris
Stroke year 1	142 280	-	Gamma 114;1244	Henriksson et al, 2014	
Stroke year 2+	38 450	-	Gamma 48;800	Henriksson et al, 2014	

For peer review only - http://bmjopen.bmj.com/site/about/guidelines.xhtml

enable stochastic estimation. If data was reported as mean and standard deviation, the Gamma distribution was simulated employing the Treeage function. In one case, data was reported as fraction of patients consuming a specific resource, which was used for sampling within the model. Otherwise the reported point estimate, usually the average cost across the patient group, was used. If no Swedish data on a cost item was found, the cost was taken from studies reporting data from settings assumed similar to the Swedish.

All costs are reported in SEK year 2014 (USD 1=SEK 6.86; Euro 1=SEK 9.10), adjusted when necessary with the Swedish CPI. To adjust reported Gamma distributed parameters to the price level, only the second parameter, i.e. the scale parameter, was adjusted.

Medical treatment costs

Recent Swedish estimates on medical treatment costs were possible to obtain for all model diseases, see table 13. The costs are paid by the regional healthcare authorities.

Institutional care and technical aids costs

These costs include rehabilitation, terminal care, old age homes, support for individuals living at home, transportation and technical aids. In Sweden, institutional care and technical aids used by patients in their homes are the responsibility of the local authorities (municipalites, in Swedish: kommuner). The costs are not fully represented for any disease, see table 14. Estimates are not available for lung cancer and the only available costs for IHD are outdated, so the institutional care costs are probably underestimated.

Table 14. Costs for institutional care and technical aids. SEK 2014.

	mean	95% confidence interval	distribution	source	comment
Lung cancer	0	-	-		
COPD	0	-	-		Oxygen theraphy included in medical treatment costs
AMI year 1	16 680	-	Gamma 11;1502	Henriksson et al, 2014	Home care and nursing home
AMI year 2+	8 340	-	Gamma 11;751	Henriksson et al, 2014	Home care and nursing home
CHF	2 200	-	-	Agvall et al, 2005	Nursing home
IHD, age <65	3 140	-	-	Andersson & Kartman, 1995	Social services and aids, angina pectoris
IHD, age >64	8 260	-	-	Andersson & Kartman, 1995	Social services and aids, angina pectoris
Stroke year 1	82 130	-	Gamma 11;7184	Henriksson et al, 2014	Home care and nursing home
Stroke year 2+	41 070	-	Gamma 11;3593	Henriksson et al, 2014	Home care and nursing home

For peer review only - http://bmjopen.bmj.com/site/about/guidelines.xhtml

Table 15. Pharmaceutical costs. SEK 2014.

	mean	95% confidence interval	distribution	source	comment
Lung cancer	0	-	-		
COPD	0	-	-		included in medical treatment costs
AMI year 1	11 960	-	-	Mourad et al, 2013	
AMI year 2+	9 250	-	-	Mourad et al, 2013	
CHF	8 420	-	-	Agvall et al, 2005	
IHD	12 690	-	-	Mourad et al, 2013	Angina pectoris
Stroke year 1	2 120	-	-	Ghatnekar et al, 2013	
Stroke year 2+	2 820	-	-	Ghatnekar et al, 2013	

Pharmaceutical costs

Costs for pharmaceuticals in Sweden ought to be divided between the county councils and the patients, as patients pay a considerable share in co-payment. This is however not possible, given the data available. Table 15 therefore presents the drug costs to the regional healthcare authorities. The costs of pharmaceuticals dispensed during hospital stays are included in the medical treatment costs.

Informal care and other patient and relatives' costs

These costs include the value of care given to patients by relatives and other costs for patients or relatives, such as time, co-payments paid for health care and drugs as well as costs for transportation, modifications at home etc. Complete estimates could not be obtained for any disease, see table 16, except IHD which however might be outdated. Informal care in present-day Sweden probably constitute a sizeable part of total societal costs.

Table 16. Informal care and other patient and relatives' costs. SEK 2014.

	Mean	95% confidence interval	distribution	source	comment
Lung cancer	140 810	-	-	Gridelli et al, 2007	Informal care, estimated from 3 months home care
COPD	0	-	-		
AMI year 1	2 090	-	Gamma 44;48	Henriksson et al, 2014	Informal care
AMI year 2+	1 050	-	Gamma 44;24	Henriksson et al, 2014	Informal care
CHF	0	-	-		
IHD, age <65	5 180	-	-	Andersson & Kartman, 1995	Travel and time costs for healthcare contacts, angina pectoris
IHD, age 65+	2 500	-	-	Andersson & Kartman, 1995	Travel and time costs for healthcare contacts, angina pectoris
IHD	680	-	-	Andersson & Kartman, 1995	Informal care, angina pectoris
Stroke year 1	28 260	-	Gamma 44;636	Henriksson et al, 2014	Informal care
Stroke year 2+	14 130	-	Gamma 44;308	Henriksson et al, 2014	Informal care

For peer review only - http://bmjopen.bmj.com/site/about/guidelines.xhtml

Table 17. Productivity costs, morbidity. SEK 2014.

	mean	95% confidence interval	sd	distribution	source	comment
Lung cancer	0	-	-	-	Ford et al, 1999	Simulated in model:<br>9% of pat. 100% disability<br>20% of pat. 80% disability<br>40% of pat. 50% disability<br>31% of pat. 20% disability
					Statistics Sweden	Age- and gender-specific mean wages year 2014
COPD	21 800	6 011 - 42 583	-	-	Jansson et al, 2013	Moderate COPD
AMI year 1	38 180	-	-	Gamma 9;4242	Henriksson et al, 2014	
AMI year 2+	19 090	-	-	Gamma 9;2121	Henriksson et al, 2014	
CHF	29 880	-	49 210	-	Zethraeus et al, 1999	Difference year before and after disease onset
IHD	121 020	-	99 880	-	Mourad et al, 2013	Angina pectoris
Stroke year 1	194 100	-	-	Gamma 9;21567	Henriksson et al, 2014	
Stroke year 2+	97 050	-	-	Gamma 9;10783	Henriksson et al, 2014	

Productivity costs

The productivity costs only value the lost production because of morbidity before the age of 66 years, not mortality. The productivity costs for lung cancer is simulated within the model, via sampling from the fraction of patients on sick leave and combined with age- and gender-specific average monthly wages, including 40% employer taxes. Remaining data is taken from the literature, see table 17, and most estimates are recent. The costs are valued by the human capital method, and thus only include losses in salaried work before the official age of retirement.

The health effects

Life years lost

The number of life years lost (YLS) are calculated until the age of 95 years, and only for individuals dead in the modelled diseases. Life years lost are presented both discounted 3% and undiscounted.

QALYs

The number of quality-adjusted life years (QALYs) are calculated during healthy years and years spent diseased, until death or the age of 95 years.

The QoL weights used during healthy years are mean age group- and gender-specific population weights, see table 18. The data is somewhat dated, but it is the only general population QoL weights available in Sweden. The QoL of the age group 20-29 years is used

For peer review only - http://bmjopen.bmj.com/site/about/guidelines.xhtml

Table 18. Average Swedish population QoL weights.

Age group	men	women
20-29	0.91	0.88
30-39	0.90	0.86
40-49	0.86	0.85
50-59	0.84	0.82
60-69	0.83	0.78
70-79	0.81	0.78
80-88	0.74	0.74

Source: Burström et al, 2001

For peer review only

also for younger ages, and the QoL of the age group 80-88 years is used for those aged 89-95 years. This last assumption is probably an overestimate.

The disease-specific QoL used in the health states are all, except one, modelled as decrements from the average population age-group and gender-specific QoL, see table 19. For lung cancer no data was available on the marginal effect of the disease on the population average QoL, so a fixed value over the ages and genders had to be used.

Sensitivity analyses

Several univariate and multivariate sensitivity analyses have been performed. Analyses on some methodological issues, as well as a probabilistic sensitivity analysis, have also been performed. The analyses are reported for men and women aged 50 years.

To give another measure of the uncertainty surrounding the cost-effectiveness ratio, the 95% confidence interval for the difference between smokers and quitters is reported, calculated from the standard deviation of outcomes.

Table 19. QoL weights and QoL decrements due to disease.

	QoL	source
Health state weight		
Lung cancer	0.653	Nafees et al, 2008
Decrement from average QoL		
COPD	0.0142	Sullivan et al, 2005
AMI	0.0627	Henriksson et al, 2014
CHF	0.0700	Granström et al, 2012
IHD	0.0900	Granström et al, 2012
Stroke	0.1384	Henriksson et al, 2014

For peer review only - http://bmjopen.bmj.com/site/about/guidelines.xhtml

Univariate analyses

Univariate analyses have been performed on all model parameters:

A. disease risks: +100%, -50%

B. death risks: +-10%. (As the unrelated death risks for those aged over 84 years are so high they had to be adjusted by deducting 0.05 for the diseases stroke, IHD and AMI, and omitted for lung cancer, to enable the simulation.)

C. risk fractions of disease after quitting: +-0.1

D. all disease costs: +-25%

E. QoL weights: QoL weight 1 during healthy years

Multivariate analyses

Two sets of multivariate analyses have been performed:

F. high risk – low risk: death risks +100%, disease risks +10% and risk fractions +0.1 *vs* death risks -50%, disease risks –10% and risk fraction –0.1

G. high risk, high costs – low risk, low costs: death risks +100%, disease risks +10%, risk fraction +0.1 and all costs +25% *vs* death risks -50%, disease risks –10%, risk fractions –0.1 and all costs –25%

Analyses on methodological issues

Three analyses have been performed on methodological issues:

H. discount rate: 5%, 0%

I. perspective: healthcare and personal social services perspective (UK NICE perspective); excludes informal care and other patient and relatives' costs and productivity costs

J. recent Swedish data: only includes data from a Swedish context from year 2005 onwards. Excludes the data from Andersson & Kartman (1995) on institutional care and patient and relatives' costs for IHD, from Gridelli et al (2007) on lung cancer patient and relatives' care, from Ford et al (1999) for lung cancer productivity costs and from Zethraeus et al (1999) on CHF productivity costs

Probabilistic analysis

A bootstrap sampling was performed using the smoker and quitter Monte Carlo simulations of 10 000 runs. A sample of 1 000 from each simulation was drawn, with replacement, performed in Microsoft Excel. The mean of the difference in costs and QALYs between smokers and quitters was then calculated. This was replicated 1 000 times. The bootstrap is represented as a scatterplot in the cost-effectiveness plane.

For peer review only

Results

In this chapter, the model estimates of QALYs, YLS and societal costs are presented for men and women in some selected ages, mainly for validation purposes. More detailed simulation outcomes as well as the results of the sensitivity analyses are presented for men and women at age 50 years. Model estimates can be obtained for men and women for all ages between 15 and 95 years.

The model estimates

In table 20 the simulation results for QALYs (quality-adjusted life-years) experienced until the age of 95 years are presented, for the selected ages 15, 30, 50 and 70 years at the start of the simulations. As can be expected, the number of QALYs are highest in the younger age groups, and somewhat higher for women in most age groups. In the selected age groups, the differences between smokers and quitters are at a maximum at age 30; 0.68 for females and 0.81 for males. The confidence intervals, calculated via the mean and standard deviation (sd) from the 10 000 model runs, indicate that there are differences in QALYs between smokers and quitters.

The YLS (life-years saved) lost before the age of 95 years are presented in tables 21 and 22, discounted 3% and undiscounted. The differences in discounted YLS between smokers and quitters are somewhat higher than the differences in QALYs. The undiscounted YLS in table 22 show the number of years that smokers and quitters are expected to lose before the age of 95 years. For the ages 15, 30, and 50 the number of lost life-years is estimated at around 6 years for women smokers and 9 years for men, implying that the female smokers are estimated to live until age 89 and the male until age 86. In the oldest age group presented here, age 70, the number of lost life-years are only 1-2 years. The quitters are estimated to lose considerably fewer life-years; 1-4 years for the women and 3-5 years for

Table 20. QALYs, until age 95 years, discounted 3%.

age	smoker		quitter		difference			
	mean	sd	mean	sd	mean	95% CI		
women								
15	23.20	2.26	23.70	2.28	0.50	0.44	-	0.57
30	20.02	2.85	20.71	2.82	0.68	0.60	-	0.76
50	14.15	4.19	14.76	4.15	0.61	0.49	-	0.73
70	8.24	3.75	8.50	3.82	0.26	0.16	-	0.37
men								
15	23.21	2.84	23.83	2.70	0.63	0.55	-	0.70
30	19.65	3.20	20.46	3.19	0.81	0.72	-	0.90
50	13.18	4.34	13.95	4.47	0.77	0.65	-	0.89
70	6.78	3.61	7.15	3.76	0.37	0.27	-	0.48

For peer review only - http://bmjopen.bmj.com/site/about/guidelines.xhtml

Table 21. Life years lost (YLS), before age 95 years. Discounted 3%.

age	smoker		quitter		difference			
	mean	sd	mean	sd	mean	95% CI		
women								
15	0.97	1.90	0.23	0.87	0.74	0.70	-	0.78
30	1.55	3.02	0.51	1.83	1.04	0.97	-	1.11
50	2.35	4.82	1.49	4.09	0.86	0.74	-	0.99
70	1.22	3.31	0.92	2.98	0.30	0.22	-	0.39
men								
15	1.42	2.25	0.43	1.21	0.99	0.94	-	1.04
30	2.18	3.44	0.79	2.15	1.40	1.32	-	1.48
50	3.51	5.57	2.09	4.69	1.41	1.27	-	1.56
70	2.22	4.30	1.68	3.94	0.53	0.42	-	0.65

the men. As expected, the difference between smokers and quitters diminish with age, with a maximum at around 5 years for the females and around 6 years for the males at age 15.

The societal costs estimated for the smokers and quitters for the selected age groups are presented in table 23. The highest costs are found for age 50; 200 000 SEK and 250 000 SEK for the smokers and 130 000 and 170 000 for the quitters, in both cases higher among the men. The highest difference between smokers and quitters is however found at age 30, with a difference of 100 000 among the females and 120 000 among the males. The difference among the eldest, at age 70, is around 20 000 SEK. These cost differences reflect the amount that tobacco cessation interventions could spend on achieving one quitter and still be cost-saving.

Table 22. Life years lost (YLS), before age 95 years. Undiscounted.

age	smoker		quitter		difference			
	mean	sd	mean	sd	mean	95% CI		
women								
15	6.46	11.80	1.68	5.86	4.78	4.52	-	5.04
30	6.58	11.93	2.22	7.25	4.37	4.09	-	4.64
50	5.67	10.94	3.55	9.19	2.12	1.84	-	2.40
70	1.97	5.18	1.47	4.64	0.50	0.37	-	0.64
men							-	
15	9.25	13.51	3.05	7.89	6.20	5.89	-	6.50
30	9.21	13.39	3.51	8.68	5.70	5.39	-	6.02
50	8.42	12.57	5.01	10.53	3.40	3.08	-	3.73
70	3.56	6.70	2.68	6.11	0.87	0.70	-	1.05

For peer review only

For peer review only - http://bmjopen.bmj.com/site/about/guidelines.xhtml

Table 23. Societal costs. In SEK 2014 and discounted 3%.

age	smoker		quitter		difference			
	mean	sd	mean	sd	mean	95% CI		
women								
15	113 097	278 446	40 761	207 879	72 337	65 526	-	79 147
30	170 047	386 905	71 569	293 477	98 478	88 960	-	107 996
50	201 760	415 452	133 902	366 313	67 858	57 002	-	78 714
70	85 818	189 827	63 824	171 358	21 994	16 981	-	27 006
men								
15	145 233	320 143	54 148	227 222	91 085	83 390	-	98 779
30	216 626	453 147	92 782	349 085	123 844	112 632	-	135 055
50	254 279	484 787	168 598	434 603	85 681	72 920	-	98 442
70	101 358	188 991	80 927	184 794	20 431	15 250	-	25 611

Selected model outcomes

The underlying estimated disease outcome is presented in figures 2 and 3, for the age 50 years. For both women and men, there is a marked decrease for quitters in the number of diseased and dead in the model diseases, which is somewhat offset by an increase in the number of deaths in unrelated diseases. The number of diseased and deaths are higher for

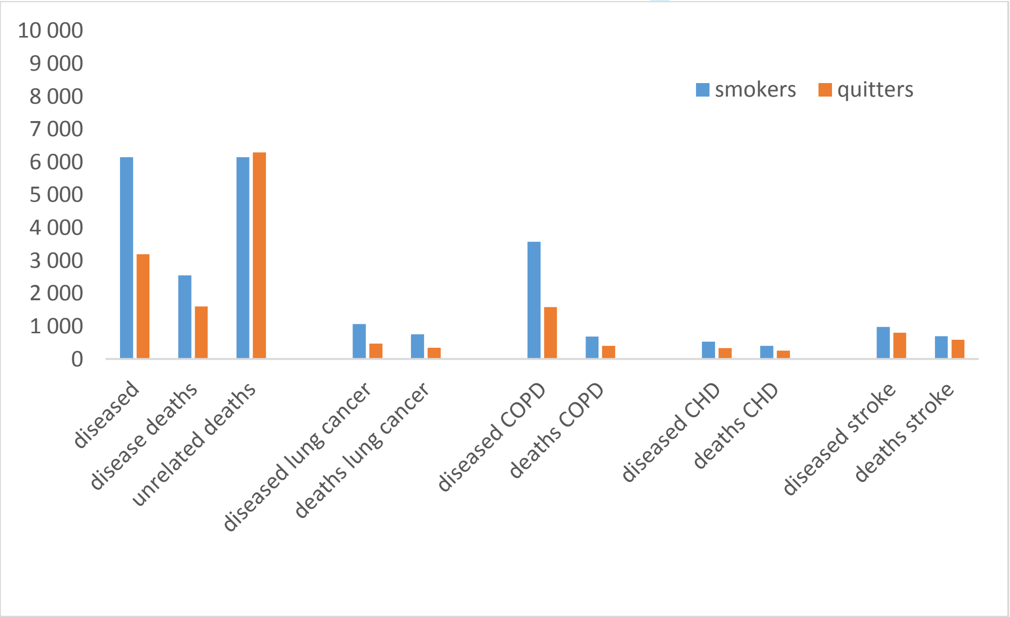

Figure 2. The disease outcome, number of diseased and dead per 10 000 for smokers and quitters, women aged 50 years.

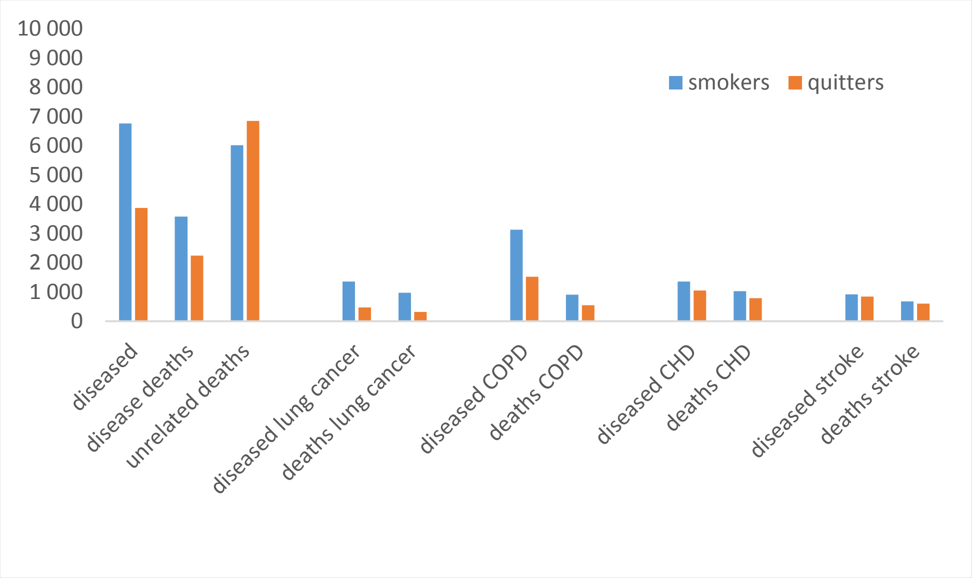

Figure 3. The disease outcome, number of diseased and dead per 10 000 for smokers and quitters, men aged 50 years.

men, mainly originating from CHD. The model disease with the highest smoking-related incidence is COPD, for both genders. The increase in unrelated deaths for the quitters is an example of competing risks, which decreases the difference in life-years and QALYs between smokers and quitters.

Table 24 and 25 shows the full model simulation results of the societal cost savings because of tobacco quitting at age 50 years. For women, the highest estimated savings are found in lung cancer, COPD and stroke at around 15-20 000 SEK per quitter. For men the cost savings because of lung cancer are considerable higher, at around 35 000, due to the higher incidence among the men. The cost item with the largest cost savings are medical treatment costs for both genders, at around 30 000 SEK. Most of the difference in savings between men and women originate from the productivity costs, possibly reflecting disease onset at younger ages among men. Note that a cost saving of zero means that no cost is being modelled, as cost data was lacking.

Table 24. Societal cost savings, in SEK 2014. Women aged 50 years.

	Lung cancer	COPD	AMI	CHF	IHD	Stroke	Sum
Medical treatment	5 171	13 573	2 337	439	3 410	5 500	30 430
Institutional care and technical aids	0	0	365	29	408	4 880	5 681
Pharmaceuticals	0	0	361	109	838	306	1 615
Informal care and other patient and relatives' costs	9 569	0	44	12	282	1 673	11 580
Productivity costs	3 971	6 456	192	243	3 228	4 462	18 552
Sum	18 711	20 029	3 300	832	8 166	16 821	67 858

For peer review only - http://bmjopen.bmj.com/site/about/guidelines.xhtml

Table 25. Societal cost savings, in SEK 2014. Men aged 50 years.

	Lung cancer	COPD	AMI	CHF	IHD	Stroke	Sum
Medical treatment	8 477	11 478	3 203	596	4 738	3 907	32 399
Institutional care and technical aids	0	0	456	39	596	3 379	4 470
Pharmaceuticals	0	0	473	148	1 165	214	2 000
Informal care and other patient and relatives' costs	15 685	0	59	16	377	1 164	17 301
Productivity costs	13 002	8 357	319	400	3 785	3 649	29 511
Sum	37 164	19 835	4 510	1 199	10 661	12 312	85 681

Sensitivity analyses

The results of the sensitivity analyses are presented on women and men at starting age 50 years. Figure 4 shows the results for women and figure 5 for men.

All analyses show a similar pattern between men and women, and also similar ranges. The univariate sensitivity analyses on the model parameters, analyses A to E, result in small changes in costs and QALYs. Also the multivariate analyses F and G, which are constructed as scenarios that allow the risk parameters to vary consistently upwards or downwards, and along with the costs in analysis G, show moderate changes from the base case estimates. The methodological choices have a more pronounced effect, as the largest difference in QALYs is achieved by varying the discount rate (analysis H) between 0 and 5%, which also affects the costs substantially. The two analyses that reflect the choices of which costs to include in the estimates, analysis I that reflects the UK NICE health care and social services perspective and analysis J that only include Swedish data published since the year 2005, both decrease the cost differences between smokers and quitters.

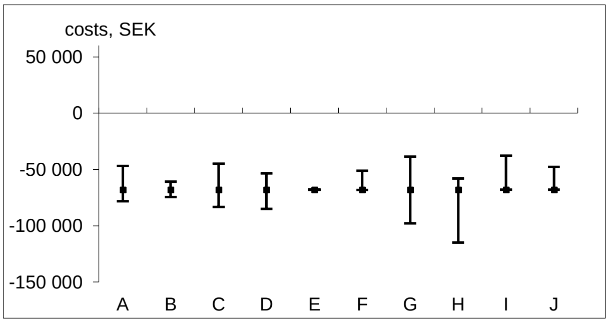

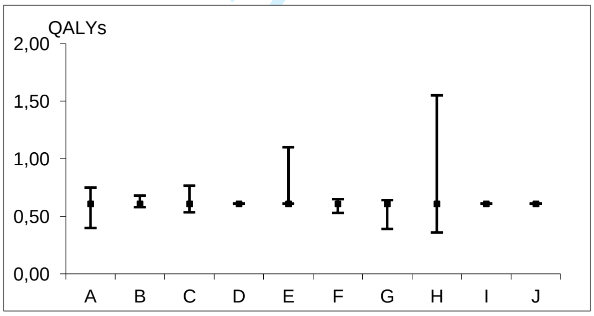

Figure 4. Sensitivity analyses on societal cost and QALY differences between smokers and quitters, women aged 50 years.

Notes: **A**. disease risks. **B**. death risks. **C**. risk fractions of disease after quitting. **D.** all costs. **E**. QoL weights. **F**. high risk – low risk. **G**. high risk, high costs – low risk, low costs. **H**. discount rate. **I**. perspective. **J.** recent Swedish data.

For peer review only - http://bmjopen.bmj.com/site/about/guidelines.xhtml

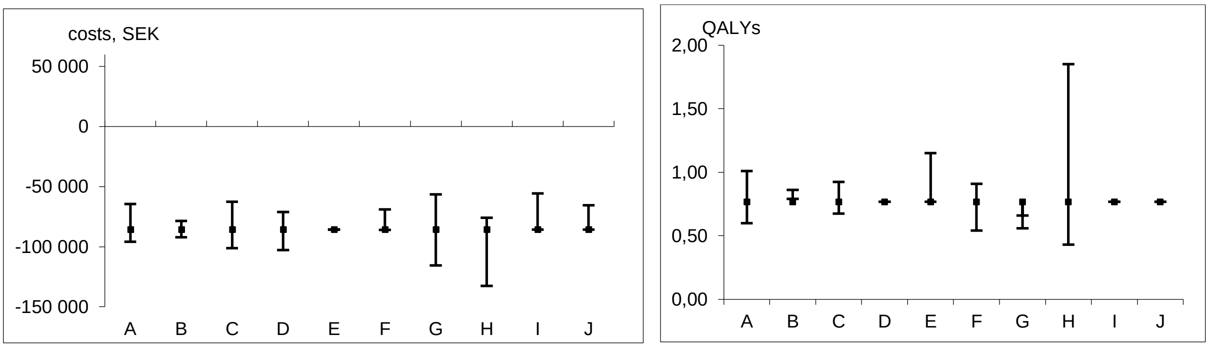

Figure 5. Sensitivity analyses on societal cost and QALY differences between smokers and quitters, men aged 50 years.

Notes: **A**. disease risks. **B**. death risks. **C**. risk fractions of disease after quitting. **D.** all costs. **E**. QoL weights. **F**. high risk – low risk. **G**. high risk, high costs – low risk, low costs. **H**. discount rate. **I**. perspective. **J.** recent Swedish data.

The scatter plot of the bootstrap analysis based on the microsimulation results for women and men aged 50 are shown in figures 6 and 7. The uncertainty is higher for the men, as the plots are more scattered. All plots are however situated in the cost decrease and QALY increase quadrant, with costs below -20 000 SEK and QALYs over 0.2.

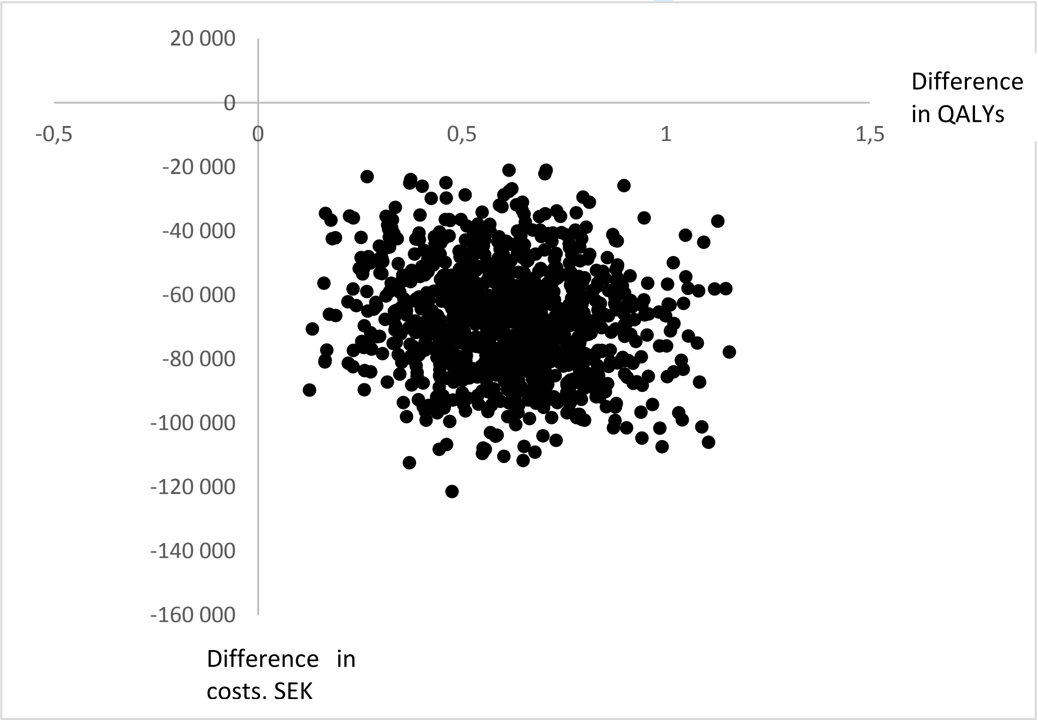

Figure 6. The cost-effectiveness plane with resultat från bootstrap, women aged 50 years.

For peer review only - http://bmjopen.bmj.com/site/about/guidelines.xhtml

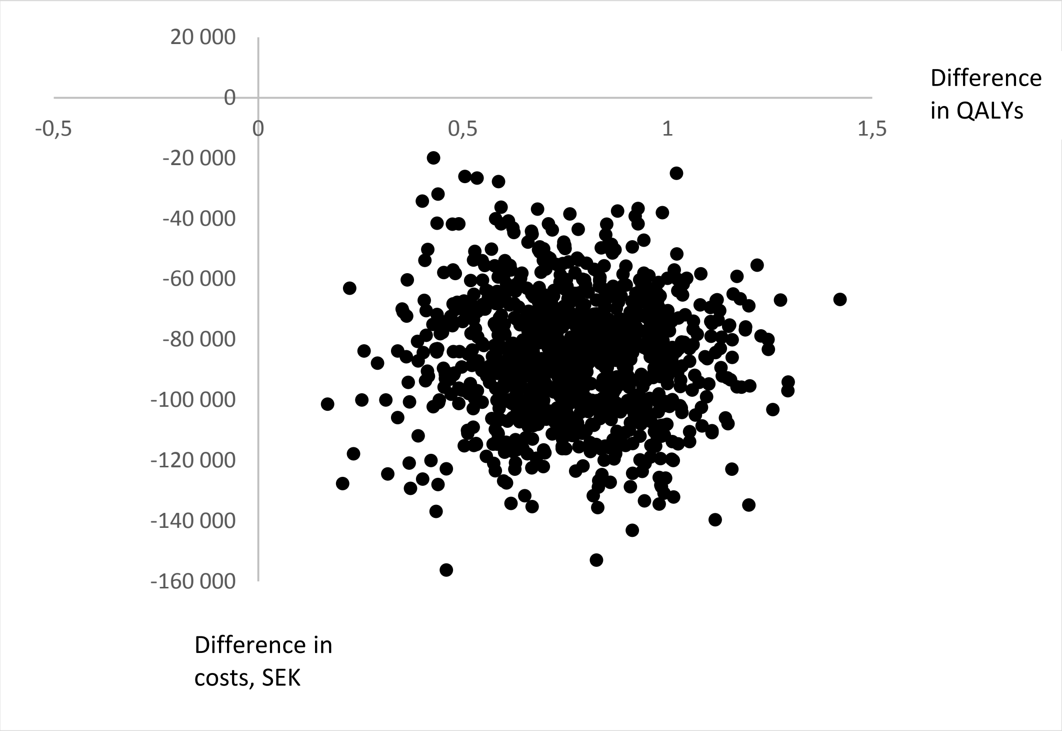

Figure 7. The cost-effectiveness plane with resultat from bootstrap, men aged 50 years.

For peer review only - http://bmjopen.bmj.com/site/about/guidelines.xhtml

Discussion: Model validity

The discussion of the model validity is structured around four aspects as proposed by McCabe & Dixon (2000): the structure of the model, the inputs to the model, the results of the model and the value of the model to the decision-maker.

The structure of the model

The structure of the model is a Markov model constructed for microsimulations, on the three most smoking-related disease groups; lung cancer, COPD, and CVD including stroke and CHD. The present updated version of the model includes one less CHD disease compared to the first version of the model, as unrecognized acute myocardial infarction now is included in the IHD disease, mainly because the disease definition is rarely used nowadays. Choosing only three disease groups is a clear simplification as smoking is known to cause hundreds of different diseases. The effects from smoking, and thus quitting, are furthermore confined to the individuals themselves; no side-effects on other individuals such as environmental tobacco smoke or smoking uptake are included. These two features leads to an underestimate of the true effects of tobacco quitting.

The same disease-specific approach has been taken by most other tobacco cessation models (Bolin, 2012), even though some of them include more diseases, such as asthma. Another approach would be to use the overall differences in mortality between current, former, and never-smokers taken from large US studies, as some early tobacco cessation models did (Secker-Walker et al, 1997; Tengs et al, 2001). In order not to overestimate the effects of quitting tobacco, we chose to model the smoking-related risk for certain diseases instead, as it is improbable that all differences in mortality and morbidity between smokers and former smokers are due to the smoking habit (Doll et al, 1994).

The model aims to reflect disease onset related to smoking tobacco. As disease in all the three disease groups included in model may be caused by other factors than smoking only the excess risks for smokers are modelled. For the diseases lung cancer and COPD this implies that the risk for smokers found in epidemiological studies is adjusted by the risk found for non-smokers. For the disease group CHD and stroke, where a large fraction of disease onset is caused by other factors than smoking, this adjustment for smokers´ excess risk was performed by setting the other risk factors in the risk function at minimal risk levels. This is an underestimate, as the risk factor levels among smokers can be expected to be at least as elevated as among the general population. The underestimate is aggravated by the fact that the functional form of the risk function results in a multiplier effect of the risk factors.

The present version of the model includes seven health states: lung cancer, COPD, stroke, and CHD divided into four diseases. This is a clear simplification, as the costs and QoL can be expected to vary considerable between patients with different severity levels within the diseases. This is particularly true for COPD which is a chronic progressive disease, i.e. the

For peer review only - http://bmjopen.bmj.com/site/about/guidelines.xhtml

diseased get more severely ill over time. However, a model with 7 health states with accompanying disease-specific death risks, costs and QoL weights is fairly complex as well as data-demanding. For the purposes of this study´s model, the division of diseases into severity levels was not deemed necessary.

An obvious problem with the model, inherent in all Markov models, are the mutually exclusive health states; any individual can only contract one disease, and once diseased the individual never recovers (apart from the very rare 5 year survivors in lung cancer). This feature implies both an overestimate and an underestimate of the true effects. The underestimate stems from the fact that co-morbidity is very common, especially among the individuals with the chronic diseases COPD, CHD, and stroke. The overestimate of costs and effects arise as individuals stay in the health states until death. If the costs and outcomes associated with the health states are taken from severely ill individuals, then these become grossly overestimated. This overestimate is partly offset by the use of separate costs for the first and subsequent years, for all societal costs due to AMI and stroke. In order not to overestimate the numbers of years spent in disease states, the possibility of dying in unrelated diseases is present in all health states. This feature is also included in the CHD Policy Model (Weinstein et al, 1987).

Most tobacco cessation models are built for cohort estimation (Bolin, 2012), but this model is constructed for individual-level estimation using the microsimulation methodology. As the data available admitted a microsimulation structure, e.g. the risk functions, the methodology was chosen as the advantages to model and to obtain a richer data set with results that reflect the heterogeneity of outcomes between individuals was deemed to offset the disadvantages of calculation burden. The use of the software Treeage also facilitates the use of microsimulation. Age- and gender-specific estimates can thus be obtained from the model, between ages 15 and 95 years.

The model stages are one-year long, which seems accurate given the risk estimates and the long time horizon of the model. The reason for the model maximum age of 95 years is the lack of risk estimates for older ages. Some extrapolations of risk estimates to the age of 95 years indeed resulted problematic, as some disease-specific death risks expressed as multipliers of the average age-specific death risk resulted in risks above 1. Further extrapolations beyond the age of 95 years were deemed unnecessary, as most of the relevant differences between smokers and quitters would have arisen by that age.

The inputs of the model

The second aspect of model validity is the inputs of the model. The model contains a large number of data taken from different sources. This is of course a threat to the internal validity of the model, shared with most models. However, the data have been chosen to reflect current Swedish circumstances. The current updated version of the model has exchanged almost all cost data, if more recent estimates were available, and all death risks to recent Swedish register data. As the number of studies on any particular data items are few, no meta-analysis or any other synthesis of data was carried out.

For peer review only - http://bmjopen.bmj.com/site/about/guidelines.xhtml

The disease risks are of course are pivotal for the result. The lung cancer disease risks are probably the best that can be obtained, from a large epidemiological study (Peto et al, 2000). The risk equation used for CHD and stroke is taken from the Framingham studies, and even though there are more recent risk scores developed from the study (D'Agostini et al, 2008), the Anderson et al (1991) risk functions are still frequently employed. The disease COPD has been the subject of a large long term epidemiological study in Sweden, The Obstructive Lung Disease in Northern Sweden (OLIN) (Lundbäck et al, 1991), which is thus the most relevant data source for the model.

In the model, there is an increased risk for a smoking-related disease remaining for some years after the tobacco cessation, in accordance with epidemiological evidence (Surgeon General, 1990; Omenn et al, 1990). The feature is also considered a marker of high quality tobacco cessation models (Bolin et al, 2012).

The majority of the cost data are taken from Swedish studies published during the 2010s. To take fully advantage of the microsimulation structure and to obtain stochastic estimates, the preferred data sources were the ones reported as distributions, i.e. as Gamma parameters or bootstrapped 95 percent confidence intervals. If no Swedish data was found, an international estimate was instead used in order to seek to represent the full societal costs. However, apart from certain cost items and for some of the diseases, the lack of data results in considerable underestimates of the true societal costs. This is particularly true in the cases of the costs for care, both institutional and informal. The institutional care could amount to considerable costs, exemplified by the costs for stroke and AMI patients, see table 14. In particular for lung cancer the lack of data results in considerable underestimates of the true disease-related costs. This is why the possible overestimate of the informal care for the disease, obtained from an Italian study, probably does not bias the overall result. To investigate the issue, one sensitivity analysis only included recent Swedish data. The analysis lead to decreases in cost savings for quitters aged 50 years of around 30%.

The QoL estimates are constructed as disease-specific decrements from the average age- and gender-specific QoL, except for lung cancer for which no QoL decrement could be found (De Geer et al, 2013). The average population age- and gender-specific QoL weights, which are certainly not 1, are also used during healthy years for the base case estimates. This means that the model assumes that an individual that avoids the smoking-related diseases is not having perfect health, but the health of an average Swede at the same age, as recommended (Gold et al, 1996).

The stated purpose of the model is to reflect the societal perspective, which for Sweden includes the morbidity productivity costs, but not the productivity costs resulting from mortality. All the model data on productivity costs value them according to the human capital approach for individuals under the age of 65, the customary Swedish age of retirement.

A full societal perspective might also include other aspects, considering that this is a model on individuals that are participating in an intervention that aims to change their lifestyle. The previous version of the tobacco cessation model, version 1 (Johansson, 2004), reported

For peer review only - http://bmjopen.bmj.com/site/about/guidelines.xhtml

sensitivity analyses that modelled some effects on the tobacco quitters, by including savings from cigarette purchases and a decreased QoL because of withdrawal effects during the first year. When that analysis was applied to an intervention, a decreased QoL during the first year was also deducted for the smokers that failed to quit, as the failure to achieve a personal goal might to lead to a decrease in QoL.

The results of the model

The third aspect of model validity is the results of the model, e.g. a comparison with reality or with other study results. A direct comparison with reality is not possible, since the model covers the ages 15-95 years, with a follow-up time of 80 years for the youngest age group.

The model estimates that around 60% of the women and 70% of the men aged 50 at the start of the simulations will contract one of the modelled diseases, and that around 50% of those will die in the diseases before the age of 95 years. The disease risks for the quitters at age 50 are not eliminated; 30-40% of them will still contract the smoking-related diseases because of remaining disease risks after quitting. As expected, the unrelated deaths increase among the quitters, in sum leading to an increase in YLS (undiscounted) of 2-3 years for those quitting at age 50, compared with continuing smokers. The increases in QALYs (discounted 3%) are smaller because of less-than-perfect health among those aged 50 years and above; 0.61 for women and 0.77 for men. The disease outcomes are fairly similar to the estimates from the previous versions of the model, but because of decreased death risks, the outcomes in terms of YLS and QALYs are considerably higher. The 2004 version of the model estimated an increased YLS of 0.93 and of 1.66 for women and men aged 50-54 years, and QALY gains of 0.36 and 0.71, respectively. The differences are due to the longer time perspective of the present version, 95 years versus 85 years, and the somewhat decreased case-fatality risk (i.e. the mortality risk among those with disease) because of improvements in medical technologies during the past decade.

Apart from increases in health, the societal cost savings because of quitting smoking are considerable. For men, the cost savings amount to around 100 000 SEK for quitters aged between 15 and 50 years, and around 70-90 000 SEK for women. Even in the age group 70 years there are estimated cost savings of around 20 000 SEK per individual quitter. This implies that substantial funds could be invested in smoking cessation interventions, and the interventions would still be cost-effective, or even cost-saving. The cost savings in the present model are considerably higher than those of the previous model, in part due to changes in price year.

Comparisons of model estimates with other models' are difficult to perform, as the time horizon, costs included, jurisdiction, and the diseases included differ. Among the recently reported model estimates (Bolin, 2012), there are two Australian models. The model developed within ACE (Bertram et al, 2007) report estimates of life-years saved that are considerable higher than the present model's; 5.7 years for men and 6.6 years for women in age group 50-54 years. That model time horizon is however 100 year, but it is unlikely

For peer review only - http://bmjopen.bmj.com/site/about/guidelines.xhtml

that the feature fully explains the difference between the model estimates. The estimates of average health care cost saved per quitter (inferred from table 3) however seems to be very similar to the present model's; around 33 000 SEK. The other Australian model, the Quit Benefits Model (Hurley & Mathews, 2007), reports considerably lower estimates of both life-years and health care costs saved, e.g. 0.1 – 0.2 YLS and QALYs saved for men and women quitters. The lower estimates, in comparison with both the present model and the ACE model, are probably partly explained by the time horizon of only ten years.

There have been two, to my knowledge, reports of tobacco cessation model estimates for Sweden, one using the Benesco model (Bolin et al, 2007) and one using an extended version of the HECOS model (Bolin et al, 2006). Comparison with those model estimates are unfortunately not possible, due to lack of reporting detail. However, estimates from the previous version of this model were fairly consistent with the HECOS model estimates (Orme et al, 2001) for Sweden, available at the time (Johansson, 2004).

The value of the model to the decision-maker

The fourth aspect of validity is the value of the model to the decision-maker. There are several models on tobacco cessation that conforms to international recommendations on how to perform cost-effectiveness analyses (Bolin, 2012). This model however reflect Swedish circumstances, with Swedish cost and QoL data, why the model might be useful for Swedish decision-makers.

We hope that the model will be used to perform economic evaluations of a range of tobacco cessation interventions. For tobacco prevention interventions, i.e. prevention of initiation of smoking, another model version, version 2, has been constructed and is available for analyses. The use of these models will in time enable incremental and marginal calculations of the cost-effectiveness of different tobacco interventions and their components and suitable target groups. The basis for decisions on which tobacco cessation and prevention interventions to implement will then be more comprehensive.

Another frequent use of models is to forecast future events. This model is not suitable for estimating what the costs of smoking will be in the future. The reason is that the model does not incorporate any adjustments of possible future developments. The risk of smoking is based on studies with follow-up periods of sometimes 30 years, which means that the risks are reflecting the smoking behaviour among smokers 30 years ago. The changes in cigarette content and in the frequency of smoking might lead to changes in disease risk in the future. Also the costs for the smoking-related diseases might change in the future, because of changes in health care technology. Another example would be the value of the morbidity productivity costs, as well as informal care, as wages and productivity often are expected to increase in the future.

Nevertheless, the model actually forecasts what the costs for smokers and quitters will be in 80 years' time, for the youngest age group. That implies that we know that the model forecasts will be wrong, but it is of minor significance as the model is constructed to be used for comparisons between two groups, smokers and quitters, thus eliminating some

For peer review only - http://bmjopen.bmj.com/site/about/guidelines.xhtml

of the biases. Furthermore, the model is constructed to be used now, for present-day decisions, which have to be based on present-day information.

The uncertainty

Another aspect of model validity is the uncertainty surrounding the model estimates.

The univariate sensitivity analyses on the model parameters (analyses A-F in figures 4 and 5 for men and women aged 50) show minor deviations from the base case result, while the multivariate analysis on costs and risks combined (analysis G) affects in particular the cost estimates. The methodological choices affect the results to a greater extent, with the discount rate (H) heavily influencing the QALYs and the more restricted perspective (I) decreasing the cost-savings. The multivariate analysis that only include higher-quality data (J) also imply decreases in the cost differences between smokers and quitters, but the difference remains substantial; around 50 000 SEK for females aged 50 years and 60 000 SEK for men, respectively. The overall conclusion from the parameter sensitivity analyses is that the QALY gains are at least 0.35 and 0.40 and the cost savings at least SEK 35 000, for female and male quitters aged 50, respectively.

The probabilistic analysis shows no uncertainty whether quitting tobacco leads to cost-savings and increases in QALYs, as all bootstraps are placed in the southeast quadrant of the cost-effectiveness plane. The bootstrap results exhibit a mixture of first and second order uncertainty, as it reflects both the probabilistic structure of the Markov model and the simulation of some parameter values (Briggs, 2000).

Another measure of uncertainty is the confidence intervals around the estimated mean differences, reported in tables 20-23. However, that measure is not fully appropriate as the large sample sizes of the Monte Carlo simulation (10 000 runs) diminishes the standard error of the mean (Briggs, 2000).

The structural uncertainty of the model, i.e. whether the results would be different if the model would have been constructed in another way, have not been studied. Alternatives to the chosen model structure could have been deterministic or discreet event simulations, more or less health states, other functional forms of risk functions, and other subgroups than men and women and five-year age-groups model results. The flaw is however shared with most tobacco quitting models (Bolin, 2012).

Checking for technical errors

The model contains a large number of trackers, i.e. variables that count events, to enable checking for technical errors. Tentative runs were executed after the introduction of every new variable, with cost items undiscounted, and the simulation results examined manually. Thus, the model has been thoroughly checked for technical errors.

For peer review only - http://bmjopen.bmj.com/site/about/guidelines.xhtml

Conclusions

The aim of this study is to develop a model predicting health and economic consequences of smoking cessation, to be used for cost-effectiveness analyses of smoking cessation interventions. The updated model strives to incorporate data that is recent, accurate and appropriate for Sweden in year 2015. The model also adhere to Swedish recommendations on how to perform cost-effectiveness analyses within the health care sector. Data is however lacking to completely fulfil these requirements. Many model parameters are based on very few studies. Some information just does not exist, at least not accessible to us.

These are issues shared with most model, however. The purpose of modelling is to assemble the most accurate information at a point of time, to enable decision-making at that particular point of time. This is in accordance with one of the fundamentals of economics: decision-making under uncertainty, which implies that decisions have to be made even if there is no full information. We hope that the model will be applied to a range of different tobacco cessation interventions, which in time will enable a more comprehensive basis for decision-making.

For peer review only

For peer review only - http://bmjopen.bmj.com/site/about/guidelines.xhtml

References

Agvall B, Borgquist L, Foldevi M, Dahlström M. Cost of heart failure in Swedish primary healthcare. Scandinavian Journal of Primary Health Care 2005;23:227-232.

Anderson KM, Odell PM, Wilson PWF, Kannel WB. Cardiovascular disease risk profiles. American Heart Journal 1991;121:293-298.

Andersson F, Kartman B. The cost of angina pectoris in Sweden. Pharmacoeconomics 1995;8:233-44.

Bertram MY, Lim SS, Wallace AL, Vos T. Costs and benefits of smoking cessation aids: making a case for public reimbursement of nicotine replacement therapy in Australia. Tobacco Control 2007; 16:255-260.

Bolin K. Economic evaluation of smoking-cessation therapies -A critical and systematic review of simulation models. Pharmacoeconomics 2012;30: 551-556.

Bolin K, Lindgren B, Willers S. The cost utility of bupropion in smoking cessation health programs. Simulation model results for Sweden. Chest 2006;129:651-660.

Bolin K, Wilson K, Benhaddi H, de Nigris E, Marbaix S, Mörk A-C, Aubin H-J. Cost-effectiveness of varenicline compared with nicotine patches for smoking cessation –results from four European countries. European Journal of Public Health 2007;19:650-654.

Briggs A, Sculpher M. An introduction to Markov modelling for economic evaluation. Pharmacoeconomics 1998;13:397-409.

Briggs AH. Handling uncertainty in cost-effectiveness models. Pharmacoeconomics 2000;17:479-500.

Burström K, Johannesson M, Diderichsen F. Swedish population health-related quality of life results using the EQ-5D. Quality of Life Research 2001;10:621-635.

Caro JJ, O´Brien JA, Hollenbeak CS, Spackman E, Ben-Joseph R, Okamoto LJ, Paramore LC. Economic burden and risk of cardiovascular disease and diabetes in patients with different cardiometabolic risk profiles. Value in Health 2007;10:S12-S20.

D'Agostino RB, Vasan RS, Pencina MJ, Wolf PA, Cobain M, Massaro JM, Kannel WB. General cardiovascular risk profile for use in primary care: The Framingham Heart Study. Circulation 2008;117:743-753.

De Geer A, Eriksson J, Finnern HW. A cross-country review of data collected on non-small cell lung cancer (NSCLC) patients in cancer registries, databases, retrospective and non-randomized prospective studies. Journal of Medical Economics 2013:16; 134–149.

Doll R, Peto R, Wheatley K, Gray R, Sutherland I. Mortality in relation to smoking: 40 years' observation on male British doctors. BMJ 1994;309:901-911.

Drummond MF, Sculpher MJ, Torrance GW, O´Brien B, Stoddart GL. Methods for the economic evaluation of health care programmes. (3rd edition). Oxford: Oxford University Press; 2005.

For peer review only - http://bmjopen.bmj.com/site/about/guidelines.xhtml

Ford ES, Kelly AE, Teutsch SM, Thacker SB, Garbe PL. Radon and lung cancer: A cost-effectiveness analysis. American Journal of Public Health 1999;89:351-357.

Ghatnekar O, Asplund, K, Glader E-K, Persson U. Costs for stroke in Sweden 2009 and developments since 1997. International Journal of Technology Assessments in Health Care 2014;30:203–209.

Gold MR, Siegel JE, Russell LB, Weinstein MC, eds. Cost-effectiveness in health and medicine. New York: Oxford University Press; 1996.

Granström O, Levin L-Å, Henriksson M. Cost-effectiveness of candesartan versus losartan in the primary preventive treatment of hypertension. ClinicoEconomics and Outcomes Research 2012;4:313–322.

Gridelli C, Ferrara C, Guerriero C, Palazzo S, Grasso G, Pavese I, Satta F, Bajetta E, Cortinovis D, Barbieri F, Gebbia V, Grossi F, Novello S, Baldini E, Gasparini G, Latino W, Durante E, Giustini L, Negrini C. Informal caregiving burden in advanced non-small cell lung cancer: The HABIT study. Journal of Thoracic Oncology 2007;2:475–480.

Henriksson M, Nikolic E, Ohna A, Wallentin L, Janzon M. Ticagrelor treatment in patients with acute coronary syndrome is cost-effective in Sweden and Denmark. Scandinavian Cardiovascular Journal 2014;48:138–147.

Holm LE, Gunnarskog J, Stenbeck M. In Stenbeck M, Rosén M (eds). Cancer survival in Sweden in 1961-1991. Acta Oncologica 1995; 34(suppl 4): 39-47.

Jansson S-A, Backman H, Stenling A, Lindberg A, Rönmark E, Lundbäck B. Health economic costs of COPD in Sweden by disease severity –has it changed during a ten years period? Respiratory Medicine 2013;107:1931-1938.

Johansson P. A model for economic evaluations of smoking cessation interventions – technical report. Version 1. Centre for Public Health, Stockholm county council; 2004. Available at: http://www.publichealtheteconomics.se/wp-content/uploads/2016/06/smoking-cessation-cea-model.pdf

Lindberg A, Eriksson B, Larsson L-G, Rönmark E, Sandström T, Lundbäck B. Seven-year cumulative incidence of COPD in an age-stratified general population sample. Chest 2006;129:879–885.

Lundbäck B, Nyström L, Rosenhall L, Stjernberg N. Obstructive lung disease in northern Sweden: respiratory symptoms assessed in a postal review. European Respiratory Journal 1991; 4:257–266.

Lundbäck B, Eriksson B, Lindberg A, Ekerljung L, Muellerova H, Larsson L-G, Rönmark E. A 20-year follow-up of a population study-based COPD cohort -Report from the Obstructive Lung Disease in Northern Sweden Studies. COPD 2009;6:4,263-271.

McCabe C, Dixon S. Testing the validity of cost-effectiveness models. Pharmacoeconomics 2000;17:501-513.

Mourad G, Alwin J, Strömberg A, Jaarsma T. Societal costs of non-cardiac chest pain compared with ischemic heart disease –a longitudinal study. BMC Health Services Research 2013;13:403.

For peer review only - http://bmjopen.bmj.com/site/about/guidelines.xhtml

Nafees B, Stafford M, Gavriel S, Bhalla S, Watkins J. Health state utilities for non small cell lung cancer. Health and Quality of Life Outcomes 2008;6:84.

Omenn GS, Anderson KW, Kronmal RA, Vlietstra RE. The temporal pattern of reduction of mortality risk after smoking cessation. American Journal of Preventive Medicine 1990;6:251-257.

Orme ME, Hogue SL, Kennedy LM, Paine AC, Godfrey C. Development of the health and economic consequences of smoking interactive model. Tobacco Control 2001;10:55-61.

Peto R, Darby S, Deo H, Silcocks P, Whitley E, Doll R. Smoking, smoking cessation, and lung cancer in the UK since 1950: combination of national statistics with two case-control studies. BMJ 2000;321:323-9. (Data supplement).

Secker-Walker RH, Worden JK, Holland RR, Flynn BS, Detsky AS. A mass media programme to prevent smoking among adolescents: costs and cost effectiveness. Tobacco Control 1997;6:207-212.

Sonnenberg FA, Beck JR. Markov models in medical decision making: A practical guide. Medical Decision Making 1993;13:322-38.

Statistics Sweden (SCB). Statistics database. www.statistikdatabasen.scb.se Accessed October 2015.

Sullivan PW, Lawrence WF, Ghushchyan V. A national catalogue of preference-based scores for chronic conditions in the United States. Medical Care 2005;43:736-749.

Surgeon General. The health benefits of smoking cessation. U.S. Department of Health and Human Services, Public Health Service, Centres for Disease Control, Center for Chronic Disease Prevention and Health Promotion, Office on Smoking and Health. DHHS Publication No. (CDC) 90-8416. 1990.

Swedish Association of Local Authorities and Regions (SALAR). KPP- database. Accessed 2015-10-01 at https://stat.skl.se/kpp/index.htm

Swedish National Board of Health and Welfare. Statistics database. Diagnoses in inpatient care from the Hospital Discharge Register. Accessed 2015-10-01 at www.socialstyrelsen.se/statistik/statistikdatabas/diagnoserislutenvard

Swedish National Board of Health and Welfare. The Swedish AMI Statistics. Accessed 2015-10-01 at www.socialstyrelsen.se/statistik/statistikdatabas/hjartinfarkter

Swedish National Board of Health and Welfare. The Swedish Stroke Statistics. Accessed 2015-10-01 at www.socialstyrelsen.se/statistik/statistikdatabas/stroke

Swedish National Board of Health and Welfare. The Swedish Causes of Death Register. Accessed 2015-10-01 at www.socialstyrelsen.se/statistik/statistikdatabas/dodsorsaker

Swedish National Heart Failure Register. Accessed 2015-10-01 at http://www.ucr.uu.se/rikssvikt/

Talbäck M, Rosén M, Stenbeck M, Dickman PW. Cancer patient survival in Sweden at the beginning of the third millennium -Predictions using period analysis. Cancer Causes and Control 2004;15:967-976. Accessed 2015-10-10 at www.socialstyrelsen.se/statistics/cancersurvival

For peer review only - http://bmjopen.bmj.com/site/about/guidelines.xhtml

Tengs TO, Osgood ND, Chen LL. The cost-effectiveness of intensive national school-based anti-tobacco education: Results from the Tobacco Policy Model. Preventive Medicine 2001;33:558-570.

TLV (Swedish Dental and Pharmaceutical Benefits Agency). General guidelines for economic evaluations from the Pharmaceutical Benefits Board (LFNAR 2003:2). Accessed 2016-04-24 at http://tlv.se/Upload/English/Guidelines-for-economic-evaluations-LFNAR-2003-2.pdf

Weinstein MC, Coxson PG, Williams LW, Pass TM, Stason WB, Goldman. L. Forecasting coronary heart disease incidence, mortality and cost: The Coronary Heart Disease Policy Model. American Journal of Public Health 1987;77:1417-1426.

Zethraeus N, Molin T, Henriksson P, Jönsson B. Costs of coronary heart disease and stroke: the case of Sweden. Journal of Internal Medicine 1999;246:151-159.

For peer review only

Consolidated Health Economic Evaluation Reporting Standards (CHEERS) statement

Section/item	Item No	Recommendation	Reported on page No/ line No
**Title and abstract**			
Title	1	Identify the study as an economic evaluation or use more specific terms such as "cost-effectiveness analysis", and describe the interventions compared.	Title, page 1
Abstract	2	Provide a structured summary of objectives, perspective, setting, methods (including study design and inputs), results (including base case and uncertainty analyses), and conclusions.	Abstract, page 3
**Introduction**			
Background and objectives	3	Provide an explicit statement of the broader context for the study. Present the study question and its relevance for health policy or practice decisions	Page 6, lines 1-17
**Methods**			
Target population and subgroups	4	Describe characteristics of the base case population and subgroups analysed, including why they were chosen.	Page 6-8
Setting and location	5	State relevant aspects of the system(s) in which the decision(s) need(s) to be made.	Page 3 lines 14-17<br>Page 9, lines 7-8
Study perspective	6	Describe the perspective of the study and relate this to the costs being evaluated.	Page 9, lines 17-24
Comparators	7	Describe the interventions or strategies being compared and state why they were chosen.	Page 7. lines 3-8,
Time horizon	8	State the time horizon(s) over which costs and consequences are being evaluated and say why appropriate.	Page 12, lines 2-13
Discount rate	9	Report the choice of discount rate(s) used for costs and outcomes and say why appropriate.	Page 9, line 24
Choice of health outcomes	10	Describe what outcomes were used as the measure(s) of benefit in the evaluation and their relevance for the type of analysis performed.	Page 10, lines 17-23
Measurement of effectiveness	11a	*Single study-based estimates:* Describe fully the design features of the single effectiveness study and why the single study was a sufficient source of clinical effectiveness data.	Page 8, table 1

For peer review only

Section/item	Item No	Recommendation	Reported on page No/ line No
	11b	*Synthesis-based estimates*: Describe fully the methods used for identification of included studies and synthesis of clinical effectiveness data.	Not applicable
Measurement and valuation of preference based outcomes	12	If applicable, describe the population and methods used to elicit preferences for outcomes.	Not applicable
Estimating resources and costs	13a	*Single study-based economic evaluation:* Describe approaches used to estimate resource use associated with the alternative interventions. Describe primary or secondary research methods for valuing each resource item in terms of its unit cost. Describe any adjustments made to approximate to opportunity costs.	Not applicable
	13b	*Model-based economic evaluation:* Describe approaches and data sources used to estimate resource use associated with model health states. Describe primary or secondary research methods for valuing each resource item in terms of its unit cost. Describe any adjustments made to approximate to opportunity costs.	Page 11, lines 21-25<br>Page 12, lines 1-14<br>Appendix 1
Currency, price date, and conversion	14	Report the dates of the estimated resource quantities and unit costs. Describe methods for adjusting estimated unit costs to the year of reported costs if necessary. Describe methods for converting costs into a common currency base and the exchange rate.	Page 9, lines 17-20
Choice of model	15	Describe and give reasons for the specific type of decision-analytical model used. Providing a figure to show model structure is strongly recommended.	Page 11, lines 21-25<br>Page 12, lines 1-6<br>Appendix 1
Assumptions	16	Describe all structural or other assumptions underpinning the decision-analytical model.	Appendix 1
Analytical methods	17	Describe all analytical methods supporting the evaluation. This could include methods for dealing with skewed, missing, or censored data; extrapolation methods; methods for pooling data; approaches to validate or make adjustments (such as half cycle corrections) to a model; and methods for handling population heterogeneity and uncertainty.	Page 12, lines 15-20<br>Appendix 1
**Results**			

For peer review only

Section/item	Item No	Recommendation	Reported on page No/ line No
Study parameters	18	Report the values, ranges, references, and, if used, probability distributions for all parameters. Report reasons or sources for distributions used to represent uncertainty where appropriate. Providing a table to show the input values is strongly recommended.	Appendix 1
Incremental costs and outcomes	19	For each intervention, report mean values for the main categories of estimated costs and outcomes of interest, as well as mean differences between the comparator groups. If applicable, report incremental cost-effectiveness ratios.	Page 15, Table 3<br>Page 17, Table 4
Characterising uncertainty	20a	*Single study-based economic evaluation:* Describe the effects of sampling uncertainty for the estimated incremental cost and incremental effectiveness parameters, together with the impact of methodological assumptions (such as discount rate, study perspective).	Not applicable
	20b	*Model-based economic evaluation:* Describe the effects on the results of uncertainty for all input parameters, and uncertainty related to the structure of the model and assumptions.	Page 17, lines 14-24<br>Page 18, lines 1-9<br>Page 18, figure 1
Characterising heterogeneity	21	If applicable, report differences in costs, outcomes, or cost-effectiveness that can be explained by variations between subgroups of patients with different baseline characteristics or other observed variability in effects that are not reducible by more information.	Not applicable
**Discussion**			
Study findings, limitations, generalisability, and current knowledge	22	Summarise key study findings and describe how they support the conclusions reached. Discuss limitations and the generalisability of the findings and how the findings fit with current knowledge.	Pages 18-20
**Other**			
Source of funding	23	Describe how the study was funded and the role of the funder in the identification, design, conduct, and reporting of the analysis. Describe other non-monetary sources of support.	Page 21 "Funding"
Conflicts of interest	24	Describe any potential for conflict of interest of study contributors in accordance with journal policy. In the absence of a journal policy, we recommend authors comply with International Committee of Medical Journal Editors recommendations.	Page 21 "Competing interests"

The CHEERS statement checklist format is based on the format of the CONSORT statement checklist

For peer review only

For peer review only

BMJ Open

Cost-effectiveness of a high- vs a low-intensity smoking cessation intervention in a dental setting: long-term follow up

Journal:	*BMJ Open*
Manuscript ID	bmjopen-2019-030934.R2
Article Type:	Research
Date Submitted by the Author:	10-Jul-2019
Complete List of Authors:	Feldman, Inna; Uppsala Universitet, Department of Public Health and Caring Science<br>Helgason, Asgeir; Karolinska Institutet, Department of Public Health Sciences, Social Medicine<br>Johansson, Pia; Public Health & Economics<br>Tegelberg, åke; Centre for Clinical Research, Uppsala University, Hospital of Vastmanland<br>Nohlert, Eva; Centre for Clinical Research, Uppsala University and Region Vastmanland,
**Primary Subject Heading**:	Health economics
Secondary Subject Heading:	Dentistry and oral medicine, Health services research
Keywords:	smoking cessation, cost-effectivenes, long-term sustainability

SCHOLARONE™
Manuscripts

1

Cost-effectiveness of a high- vs a low-intensity smoking cessation intervention in a dental setting: long-term follow up.

Inna Feldman[1], Ásgeir R Helgason[2.3], Pia Johansson[4], Åke Tegelberg[5.6] and Eva Nohlert[5]

[1] - Department of Public Health and Caring Sciences, Uppsala University, Uppsala, Sweden

[2] - Karolinska Institutet, Stockholm, Sweden

[3] - Reykjavik University & Icelandic Cancer Society, Reykjavik, Iceland

[4] - Public Health & Economics, Kyrkängsbacken 8, Huddinge, Sweden

[5] - Centre for Clinical Research, Uppsala University, Hospital of Västmanland, Västeras. Sweden

[6] - Faculty of Odontology, Malmö University, Malmö, Sweden

Corresponding author: Inna Feldman. inna.feldman@pubcare.uu.se

For peer review only

2

Abstract

Objectives. The aim of this study was to conduct a cost-effectiveness analysis of a high- and a low-intensity smoking cessation treatment programme (HIT and LIT) using long-term follow-up effectiveness data and to validate the cost-effectiveness results based on short-term follow-up.

Design and outcome measures. Intervention effectiveness was estimated in a randomized controlled trial as numbers of abstinent participants after 1 and 5–8 years follow-up. The economic evaluation was performed from a societal perspective using a Markov model by estimating future disease-related costs (in Euro (€) 2018) and health effects (in quality-adjusted life-years, QALYs). Programmes were explicitly compared in an incremental analysis, and the results were presented as an incremental cost-effectiveness ratio (ICER).

Setting. Dental clinics in Sweden.

Participants. 294 smokers aged 19–71 years.

Interventions. Behaviour therapy, coaching and pharmacological advice (HIT) was compared with one counselling session introducing a conventional self-help programme (LIT).

Results. The more costly HIT led to higher number of 6-month continuous abstinent participants after 1 year and higher number of sustained abstinent participants after 5–8 years, which translates into larger societal costs avoided and health gains than LIT. The incremental cost/QALY of HIT compared to LIT amounted to €918 and €3,786 using short- and long-term effectiveness respectively, which is considered very cost-effective in Sweden.

Conclusion. Cost-effectiveness analysis favours the more costly HIT if decision-makers are willing to spend at least €4,000/QALY for tobacco cessation treatment.

For peer review only

Strengths and limitations of this study

- This study utilises a unique possibility to compare cost-effectiveness analyses based on 1-year and 5-8 years follow-up data.
- This economic evaluation clearly supports that more intensive and costly smoking cessation provision is cost-effective.
- The calculation of the intervention costs for the cessation programmes was based on a trial protocol and might be overestimated in comparison with routine practice.
- The effects of smoking cessation are probably underestimated since only three disease groups are modelled and no effects of passive smoking are included.

For peer review only

Introduction

Smoking is likely to remain the single most important preventable health risk in the world. Despite continuously declining prevalence in recent decades, one in ten adults in Sweden still smokes daily [1]. Cigarette smoking contributes to 7.5% of the burden of disease in Sweden [2] and was estimated to account for approximately €3,000,000 (31.5 billion Swedish krona, SEK), including €1,000,000 (11 billion SEK) in healthcare costs (15% of the national costs for health and welfare sector) and €1,500,000 (16 billion SEK) in productivity costs in year 2015 [3]. A decrease in prevalence of smoking to five per cent could save society €1,300,000 (14.3 billion SEK) per year.

Several smoking cessation interventions, targeted at current smokers, are available; furthermore, evaluations so far have confirmed the effectiveness of the majority of them. Additionally, some recent studies emphasise that higher level of intervention intensity, such as additional counselling sessions [4] and intensive support through a mobile application [5], resulted in the highest smoking cessation rates. However, due to increasing number of available interventions, decision-makers have to decide which intervention to implement, taking into account that intervention intensity increases intervention costs. Relative costs and benefits of those interventions are important criteria, thus, increasing the attention on economic evaluations in recent years [6 7]. Economic evaluations combine the costs and outcomes of different interventions and aim to determine which intervention provides the best value for money [8]. Several studies on the cost-effectiveness of smoking cessation interventions comparing different intensity of support have been performed during the last few years. For example, Quit-and-Win programme [9], comparison of standard, enhanced and intensive smoking cessation interventions using cell phones [10], and two smoking cessation

For peer review only

approaches of different level of intensity for cancer patients [11]. The results suggested that the higher intensive interventions are preferable from health economics point of view, but all those evaluations were based on 6- or 12-months follow-up, long-term follow-ups are scarce in randomised controlled trials.

The effects of smoking on health occur during many years because current smoking influences future health risks; similarly, a smoking cessation today will cause smoking related health risks to tail off gradually. Thus, in order to estimate cost-effectiveness of smoking cessation interventions, a lifetime perspective is necessary, taking into account a variety of different costs and effects [12]. Hence, the well-established method to perform cost-effectiveness analyses of smoking cessation interventions involves mathematical modelling of future events as consequences of smoking. Systematic reviews of model-based economic evaluations in smoking cessation analysed different aspects, such as type of model, quality of the model, transferability, and comparison of the results in different studies [12-14]. Berg at al. [13] identified 64 economic evaluations in smoking cessation, and the state-transition Markov model was most frequently used. The majority of the models simulates the lifetime development of morbidity and mortality for smoker vs former smoker using relative risks for four diseases, chronic obstructive pulmonary disease (COPD), coronary heart disease (CHD), stroke, and lung cancer. The authors concluded that existing economic evaluations in smoking cessation vary in quality, resulting mainly from the way in which they selected their populations, measured costs and effects, and assessed the variability and generalisability of their own findings [13]. One of the reasons of the quality issues is that all those studies are based on short-term follow-up (from six months to one year), and they have no possibilities to validate the sustainability of short-term effectiveness in real life; thus, they cannot confirm the reported cost-effectiveness results and policy recommendations. Moreover, the long-term

For peer review only

assumption, such as relapse rate, might change the results of the smoking cessation cost-effectiveness [15].

Our previous economic evaluation of high- and low-intensity programmes (HIT and LIT) for smoking cessation in a dental setting was based on the reported number of quitters measured as point prevalence abstinent (not one puff of smoke during the past seven days prior to 1-year follow-up). The conclusion was that high-intensity treatment support is the preferred option if the decision-makers' willingness-to-pay exceeds €5,100 (50,000 SEK) per QALY. The base-case scenario of the analysis assumed a sustained abstinence for the quitters [16]. The long-term follow-up of the programmes was performed five to eight years later [17]. In this study, we used a unique opportunity to compare cost-effectiveness analyses of a high- and a low-intensity smoking cessation intervention in a dental setting, using data from short-term (1-year) and long-term (5–8 years) follow-up.

We set out to: 1) perform a cost-effectiveness analysis of a high- and a low-intensity smoking cessation programme in a dental setting using long-term (5–8 years) follow-up data and 2) compare the cost-effectiveness results with the previous study based on short-term (1-year) follow-up.

Methods

Summary of the smoking cessation study

In the smoking cessation intervention study [18], between August 2003 and February 2005, 300 adult smokers recruited via direct inquiry or advertising in dental or general health care were offered smoking cessation support performed in a dental setting. Inclusion criteria were daily smokers over 20 years of age, while exclusion criteria were reading difficulties and problems

with Swedish language. The participants were randomly assigned to two interventions; one received high-intensity and one low-intensity treatment support.

The high-intensity smoking cessation treatment, the HIT programme, comprised eight individual sessions, of in total 3.5 hr over a period of 4 months, and was based on behaviour therapy, coaching and pharmacological advice. The low-intensity smoking cessation treatment, the LIT programme, comprised one counselling session, of up to 45 min, introducing a conventional self-help programme running over 8 weeks. Both programmes were free of charge.

The participants answered a baseline questionnaire and a short-term (one year after the planned smoking cessation date) follow-up questionnaire. Demographic characteristics such as gender, age and education level were also collected. The effectiveness of the trial was reported elsewhere [18]. The analysis concluded that the more extensive and expensive HIT programme was more effective and cost-effective, in terms of proportion of smokers who were still smoke-free after one year [16 18]. The long-term follow-up was performed 5–8 years after the planned smoking cessation date. The effectiveness analysis showed that the difference in outcome between the HIT and LIT programmes remained relatively constant and significant in favour of HIT, and that abstinence at 1-year follow-up was a good predictor for long-term abstinence [17]. All analyses were done using the "intention to treat" approach where non-responders were considered as smokers. Mortality and morbidity data for the participants were not collected either by questionnaire or through the registers. The original study, as well as the long-term follow-up, was approved by the ethical committee at Uppsala University (Dnr:Ups 02–457, Dnr: 2010/172).

The mean age of the participants was 49 years, and 78% were women. Short-term follow-up (one year) questionnaire was answered by 84% of the randomised participants (88% for HIT vs 81% for LIT). Fourteen per cent (41 of the 300 participants) reported 6-month continuous

For peer review only

abstinence (not one puff of smoke during the past 6 month); 27 (18%) individuals in HIT vs 14 (9%) in LIT. At long-term follow-up (5–8 years), 241 persons answered the questionnaire (80% for both HIT and LIT). Of those, 24 were sustained abstinent (17 vs 7 for HIT vs LIT) since the planned smoking cessation date. Relapse rate was 26% and 50% for participants reported 6-month continuous abstinence at 1-year follow-up in HIT and LIT respectively, but the difference was not statistically significant**.** Characteristics of the study participants as well as abstinence at the 1-year and at the long-term follow-up are presented in Table 1.

Table 1. Characteristics of the study participants and programme effectiveness at the 1- and 5-8-year follow-up, by treatment intensity.

	HIT N=150	LIT N=150	p-value
Study participants (number)			
Baseline measures	146	148	
12-month follow-up measures	132	122	
Available at long-term follow-up	141	143	
Long-term follow-up measures	121	120	
Participants characteristics			
Gender (number):			
Men	26	32	
Women	115	111	.410
Age at baseline (age):			
mean (SD)	48.7 (9.6)	48.5 (11.0)	
median	48.0	49.0	.825
Education (in years) (number):			
0 - 9	25	36	
10 - 12	60	55	
>=13	52	50	.336
Number of smoked cigarettes/week at baseline:			
mean (SD)	106 (50)	105 (40)	
median	105	105	.794
Intervention effectiveness (number)			
1-year follow-up:			
6-month continuous abstinence	27	14	.034*
5-8 year follow-up:			

For peer review only

Sustained abstinence	17	7	.030*
Relapse rate (%)	26	50	.345

* statistical significant differences at 0.05 level in effectiveness between the programmes

Economic evaluation

Two economic evaluations were performed to obtain the cost-effectiveness of the more costly HIT programme in comparison to LIT:

1) Cost-effectiveness analyses (CEA) based on the number and characteristics of 6-month continuous abstinent participants according to 1-year follow-up, CEA short-term; and

2) Cost-effectiveness analyses based on the number and characteristics of sustained abstinent participants since planned smoking cessation date according to 5–8 years follow-up, CEA long-term.

Both analyses used the same methodology described below.

Economic evaluations were based on the costs to implement the programmes, the number and characteristics of abstinent participants and on a previously constructed Markov model that estimates the future health and cost consequences of smoking cessation. All costs were inflated to reflect 2018 costs according to the Swedish consumer price index [19] and converted into 2018 Euro (€) using the purchasing power parity (PPP) estimates with CCEMG – EPPI-Centre Cost Converter (http://eppi.ioe.ac.uk/costconversion/default.aspx). The cost-effectiveness analyses followed Swedish and international recommendations: costs were calculated from a societal perspective, health effects expressed as quality-adjusted life-years (QALYs), and programmes explicitly compared in an incremental analysis (incremental cost-effectiveness ratio (ICER), with discounting (3% per year) and sensitivity analyses [8 20]. The ICER was calculated by dividing the difference in total costs for the programmes (incremental

For peer review only

cost) by the difference in the health outcomes in QALYs (incremental effect) to provide a ratio of extra cost per extra unit of health effect.

Intervention costs

The intervention costs were collected prospectively by interviewing the three dental hygienists who carried out the patient work as well as the project leader and the project coordinator. The costs were divided into joint costs for the two programmes and programme-specific costs, and undiscounted because of the short 3-year project time. The joint costs were assumed, divided equally between the programmes while the programme-specific costs included staff time for patient work, material, and participant costs. Estimation of the intervention costs has been described in detail previously [16]. Total programme-specific costs amounted to €117,011; €801 per participant for HIT and €27,927; €189 per participant for LIT.

Intervention effectiveness

For CEA short-term, we used 6-month continuous abstinence at 1-year follow-up reported by 41 participants (14 from HIT and 27 from LIT). For CEA long-term, we used sustained abstinence at 5–8 years reported by 24 participants (17 from HIT and 7 from LIT), see Table 1. Both measures were statistically significant different between the treatment programs. In order to generalize the long-term effectiveness of our study, we performed a logistic regression analysis to calculate the probability of sustained abstinence depending on programme (HIT vs LIT), participant's gender and age, see Table 2.

Table 2. Logistic regression analysis of factors associated with sustained abstinence at 5–8 years follow-up

	Coefficient	p-value	OR#	95% CI##

For peer review only

HIT programme	1.001	0.03*	2.72	1.09-6.80
Mail gender	-0.077	0.88	0.93	0.32-2.64
Age	0.005	0.82	1.00	0.96-1.05
Constant	-3,124	0.01	0.04	

* statistical significant at 0.05 level

# - Odds Ratio

- Confidence Interval

The type of the programme (HIT vs LIT) was significantly associated with sustained abstinence while gender and age were not. The regression equation [1] demonstrates dependence between “abstinence” (1 - abstinence, 0 - no abstinence) and “programme” (1 - HIT, 0 - LIT), “gender” (1 - male, 0 - female) and “age” (19-71):

$$abstinence = -3.124 + 1.001 * programme - 0.077 * gender + 0.005 * age \qquad [1]$$

Equation [1] allows us to calculate the probability of long-term abstinence, P_{q}, for a random participant (a random man/woman from a population of interest, smoker between 19 and 71 years old) in respective programme, see equation [2].

$$P_q = EXP\ (abstinence) / \ (1 + EXP\ (abstinence)) \qquad [2]$$

Markov model

A Markov model was used to estimate health consequences and societal costs of smoking cessation, further described in a technical report [21]. The model has been used in similar studies in Sweden [16 22 23], and the updated year 2015 version was used for the current analysis [21]. The model simulates the societal effects of quitting smoking on three disease groups: lung cancer, chronic obstructive pulmonary disease (COPD) and cardiovascular disease, including

For peer review only

coronary heart disease (CHD) and stroke. Even though there are other smoking-related diseases, these conditions cover most of the health problems associated with smoking [24]. The model incorporates the smoking-related disease risks, time-dependent remaining excess disease risks after quitting, the death risks for the specific and for unrelated diseases, as well as the societal costs of the diseases. All disease risks are annual age- and gender-specific excess incidence risks until death or the age of 95. This lifetime horizon was recommended for modelling of smoking cessation interventions [12] because smoking cessation reduces smoking-related health risks gradually during a long period. Notably, the model does not contain the risk for relapse in smoking among the quitters. The societal costs include costs associated with: medical treatment, community care, drugs, informal care and other expenditures for patients and relatives as well as morbidity productivity costs. Health outcomes are expressed in QALYs. The number of QALYs were calculated during healthy years and years spent with a disease, until death or the age of 95. The model and all the parameters are described in detail in a technical report [21] and Appendix 1.

Model simulation were performed according to gender and 5-year age groups. The simulations result in accumulated societal costs and health effects for life-long continuing smokers and quitters at a specific age and gender group, respectively. The differences in societal costs and health effects between smoking statuses at a certain age are then compared outside the model, and constitute the avoided costs and gained health effects from the tobacco quitting for the specified age and gender group

Sensitivity analyses

Extensive sensitivity analyses on parameter values and methodological choices were reported in the model technical report [21]. The model estimates were, in general, insensitive to changes in parameter values, except the most conservative multivariate analysis where the costs were

For peer review only

decreased by 25%, the disease risks by 50%, the death risks by 10%, and the risk fractions after quitting by 0.1. This low cost/low risk analysis led to substantial decreases in cost and QALY differences between quitters and smokers. This sensitivity analysis was applied to compare costs and effects between HIT and LIT, to validate the results of the CEA long-term. To increase the generalizability of the cost-effectiveness results, we have also applied the probabilities of long-term abstinence depending on programme (HIT vs LIT), participant's gender and age on the modelling results. We estimated the avoided social costs and gained QALYs for a random quitter from our sample and then adjusted the results to the probability to quit (Abstinence), calculated in [1]. Cost-effectiveness was estimated for men and women separately.

Further, a probabilistic sensitivity analysis (PSA) was conducted, based on the uncertainty of the difference in sustained abstinent participants in the two programmes. The effectiveness of LIT was fixed at the 7% quit rate, but the HIT quit rate was sampled from the 95% confidence interval (9% – 22%). The PSA was performed by 10,000 runs, using the societal costs avoided and QALY gains for the group with the largest number of quitters, i.e. women aged 40–44 years. The PSA was presented as a cost-effectiveness acceptability curve, which indicates the probability that HIT is cost-effective versus LIT at different values of the willingness-to-pay for a QALY.

Patient and Public Involvement

This research was done without patient involvement. Patients were not invited to comment on the study design and were not consulted to develop patient relevant outcomes or interpret the results. Patients were not invited to contribute to the writing or editing of this document for readability or accuracy.

For peer review only

Results

Model estimates

Model estimates for the CEA short-term and CEA long-term are presented in Table 3 (societal costs and QALYs). The second and third columns in Table 3 present the estimation of avoided societal costs and QALYs gained for a person with respective gender and age, who became sustained abstinent in comparison with a continuing smoker. Using this data, we can estimate the difference in societal cost avoided and QALYs gained by multiplying difference in numbers of 6-month continuous abstinent participants between the treatment programmes (N*) or difference in numbers of sustained abstinent participants since planned smoking cessation date between the treatment programmes (N**) by societal costs avoided and QALYs gained.

For peer review only

Table 3. Model estimates of societal costs avoided and QALYs gained. Costs in **Euro 2018**. 3 % discount rate.

Gender/ Age group	Model estimates		CEA[a]-short HIT[b]	LIT[c]	Difference			CEA[a]-long HIT[b]	LIT[c]	Difference		
	Costs avoided	QALYs[d] gained	N_{Hp}[e]	N_{Lp}[f]	N*	Costs	QALYs[d]	N_{Hs}[g]	N_{Ls}[h]	N**	Costs	QALYs[d]
Women												
20-24	8,142	0.61		1	-1	-8,142	-0.61	na¤	na¤	na¤	na¤	na¤
25-29	8,425	0.65	1		1	8,425	0.65	na¤	na¤	na¤	na¤	na¤
35-39	9,267	0.71	2	2	0			1	1	0		
40-44	8,532	0.71	5		5	42,658	3.55	4		4	34,126	2.84
45-49	6,772	0.66	3	3	0			1	2	-1	-6,772	-0.66
50-54	5,228	0.61	4	3	1	5,228	0.61	1	2	-1	-5,228	-0.61
55-59	4,542	0.43	4	2	2	9,085	0.86	4	1	3	13,627	1.29
60-64	3,336	0. 32	4		4	13,342	1.29	2		2	6,671	0.64
65-69	2,023	0.33		1	-1	-2,023	-0.33	na¤	na¤	na¤	na¤	na¤
Men												
20-24	10,430	0.74	1		1	10,430	0.74	1		1	10,430	0.74
40-44	10,526	1.00	1		1	10,526	1.00	1		1	10,526	1.00
45-49	11,416	0.82	1	1	0			1	1	0		
50-54	11,360	0.78		1	-1	-11,360	-0.78	na¤	na¤	na¤	na¤	na¤
65-69	4,084	0.46	1		1	4,084	0.46	1		1	4,084	0.46
Total			27	14	13	82,253	7.44	17	7	10	67,466	5.71

For peer review only

[a] Cost-effectiveness analysis
[b] High-intensity smoking cessation treatment, the HIT programme
[c] Low-intensity smoking cessation treatment, the LIT programme
[d] Quality-adjusted life-years
[e] N_{Hp} – number of 6-month continuous abstinent participants HIT treatment programme according to 1-year follow-up
[f] N_{Lp} – number of 6-month continuous abstinent participants LIT treatment programme according to 1-year follow-up
[g] N_{Hs} – number of sustained abstinent participants HIT treatment according to 5-8 year follow-up
[h] N_{Ls} – number of sustained abstinent participants LIT treatment according to 5-8 year follow-up
N*– difference in numbers of 6-month continuous abstinent participants between the treatment programmes according to 1-year follow-up
N**– difference in number of sustained abstinent participants between the treatment programmes according to 5-8 year follow-up
na[¤] – 'not applicable'

The CEA short-term indicated that HIT led to additional avoided societal costs of €82,253 and additional 7.44 QALYs compared with LIT. The CEA long-term reported the difference between HIT and LIT as additional avoided societal costs of €67,466 and additional 5.71 QALYs.

Cost-effectiveness analyses

The more costly HIT programme led to a higher number of 6-month continuous abstinent participants at 1-year follow-up (CEA short-term) as well as higher number of sustained abstinent participants at 5–8 year follow-up (CEA long-term), which translates into larger costs avoided and health gains than LIT, see Table 4. However, the difference in intervention costs were not fully balanced by the societal costs avoided, so HIT implied an incremental net cost of about €6,832 in CEA short-term and €21,619 in CEA long-term, compared with LIT. HIT was estimated to lead to more QALYs, so the incremental cost per QALY of HIT compared with LIT amounted to €918 for CEA short-term and €3,786 for CEA long-term, which is considered to be very cost-effective in Sweden [20]. The incremental analysis favours the more costly HIT, if decision-makers are willing to spend at least €4,000/QALY for tobacco cessation programmes.

For peer review only

Table 4. Incremental cost-effectiveness analyses, CEA, of the two smoking cessation treatments, HIT and LIT, for 6-month continuous abstinence at 1-year (CEA short-term), sustained abstinence at 5–8 year follow-up (CEA long-term), and sensitivity analyses for CEA long-term. Societal perspective, in Euro 2018.

				CEA[a] -long, population level, per person	
Intervention costs	CEA[a]-short	CEA[a] -long	CEA[a] -long, sensitivity	Men	Women
HIT[b]	117,011	117,011	117,011	801	801
LIT[c]	27,927	27,927	27,927	189	189
Difference in intervention costs	89,085	89,085	89,085	612	612
Difference in societal costs avoided	82,253	67,466	32,469	779	502
Incremental costs	6,832	21,619	56,616	-167	110
Incremental QALYs[d]	7.44	5.71	4.82	0.0664	0.0462
Incremental cost per QALY[d] (ICER*)	918	3,786	11,746	<0	2,391

* Incremental cost-effectiveness ratio (ICER) is calculated as incremental costs divided by incremental QALYs

[a] – Cost-effectiveness analysis
[b] – High-intensity smoking cessation treatment, the HIT programme
[c] – Low-intensity smoking cessation treatment, the LIT programme
[d] – Quality-adjusted life-years

Sensitivity analyses

The most conservative sensitivity analysis, a multivariate low cost/low risk analysis, was applied to CEA long-term. This analysis led to substantial decreases in avoided social costs and QALY gains for both HIT and LIT. At the same time, the incremental costs increased and incremental QALYs slightly decreased which resulted in higher incremental cost of €11,746 per QALY see Table 4.

The probability of sustained abstinence varies between 0.11 and 0.13 for men and between 0.12 and 0.14 for women in HIT in different ages. The corresponding numbers are 0.4-0.5 for men and 0.5-0.6 for women in LIT. The model estimates for random man and woman were

For peer review only

9,740/0.83 and €7,165 /0.66 for avoided societal costs/QALYs gained. Given the probability of abstinence, the difference in avoided societal costs per person between HIT and LIT was estimated as €779 for men and €502 for women and the correspondent difference in QALYs gained was 0.0664 for men and 0.0462 for women. The incremental cost-effectiveness ration (ICER) was negative for men (HIT was cost saving and entailed positive health outcomes in comparison to LIT) but amounted to €2,391 for women, which is close to our base-case analysis, see Table 4.

At all values of willingness-to-pay for a QALY, including zero, the HIT was more cost-effective than the LIT, see the probabilistic sensitivity analysis on the HIT quit rate in Figure 1.

(insert figure 1 here)

Figure **1.** Probabilistic sensitivity analysis on the effectiveness (proportion of quitters) of high-intensity treatment (HIT) in comparison with low-intensity treatment (LIT), reported as cost-effectiveness acceptability curve, willingness-to-pay per quality-adjusted life-year (QALY), in Euro 2018.

Discussion

Main results

In this study, we performed a cost-effectiveness analysis using the long-term follow-up data from a RCT of a high- and a low-intensity treatment programme (HIT and LIT) for smoking cessation in a dental setting. We also validated the cost-effectiveness results of the previous study based on short-term follow-up [16]. HIT was more effective in getting participants to quit smoking and to keep sustained abstinent, resulted in higher societal costs avoided and more QALYs gained among both men and women, compared with LIT and thus can be considered cost-effective**.** The incremental cost-effectiveness ratios (ICERs) were €918 and €3,786 using short- and long-term effectiveness, respectively, which are below the Swedish willingness-to-pay threshold of €50,000 per QALY [25], thus, indicating that the resource intensive HIT was

cost-effective compared to the less resource demanding LIT. The results also confirm the conclusions of the previous cost-effectiveness analysis based on short-term follow-up data and suggest its sustainability**.** We would recommend the use of the HIT programme as a cost-effective option for smoking cessation.

Notably, the usage of both the HIT and LIT programmes is not limited to dental settings and can be implemented in other healthcare sectors and delivered by trained nurses instead of dental hygienists. Since the salaries of registered nurses and dental hygienists are comparable, the conclusion of high cost-effectiveness of the HIT programme remains.

However, although HIT was shown to be cost-effective in comparison with LIT, the sensitivity analysis using the probability of abstinence suggested that HIT dominated over LIT for men (saved societal costs and generated more QALYs). In our sample the majority of study participants were women, that is why the results of the sensitivity analysis for women was very close to our base-case analysis.

Strength and limitations

The majority of cost-effectiveness analyses on smoking cessation use one year quit rates in their models; however, it is not uncommon that 6-month quit rates are used [12 26]. The question of how much we can trust the overall conclusions of such analyses always remains, because we do not know for sure what happens subsequently. To our knowledge, this is the first study that utilises a unique possibility to compare a previously conducted cost-effectiveness analyses based on 6-month continuous abstinent participants at 1-year follow-up with a new evaluation, based on sustained abstinence since the planned smoking cessation date up to 5–8 years. We had the possibility to compare the results based on 6-month continuous abstinence (when some time-dependent excess disease risks remained for the first years after quitting)

and sustained abstinence for 5–8 years (when the smoking-related excess disease risks had been reduced). A higher proportion of sustained abstinent participants in HIT compared to LIT contributed to a low ICER for the long-term cost-effectiveness analyses.

The effects of smoking cessation are certainly underestimated in the model estimates since only three disease groups including lung cancer are modelled and no effects of passive smoking are included, but smoking is causally related to at least 15 other types of cancer [33]. In addition, quitting smoking reduced the rate of incidence diabetes to that of non-smokers after five years in women and after 10 years in men [27]. The model does not include the health problems related to passive smoking, such as risk of CHDs in offspring [28] and increase in risk for breast cancer [29]. That makes our estimations more conservative with respect to cost savings and QALYs, although these three diseases do account for over 80% of morbidity (and mortality) associated with smoking and are frequently used in similar studies [15 30]. Another limitation is that the model does not include the relapse rate among the quitters. This tends to overestimate the health and cost consequences of the tobacco quitting based on short-term outcomes, because the relapse rate is presumably higher among the short-term quitters. On the other hand, the relapse rate might be negligibly low among individuals that quit smoking 5-8 years ago and thus not important for the modelling results. Additionally, as mentioned in our previous study [16], the Markov model indicates considerably lower smoking-related disease risks for women reported by large epidemiological studies (see model technical report for details) [21], and thus lower cost savings and health gains from tobacco cessation for women than for men. Finally, the intervention costs for the RCT study calculation was based on the trial protocol and might be overestimated in comparison with routine practice; however, in the ICER, those extra costs were divided equally between the programmes, and thus disregarded.

For peer review only

Comparison with other studies

We could not find any cost-effectiveness analyses based on more than 1-year follow-up, and therefore we compared our results with other studies estimating cost-effectiveness of interventions with different level of intensity using 6- or 12-month follow-up. Thus, a cost-effectiveness analysis of high intensity multiple contests and low intensity enhanced contest of a Quit-and-Win programme reported that high intensity Quit-and-Win leads to an average gain of 0.03 QALYs and was cost-saving, in comparison with lower intensity [9]. Another study presented a cost-effectiveness analysis of three smoking cessation interventions with different intensity levels: Standard Care (SC) (brief advice to quit, nicotine replacement therapy and self-help written materials), Enhanced Care (EC) (SC plus cell phone-delivered messaging) and Intensive Care (IC) (EC plus cell phone-delivered counselling) [10]. The overall conclusion was that the higher intensive intervention (IC) was the most cost-effective strategy both for men and women, which is in line with our results. Additionally, a cost-effectiveness analysis of two smoking cessation approaches for cancer patients was presented in a study from Canada [11]. The basic programme consisted of screening for tobacco use, advice and referral, whereas the best practice programme included a basic programme and pharmacological therapy, counselling and follow-up. The incremental cost-effectiveness ratio of the best practice programme compared to the basic programme was $3,367 per QALY gained for men, and $2,050 per QALY gained for women. These results are very similar to our findings. In our previous study [16], based on the same RCT and 1-year follow-up, a higher ICER of €9,900/QALY and €5,500 /QALY was calculated for point prevalence and continuous abstinence respectively, but the overall conclusion confirmed the cost-effectiveness of HIT at a willingness-to-pay of €10,000.

For peer review only

Conclusions

In conclusion, the more costly HIT smoking cessation programme is an economically attractive option when compared to the LIT programme over a broad range of assumptions, using shot- and long-term outcomes. Cost-effectiveness analysis favours the more costly HIT if decision-makers are willing to spend at least €4,000/QALY for tobacco cessation treatment. These findings can support and guide implementation of smoking cessation programmes.

Contributors

IF and EN conceived and designed the study and drafted the manuscript. Modelling and economic evaluation was carried out by IF and PJ. AR, ÅT and EN were responsible for clinical evaluation of the smoking cessation study. All the authors (IF, AR, ÅT, PJ and EN) contributed to the writing process and have approved the final manuscript.

Funding

This study was funded by grants from the County Council of Västmanland, Sweden (LTV 3999) and Swedish Research Council for Health, Working Life and Welfare (FORTE), grant number 2014-1399.

Competing interests

None declared.

Ethics approval

The Ethical Committee Uppsala University gave clearance for the smoking cessation study Dnr: Ups 02-457.

For peer review only

Data sharing statement

Data is available from corresponding author (IF) on reasonable request.

References

1. [Folkhälsomyndigheten] PHAoS. Public Health Agency of Sweden [Folkhälsomyndigheten] Stockholm2018 [Available from: https://www.folkhalsomyndigheten.se/the-public-health-agency-of-sweden/living-conditions-and-lifestyle/alcohol-narcotics-doping-tobacco-and-gambling/tobacco/ accessed 2018-05-05 2018.
2. Institute for Health Metrics and Evaluation. Country Profiles: Sweden [Available from: http://www.healthdata.org/sweden accessed Nov 5 2015.
3. Andersson E, Toresson Grip E, Norrlid H, et al. Samhällskostnaden för rökningsrelaterad sjuklighet i Sverige [The social cost of smoking-related illness in Sweden]. In: IHE, ed. IHE Rapport 2017:4. Lund, Sweden, 2017.
4. Thomas JL, Luo X, Bengtson J, et al. Enhancing Quit & Win contests to improve cessation among college smokers: a randomized clinical trial. *Addiction* 2016;111(2):331-9. doi: 10.1111/add.13144 [published Online First: 2016/01/16]
5. Vidrine DJ, Fletcher FE, Danysh HE, et al. A randomized controlled trial to assess the efficacy of an interactive mobile messaging intervention for underserved smokers: Project ACTION. *BMC public health* 2012;12:696. doi: 10.1186/1471-2458-12-696 [published Online First: 2012/08/28]
6. McFarland A. Economic evaluation of interventions in health care. *Nursing standard* 2014;29(10):49-58. doi: 10.7748/ns.29.10.49.e9315
7. Anderson R. Systematic reviews of economic evaluations: utility or futility? *Health economics* 2010;19(3):350-64. doi: 10.1002/hec.1486
8. Drummond M, Sculpher M, Torrance G, et al. Methods for the economic evaluation of health care programmes. 3rd edn ed. Oxford: Oxford University Press 2005.
9. Popp J, Nyman JA, Luo X, et al. Cost-effectiveness of enhancing a Quit-and-Win smoking cessation program for college students. *The European journal of health economics : HEPAC : health economics in prevention and care* 2018 doi: 10.1007/s10198-018-0977-z [published Online First: 2018/04/25]
10. Daly AT, Deshmukh AA, Vidrine DJ, et al. Cost-effectiveness analysis of smoking cessation interventions using cell phones in a low-income population. *Tobacco control* 2018 doi: 10.1136/tobaccocontrol-2017-054229 [published Online First: 2018/06/11]
11. Djalalov S, Masucci L, Isaranuwatchai W, et al. Economic evaluation of smoking cessation in Ontario's regional cancer programs. *Cancer medicine* 2018 doi: 10.1002/cam4.1495 [published Online First: 2018/07/19]
12. Bolin K. Economic evaluation of smoking-cessation therapies: a critical and systematic review of simulation models. *PharmacoEconomics* 2012;30(7):551-64. doi: 10.2165/11590120-000000000-00000
13. Berg ML, Cheung KL, Hiligsmann M, et al. Model-based economic evaluations in smoking cessation and their transferability to new contexts: a systematic review. *Addiction* 2017;112(6):946-67. doi: 10.1111/add.13748
14. Kirsch F. A systematic review of quality and cost-effectiveness derived from Markov models evaluating smoking cessation interventions in patients with chronic obstructive pulmonary disease. *Expert review of pharmacoeconomics & outcomes research* 2015;15(2):301-16. doi: 10.1586/14737167.2015.1001976 [published Online First: 2015/01/21]

For peer review only

15. Pennington B, Filby A, Owen L, et al. Smoking Cessation: A Comparison of Two Model Structures. *PharmacoEconomics* 2018 doi: 10.1007/s40273-018-0657-y [published Online First: 2018/05/08]
16. Nohlert E, Helgason AR, Tillgren P, et al. Comparison of the cost-effectiveness of a high- and a low-intensity smoking cessation intervention in Sweden: a randomized trial. *Nicotine & tobacco research : official journal of the Society for Research on Nicotine and Tobacco* 2013;15(9):1519-27. doi: 10.1093/ntr/ntt009
17. Nohlert E, Ohrvik J, Tegelberg A, et al. Long-term follow-up of a high- and a low-intensity smoking cessation intervention in a dental setting--a randomized trial. *BMC public health* 2013;13:592. doi: 10.1186/1471-2458-13-592 [published Online First: 2013/06/20]
18. Nohlert E, Tegelberg A, Tillgren P, et al. Comparison of a high and a low intensity smoking cessation intervention in a dentistry setting in Sweden: a randomized trial. *BMC public health* 2009;9:121. doi: 10.1186/1471-2458-9-121 [published Online First: 2009/05/02]
19. Statistics Sweden. Konsumentprisindex [Available from: http://www.scb.se/pr0101/ accessed April 14, 2016.
20. Pharmaceutical Benefits Board. General guidelines for economic evaluations from the Pharmaceutical Benefits Board, 2003.
21. Johansson. A model for economic evaluations of smoking cessation interventions – technical report. Model version 3. Folkhälsa & Ekonomi (Public Health & Economics), 2015.
22. Johansson PM, Tillgren PE, Guldbrandsson KA, et al. A model for cost-effectiveness analyses of smoking cessation interventions applied to a Quit-and-Win contest for mothers of small children. *Scandinavian journal of public health* 2005;33(5):343-52.
23. Virtanen SE, Galanti MR, Johansson PM, et al. Economic evaluation of a brief counselling for smoking cessation in dentistry: a case study comparing two health economic models. *BMJ open* 2017;7(7):e016375. doi: 10.1136/bmjopen-2017-016375 [published Online First: 2017/07/22]
24. Gunning-Schepers LJ . The health benefits of prevention: a simulation approach. *Health policy and planning* 1989(12):1-256.
25. Svensson M, Nilsson FO, Arnberg K. Reimbursement Decisions for Pharmaceuticals in Sweden: The Impact of Disease Severity and Cost Effectiveness. *PharmacoEconomics* 2015;33(11):1229-36. doi: 10.1007/s40273-015-0307-6 [published Online First: 2015/06/22]
26. Chen YF, Madan J, Welton N, et al. Effectiveness and cost-effectiveness of computer and other electronic aids for smoking cessation: a systematic review and network meta-analysis. *Health technology assessment* 2012;16(38):1-205, iii-v. doi: 10.3310/hta16380 [published Online First: 2012/10/11]
27. Will JC, Galuska DA, Ford ES, et al. Cigarette smoking and diabetes mellitus: evidence of a positive association from a large prospective cohort study. *International journal of epidemiology* 2001;30(3):540-6. doi: 10.1093/ije/30.3.540 [published Online First: 2001/06/21]
28. Zhao L, Chen L, Yang T, et al. Parental smoking and the risk of congenital heart defects in offspring: An updated meta-analysis of observational studies. *European journal of preventive cardiology* 2019:2047487319831367. doi: 10.1177/2047487319831367 [published Online First: 2019/03/25]
29. Macacu A, Autier P, Boniol M, et al. Active and passive smoking and risk of breast cancer: a meta-analysis. *Breast cancer research and treatment* 2015;154(2):213-24. doi: 10.1007/s10549-015-3628-4 [published Online First: 2015/11/08]
30. Hurley SF, Matthews JP. The Quit Benefits Model: a Markov model for assessing the health benefits and health care cost savings of quitting smoking. *Cost effectiveness and resource allocation : C/E* 2007;5:2. doi: 10.1186/1478-7547-5-2

For peer review only

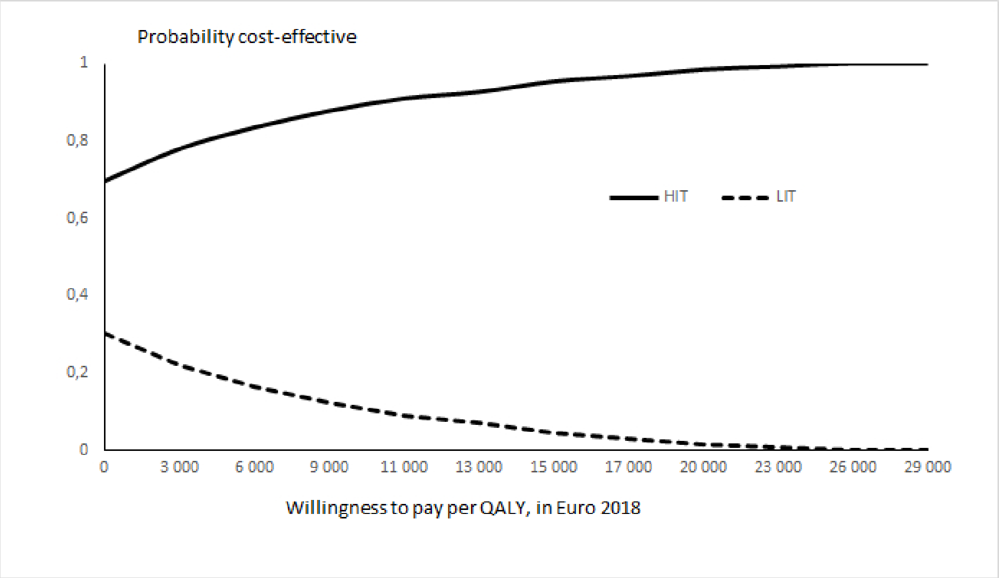

Figure 1. Probabilistic sensitivity analysis on the effectiveness (proportion of quitters) of high-intensity treatment (HIT) in comparison with low-intensity treatment (LIT), reported as cost-effectiveness acceptability curve, willingness-to-pay per quality-adjusted life-year (QALY), in Euro 2018.

A model for economic evaluations of smoking cessation interventions – technical report

Version 3 year 2015

Pia Johansson

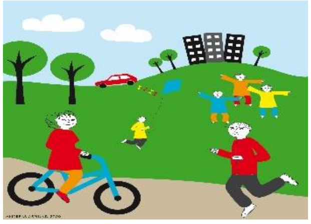

Folkhälsa&Ekonomi
PublicHealth&Economics

Stockholm 2015

Pia.johansson@folkhälsaetekonomi.se
Pia.johansson@publichealtheteconomics.se

This report is available at PublicHealth&Economics.se:
http://www.publichealtheconomics.se

For peer review only

Content

TABLES **3**

INTRODUCTION **4**

METHOD **5**

THE DISEASES 5
THE MODEL 5

MATERIAL **7**

THE RISKS 7
Disease risks *7*
Death risks *10*
Changes in risk after quitting smoking *12*
THE SOCIETAL COSTS 12
Medical treatment costs *13*
Institutional care and technical aids costs *13*
Pharmaceutical costs *14*
Informal care and other patient and relatives' costs *14*
Productivity costs *15*
THE HEALTH EFFECTS 15
Life years lost *15*
QALYs *15*
SENSITIVITY ANALYSES 16
Univariate analyses *17*
Multivariate analyses *17*
Analyses on methodological issues *17*
Probabilistic analysis *17*

RESULTS **18**

THE MODEL ESTIMATES 18
SELECTED MODEL OUTCOMES 20
SENSITIVITY ANALYSES 22

DISCUSSION: MODEL VALIDITY **25**

THE STRUCTURE OF THE MODEL 25
THE INPUTS OF THE MODEL 26
THE RESULTS OF THE MODEL 28
THE VALUE OF THE MODEL TO THE DECISION-MAKER 29
THE UNCERTAINTY 30
CHECKING FOR TECHNICAL ERRORS 30
CONCLUSIONS 31

REFERENCES **32**

For peer review only

For peer review only - http://bmjopen.bmj.com/site/about/guidelines.xhtml

Tables

Table 1. The model diseases, with ICD-10 codes. 5
Figure 1. State-transition diagram 6
Table 2. Risks COPD. 7
Table 3. Risks lung cancer. 8
Table 4. Risks CHD and stroke. 8
Table 5. The annual risks of CHD. 9
Table 6. The annual risks of stroke. 9
Table 7. Distribution of diseases within CHD. 9
Table 8. Death risk lung cancer. 10
Table 9. Death risk AMI, 1st year. 10
Table 10. Death risk stroke, 1st year. 11
Table 11. Death risk CHF. 11
Table 12. Death risks, unrelated. 11
Table 13. Medical treatment costs. SEK 2014. 12
Table 14. Costs for institutional care and technical aids. SEK 2014. 13
Table 15. Pharmaceutical costs. SEK 2014. 14
Table 16. Informal care and other patient and relatives' costs. SEK 2014. 14
Table 17. Productivity costs, morbidity. SEK 2014. 15
Table 18. Average Swedish population QoL weights. 16
Table 19. QoL weights and QoL decrements due to disease. 16
Table 20. QALYs, until age 95 years, discounted 3%. 18
Table 21. Life years lost (YLS), before age 95 years. Discounted 3%. 19
Table 22. Life years lost (YLS), before age 95 years. Undiscounted. 19
Table 23. Societal costs. In SEK 2014 and discounted 3%. 20
Figure 2. The disease outcome, number of diseased and dead per 10 000 for smokers and quitters, women aged 50 years. 20
Figure 3. The disease outcome, number of diseased and dead per 10 000 for smokers and quitters, men aged 50 years. 21
Table 24. Societal cost savings, in SEK 2014. Women aged 50 years. 21
Table 25. Societal cost savings, in SEK 2014. Men aged 50 years. 22
Figure 4. Sensitivity analyses on societal cost and QALY differences between smokers and quitters, women aged 50 years. 22
Figure 5. Sensitivity analyses on societal cost and QALY differences between smokers and quitters, men aged 50 years. 23
Figure 6. The cost-effectiveness plane with resultat från bootstrap, women aged 50 years. 23
Figure 7. The cost-effectiveness plane with resultat from bootstrap, men aged 50 years. 24

For peer review only

Introduction

This is a technical report on an updated version of a model, originally developed in year 2004 (Johansson, 2004), to enable systematic cost-effectiveness analyses of tobacco cessation interventions in Sweden. It aims to follow international and Swedish recommendations of cost-effectiveness analyses in health and medicine. The model holds a societal perspective, aiming to incorporate available disease-specific costs for all sectors of Swedish society. The updated model contains more recent data on societal costs, disease and death risks, and quality-of life-estimates, to enable estimates that reflects current Swedish conditions.

The model simulates the lifetime societal effects of quitting smoking on three diseases: lung cancer, chronic obstructive pulmonary disease (COPD), and cardiovascular disease (CVD) including coronary heart disease (CHD) and stroke. The model incorporates the smoking-related disease risks, the remaining disease risks after tobacco quitting, the death risks in the diseases and unrelated diseases, as well as the societal effects of the diseases. The societal effects include medical treatment costs, costs for institutional care, drug costs, costs for informal care and other costs for patients and relatives, and morbidity productivity costs, as well as loss of life-years and quality-adjusted life-years (QALYs).

This technical report contains a description of the model structure, of all the data sources used and of the assumptions made. For validation purposes, it also reports model estimates for some selected age-groups and more detailed outcomes and sensitivity analyses for one age-group, men and women aged 50 years at the start of the simulations. To investigate model uncertainty, univariate and multivariate sensitivity analyses are reported, as well as a probabilistic analysis. The model validity is discussed in the final section of the report.

For peer review only - http://bmjopen.bmj.com/site/about/guidelines.xhtml

Method

The diseases

The model incorporates the three most smoking-related diseases: lung cancer, chronic obstructive pulmonary disease (COPD), and cardiovascular disease (CVD) including coronary heart disease (CHD) and stroke, see table 1. The model is restricted to the effects on the individual smoker/quitter, thus not incorporating any side-effects on other people.

The model

The stochastic model simulates the societal effects of smoking cessation on three smoking-related diseases. It is constructed as a Markov-cycle tree model appropriate for microsimulations.

The Markov model is a health state-transition model (Sonnenberg & Beck, 1993; Briggs & Sculpher, 1998) using probabilities for transitions between health states. These probabilities are the age- and gender-specific disease risks, conditional on smoking status and years since quitting, and age-, gender- and disease-specific death risks. The states are mutually exclusive and collectively exhaustive, and transitions between disease states are not allowed. The only exits from disease states are death, in the disease in question or in unrelated diseases, except for 5-year survivors in lung cancer which are assumed to recover to complete health. All other disease states are assumed to last life-long. See figure 1 for the state-transition diagram.

The Markov stages are one year-long, with no half-cycle correction. The starting point is the state healthy. The model covers the ages 15 to 95 years. The Markov-cycle tree has been created in Treeage Pro (Treeage Inc., 2015).

For peer review only

Table 1. The model diseases, with ICD-10 codes.

Disease	ICD-10
Lung cancer	C34
COPD	J44
Stroke	I61 I63 I64
Coronary heart disease, CHD:	
Acute myocardial infarction, AMI	I21 I22 I23
Congestive health failure, CHF	I50.
Ischemic heart disease, IHD	I20 I24 I25
Sudden death	I46.1

For peer review only - http://bmjopen.bmj.com/site/about/guidelines.xhtml

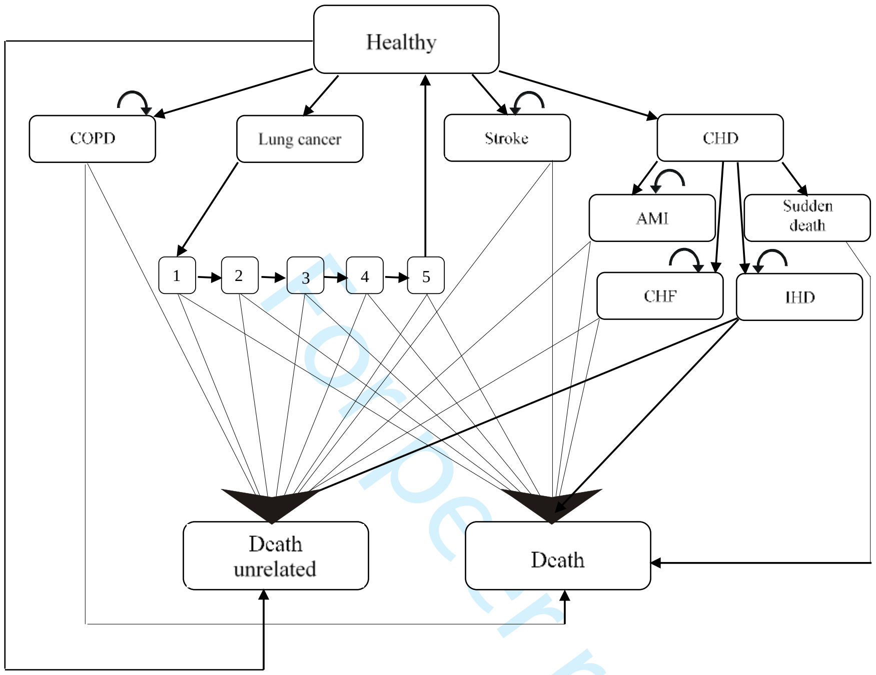

Figure 1. State-transition diagram

The model is set up with two reward sets; costs and effects. The incremental rewards are accumulated during time spent in the health states. The transitional rewards lost life years and some costs are recorded at transitions between healthy and disease state, and disease state and death.

The Markov-cycle tree is run as a microsimulation with 10 000 repetitions. The simulation ends at death or age 95 years. The model is run separately for age and gender groups. The result of each simulation is expected value, with accompanying distributions. The two simulations, the continuing smoker and the quitter, are compared outside the model. The results are presented as expected value per individual, specific for gender, age and smoking status.

For peer review only - http://bmjopen.bmj.com/site/about/guidelines.xhtml

Material

The model is based on principles for cost-effectiveness analysis in health and medicine (Gold et al, 1996; Drummond et al, 2005) and Swedish methodological recommendations (TLV, 2004). The model holds the societal perspective, aiming to incorporate disease-specific costs for all sectors of Swedish society.

The model uses Swedish register data and secondary data from previously published scientific articles. The secondary data was found through searches in the database MEDLINE and the reference lists of retrieved articles, choosing the data that is considered most relevant to present-day Swedish circumstances and the target group. No meta-analysis nor other synthesis of data was performed.

All costs are expressed in year 2014 SEK (USD 1=SEK 6.86; Euro 1=SEK 9.10), converted if necessary by the Swedish CPI (consumer price index). The annual discount rate is 3% for both costs and health effects.

The risks

Disease risks

All disease risks are annual age- and gender-specific excess incidence risk until the age of 95 years, see tables 2 to 5.

The COPD disease risk is taken from the Swedish population-based study Obstructive Lung Disease in Northern Sweden (OLIN), which was started in year 1985 (Lundbäck et al, 1991). The risk is the reported average excess seven-year incidence among smokers in three age groups, of which the youngest was 45 years at baseline, see table 2. COPD was defined according to the spirometer GOLD definition.

Table 2. Risks COPD.

	men & women	source
Disease risk		
Risk until age 45	0%	Lindberg et al, 2006
Excess annual risk for smokers, from age 46	1.6%	
Effect of quitting		
Risk fraction for quitters, years since quitting:		Inspired by Surgeon General, 1990
0-5	1	
6-15	0.5	
16-24	0.3	
>25	0.1	
Death risk		
Excess risk among diseased, as fraction of age-specific general death risk, by age:		Estimated from Lundbäck et al, 2009 Statistics Sweden, database
<58 years	1	
58-70 years	5	
>70 years	1	

For peer review only - http://bmjopen.bmj.com/site/about/guidelines.xhtml

For peer review only

Table 3. Risks lung cancer.

	men	women	source
Death risk			
Accumulated death risk until age 75			
Smokers	16.7%	10.4%	Peto et al, 2000
Non-smokers	0.4%	0.4%	
Risk for ages <40	0	0	Assumed, based on Peto et al, 2000
Smokers accumulated excess death risk until age 95	37.2%	23.1%	Interpolated, based on Peto et al, 2000
Age-adjusted conditional death risk	see table 8		
Disease risk			
Smokers accumulated excess disease risk until age 95	42.0%	26.3%	After interpolation, based on Peto et al, 2000 and Holm et al, 1995
Effect of quitting			
Risk fraction for quitters, years since quitting:			Peto et al, 2000
<10	0.66	0.69	
10-19	0.42	0.21	
20-29	0.18	0.05	
30-35	0.08	0	
>36	0	0	

The lung cancer disease risk is estimated from reports on lung cancer deaths until age 75 for smokers (15-24 cigarettes/day) and non-smokers, see table 3. The annual excess death risk is estimated by a quadratic function of the accumulated risk until age 75 years. The lung cancer death risk is assumed 0 until the age of 40 years, and assumed constant between ages 75 and 95. The disease risk is obtained by adjusting the annual death risk by the annual crude survival rate of lung cancer in Sweden for a similar time period as the Peto data, from Holm et al (1995).

Table 4. Risks CHD and stroke.

	men & women	source
Disease risk	Framingham, see tables 5-7	
Effect of quitting		
Risk fraction for quitters, years since quitting:		Surgeon General, 1990
on CHD:		
1	0.5	
>15	0	
on stroke:		
>10	0	
Death risk		
AMI, 1st year	see table 9	
Stroke, 1st year	see table 10	
CHF	see table 11	
Risks as fraction of age- and gender-specific general death risk:		Statistics Sweden
AMI, 2nd and following years, age 15-93 years	3	Henriksson et al, 2014
AMI, 2nd and following years, age >93 years	2	Assumed
Stroke, 2nd and following years, age 15-93 years	3	Henriksson et al, 2014
Stroke, 2nd and following years, age >93 years	2	Assumed
IHD, 1st year	2.5	Granström et al, 2012
IHD, 2nd and following years	2.15	Granström et al, 2012

For peer review only - http://bmjopen.bmj.com/site/about/guidelines.xhtml

Table 5. The annual risks of CHD.

μ_{chd} =5.5305+28.4441*Sex-1.479*Ln(Age)-14.4588*Ln(Age)*Sex+1.8515*(Ln(Age))²*Sex-0.9119*Ln(SBP)-0.2767*Smok-0.7181*Ln(Chol/HDL)-0.1759*Diabetes-0.1999*Diabetes*Sex

P_{chd} =1-Exp(-Exp((- μ_{chd})/ Exp(0.9145-0.2784* μ_{chd})))

Source: Caro et al, 2007; Anderson et al, 1991

Table 6. The annual risks of stroke.

μ_{str} =26.5116+0.2019*Sex-2.3741*Ln(Age)-2.4643*Ln(SBP)-0.3914*Smok-0.0229*Ln(Chol/HDL)-0.3087*Diabetes-0.2627*Diabetes*Sex

P_{str} = 1-Exp(-Exp((- μ_{str})/ Exp(-0.04312* μ_{str})))

Source: Caro et al, 2007; Anderson et al, 1991

The CHD and stroke disease risk estimates are based on the Framingham CVD risk function, see table 4 and tables 5-6. As the Framingham CHD risk function only calculates CHD events, the division of these events into the particular diseases are based on recent Swedish register data, see table 7. To avoid over-estimation of risks, the risk factors for CHD and stroke are evaluated at minimal-risk levels; 120 mmHg for systolic blood pressure (SBP), HDL-cholesterol (HDL) at 1.5 and cholesterol (Chol) at 4. Diabetes is set at 0, while the variable smoking (smok) is set at 1 for the smokers.

Table 7. Distribution of diseases within CHD.

	Age < 65 years		Age >65 years	
	men	women	men	women
AMI	0.42	0.40	0.31	0.31
IHD	0.40	0.39	0.21	0.29
CHF	0.16	0.19	0.46	0.38
Sudden death	0.02	0.02	0.02	0.02

Source: Swedish National Board of Health and Welfare, Statistics database, Diagnoses in inpatient care from the Hospital Discharge Register, year 2013.

For peer review only - http://bmjopen.bmj.com/site/about/guidelines.xhtml

Table 8. Death risk lung cancer.

Age group	Years since diagnosis 1	2	3	4	5
0-54	0.550	0.172	0.034	0.034	0.034
55-74	0.610	0.168	0.030	0.030	0.030
75-95	0.743	0.120	0.021	0.021	0.021

Source Based on Talbäck et al, 2004

Death risks

All death risks are age-and gender disease-specific conditional risks; in some cases estimated as fractions of the general population age- and gender-specific mortality risk, see tables 2 to 4, and in some cases based on Swedish register data, see tables 8 to 11.

The COPD death risk is estimated from the study Obstructive Lung Disease in Northern Sweden (OLIN), which reported the 20-year mortality in three age groups. Comparison with the general age-specific mortality risks revealed no excess risk of death among those younger than 58 years and older than 70 years, but a considerable increased risk among those aged 58-70 years at follow-up. The excess risk was estimated at on average around 5 times the age- and gender-specific general population death risk, see table 2.

The lung cancer death risk is based on survival data from the Swedish National Cancer Registry, see table 8. The death risks for year 3 and 4 after diagnosis are estimated by linear interpolation between years 2 to 5. Lung cancer survivors at 5 years are assumed recovered, and returned to the health state healthy.

The death risks from CHD and stroke are taken from Swedish registers, see tables 9 to 11, or published scientific reports, see table 5. The death risks for AMI, stroke and IHD are divided into risks the first year after the first event and the second and following years after first event.

Table 9. Death risk AMI, 1st year.

Age group	men	women
20-49	0.077	0.077
50-64	0.137	0.101
65-69	0.159	0.149
70-74	0.172	0.141
75-79	0.206	0.191
80-84	0.255	0.224
>84	0.327	0.331

Source: Swedish National Board of Health and Welfare, The Swedish AMI Statistics, year 2013

For peer review only - http://bmjopen.bmj.com/site/about/guidelines.xhtml

Table 10. Death risk stroke, 1st year.

Age group	men	women
20-49	0.031	0.038
50-54	0.059	0.051
55-59	0.044	0.064
60-64	0.046	0.061
65-69	0.062	0.066
70-74	0.077	0.085
75-79	0.097	0.109
80-84	0.148	0.157
>84	0.216	0.257

Source: Swedish National Board of Health and Welfare. The Swedish Stroke Statistics, year 2013

Table 11. Death risk CHF.

Age group	men	women
15-49	0	0
50-69	0.057	0.015
70-84	0.245	0.162
>84	0.340	0.281

Source: Swedish National Heart Failure Register, year 2012

The model also incorporates the possibility of dying in unrelated diseases. The death risk in the health state Healthy is the average 5-year age group- and gender-specific risk adjusted for all model disease deaths, the last column in table 12. In disease health states, the risk of dying in unrelated disease is the average 5-year age group- and gender-specific

Table 12. Death risks, unrelated.

Age Group	Not COPD		Not Lung cancer		Not AMI		Not CHF		Not IHD		Not Sudden death		Not Stroke		Not model disease	
	m	w	m	w	m	w	m	w	m	w	m	w	m	w	m	w
<39	0.001	0.000	0.001	0.000	0.001	0.000	0.001	0.000	0.001	0.000	0.001	0.000	0.001	0.000	0.001	0.000
40-44	0.001	0.001	0.001	0.001	0.001	0.001	0.001	0.001	0.001	0.001	0.001	0.001	0.001	0.001	0.001	0.001
45-49	0.002	0.001	0.002	0.001	0.002	0.001	0.002	0.001	0.002	0.001	0.002	0.001	0.002	0.001	0.002	0.001
50-54	0.003	0.002	0.003	0.002	0.003	0.002	0.003	0.002	0.003	0.002	0.003	0.002	0.003	0.002	0.003	0.002
55-59	0.005	0.003	0.004	0.003	0.004	0.003	0.005	0.003	0.004	0.003	0.005	0.003	0.005	0.003	0.005	0.003
60-64	0.008	0.005	0.007	0.005	0.007	0.005	0.008	0.005	0.007	0.005	0.008	0.005	0.008	0.005	0.008	0.005
65-69	0.013	0.008	0.012	0.008	0.012	0.009	0.013	0.009	0.012	0.009	0.013	0.009	0.013	0.009	0.013	0.008
70-74	0.021	0.013	0.020	0.013	0.019	0.014	0.021	0.014	0.020	0.013	0.021	0.014	0.020	0.014	0.021	0.013
75-79	0.037	0.023	0.036	0.023	0.035	0.024	0.037	0.024	0.035	0.023	0.038	0.024	0.036	0.023	0.037	0.023
>79	0.068	0.047	0.068	0.047	0.065	0.047	0.068	0.047	0.065	0.046	0.071	0.048	0.068	0.046	0.068	0.047

m=men, w=women

Source: Swedish National Board of Health and Welfare. The Swedish National Causes of Death Register, year 2014

For peer review only

risk adjusted for the deaths in each respective disease. For ages below 39 years the risk in the age group 35-39 years is used, and for ages 80-84 years the risk >79 years. For ages above 84 years, the general population age-and gender specific death risk is used for the unrelated death risk. As the lung cancer death risks are so high, the unrelated death risks for lung cancer among individuals aged above 84 years had to be adjusted, by deducting 0.05. For those aged below 85 years, the age- and gender-specific general population risk of death is only used for calculating some disease-specific death risks, see tables 2 and 4. The risk is taken from the Swedish national mortality statistics for the year 2014 (Statistics Sweden, 2015).

Changes in risk after quitting smoking

The excess disease risks for smokers are not eliminated immediately after quitting smoking. This "lead time" is 36 years for lung cancer, 16 years for CHD, and 11 years for stroke, while for COPD some excess risk remain life-long, see heading effect of quitting in tables 2 to 4. The disease risks after quitting are constructed by adjusting the smokers' risks by the remaining risk. The remaining risk is modelled as fractions of risk, given the number of years since quitting. The annual remaining risks are estimated by linear interpolation. The effects on the risk for CHD and stroke are modelled on the dummy variable smoking, adjusting the value of 1 by the remaining risk fraction.

The societal costs

The model is reflecting the societal perspective, including disease-related costs for all sectors of the Swedish society. The costs included are medical treatment costs, costs for institutional care and technical aids, pharmaceutical costs, informal care and other patient and relatives' costs, and morbidity productivity costs.

Most of the data on societal costs are taken from Swedish studies published during the 2010s. Data reported as distributions, i.e. with the Gamma parameters for costs, or bootstrapped 95 percent confidence interval were preferred and used in the model to

Table 13. Medical treatment costs. SEK 2014.

	mean	95% confidence interval	distribution	source	comment
Lung cancer	76 096	-	-	KPP register, SALAR 2015	Only inpatient care
COPD	10 120	6 120 - 14 920	-	Jansson et al, 2013	Moderate COPD
AMI year 1	171 660	-	Gamma 106;1622	Henriksson et al, 2014	
AMI year 2+	45 740	-	Gamma 17;2698	Henriksson et al, 2014	
CHF	33 850	-	-	Agvall et al, 2005	
IHD	51 610	-	-	Mourad et al, 2013	Angina pectoris
Stroke year 1	142 280	-	Gamma 114;1244	Henriksson et al, 2014	
Stroke year 2+	38 450	-	Gamma 48;800	Henriksson et al, 2014	

For peer review only - http://bmjopen.bmj.com/site/about/guidelines.xhtml

enable stochastic estimation. If data was reported as mean and standard deviation, the Gamma distribution was simulated employing the Treeage function. In one case, data was reported as fraction of patients consuming a specific resource, which was used for sampling within the model. Otherwise the reported point estimate, usually the average cost across the patient group, was used. If no Swedish data on a cost item was found, the cost was taken from studies reporting data from settings assumed similar to the Swedish.

All costs are reported in SEK year 2014 (USD 1=SEK 6.86; Euro 1=SEK 9.10), adjusted when necessary with the Swedish CPI. To adjust reported Gamma distributed parameters to the price level, only the second parameter, i.e. the scale parameter, was adjusted.

Medical treatment costs

Recent Swedish estimates on medical treatment costs were possible to obtain for all model diseases, see table 13. The costs are paid by the regional healthcare authorities.

Institutional care and technical aids costs

These costs include rehabilitation, terminal care, old age homes, support for individuals living at home, transportation and technical aids. In Sweden, institutional care and technical aids used by patients in their homes are the responsibility of the local authorities (municipalites, in Swedish: kommuner). The costs are not fully represented for any disease, see table 14. Estimates are not available for lung cancer and the only available costs for IHD are outdated, so the institutional care costs are probably underestimated.

Table 14. Costs for institutional care and technical aids. SEK 2014.

	mean	95% confidence interval	distribution	source	comment
Lung cancer	0	-	-		
COPD	0	-	-		Oxygen theraphy included in medical treatment costs
AMI year 1	16 680	-	Gamma 11;1502	Henriksson et al, 2014	Home care and nursing home
AMI year 2+	8 340	-	Gamma 11;751	Henriksson et al, 2014	Home care and nursing home
CHF	2 200	-	-	Agvall et al, 2005	Nursing home
IHD, age <65	3 140	-	-	Andersson & Kartman, 1995	Social services and aids, angina pectoris
IHD, age >64	8 260	-	-	Andersson & Kartman, 1995	Social services and aids, angina pectoris
Stroke year 1	82 130	-	Gamma 11;7184	Henriksson et al, 2014	Home care and nursing home
Stroke year 2+	41 070	-	Gamma 11;3593	Henriksson et al, 2014	Home care and nursing home

For peer review only - http://bmjopen.bmj.com/site/about/guidelines.xhtml

Table 15. Pharmaceutical costs. SEK 2014.

	mean	95% confidence interval	distribution	source	comment
Lung cancer	0	-	-		
COPD	0	-	-		included in medical treatment costs
AMI year 1	11 960	-	-	Mourad et al, 2013	
AMI year 2+	9 250	-	-	Mourad et al, 2013	
CHF	8 420	-	-	Agvall et al, 2005	
IHD	12 690	-	-	Mourad et al, 2013	Angina pectoris
Stroke year 1	2 120	-	-	Ghatnekar et al, 2013	
Stroke year 2+	2 820	-	-	Ghatnekar et al, 2013	

Pharmaceutical costs

Costs for pharmaceuticals in Sweden ought to be divided between the county councils and the patients, as patients pay a considerable share in co-payment. This is however not possible, given the data available. Table 15 therefore presents the drug costs to the regional healthcare authorities. The costs of pharmaceuticals dispensed during hospital stays are included in the medical treatment costs.

Informal care and other patient and relatives' costs

These costs include the value of care given to patients by relatives and other costs for patients or relatives, such as time, co-payments paid for health care and drugs as well as costs for transportation, modifications at home etc. Complete estimates could not be obtained for any disease, see table 16, except IHD which however might be outdated. Informal care in present-day Sweden probably constitute a sizeable part of total societal costs.

Table 16. Informal care and other patient and relatives' costs. SEK 2014.

	Mean	95% confidence interval	distribution	source	comment
Lung cancer	140 810	-	-	Gridelli et al, 2007	Informal care, estimated from 3 months home care
COPD	0	-	-		
AMI year 1	2 090	-	Gamma 44;48	Henriksson et al, 2014	Informal care
AMI year 2+	1 050	-	Gamma 44;24	Henriksson et al, 2014	Informal care
CHF	0	-	-		
IHD, age <65	5 180	-	-	Andersson & Kartman, 1995	Travel and time costs for healthcare contacts, angina pectoris
IHD, age 65+	2 500	-	-	Andersson & Kartman, 1995	Travel and time costs for healthcare contacts, angina pectoris
IHD	680	-	-	Andersson & Kartman, 1995	Informal care, angina pectoris
Stroke year 1	28 260	-	Gamma 44;636	Henriksson et al, 2014	Informal care
Stroke year 2+	14 130	-	Gamma 44;308	Henriksson et al, 2014	Informal care

For peer review only - http://bmjopen.bmj.com/site/about/guidelines.xhtml

Table 17. Productivity costs, morbidity. SEK 2014.

	mean	95% confidence interval	sd	distribution	source	comment
Lung cancer	0	-	-	-	Ford et al, 1999	Simulated in model:<br>9% of pat. 100% disability<br>20% of pat. 80% disability<br>40% of pat. 50% disability<br>31% of pat. 20% disability
					Statistics Sweden	Age- and gender-specific mean wages year 2014
COPD	21 800	6 011 - 42 583	-	-	Jansson et al, 2013	Moderate COPD
AMI year 1	38 180	-	-	Gamma 9;4242	Henriksson et al, 2014	
AMI year 2+	19 090	-	-	Gamma 9;2121	Henriksson et al, 2014	
CHF	29 880	-	49 210	-	Zethraeus et al, 1999	Difference year before and after disease onset
IHD	121 020	-	99 880	-	Mourad et al, 2013	Angina pectoris
Stroke year 1	194 100	-	-	Gamma 9;21567	Henriksson et al, 2014	
Stroke year 2+	97 050	-	-	Gamma 9;10783	Henriksson et al, 2014	

Productivity costs

The productivity costs only value the lost production because of morbidity before the age of 66 years, not mortality. The productivity costs for lung cancer is simulated within the model, via sampling from the fraction of patients on sick leave and combined with age- and gender-specific average monthly wages, including 40% employer taxes. Remaining data is taken from the literature, see table 17, and most estimates are recent. The costs are valued by the human capital method, and thus only include losses in salaried work before the official age of retirement.

The health effects

Life years lost

The number of life years lost (YLS) are calculated until the age of 95 years, and only for individuals dead in the modelled diseases. Life years lost are presented both discounted 3% and undiscounted.

QALYs

The number of quality-adjusted life years (QALYs) are calculated during healthy years and years spent diseased, until death or the age of 95 years.

The QoL weights used during healthy years are mean age group- and gender-specific population weights, see table 18. The data is somewhat dated, but it is the only general population QoL weights available in Sweden. The QoL of the age group 20-29 years is used

For peer review only - http://bmjopen.bmj.com/site/about/guidelines.xhtml

Table 18. Average Swedish population QoL weights.

Age group	men	women
20-29	0.91	0.88
30-39	0.90	0.86
40-49	0.86	0.85
50-59	0.84	0.82
60-69	0.83	0.78
70-79	0.81	0.78
80-88	0.74	0.74

Source: Burström et al, 2001

also for younger ages, and the QoL of the age group 80-88 years is used for those aged 89-95 years. This last assumption is probably an overestimate.

The disease-specific QoL used in the health states are all, except one, modelled as decrements from the average population age-group and gender-specific QoL, see table 19. For lung cancer no data was available on the marginal effect of the disease on the population average QoL, so a fixed value over the ages and genders had to be used.

Sensitivity analyses

Several univariate and multivariate sensitivity analyses have been performed. Analyses on some methodological issues, as well as a probabilistic sensitivity analysis, have also been performed. The analyses are reported for men and women aged 50 years.

To give another measure of the uncertainty surrounding the cost-effectiveness ratio, the 95% confidence interval for the difference between smokers and quitters is reported, calculated from the standard deviation of outcomes.

Table 19. QoL weights and QoL decrements due to disease.

	QoL	source
Health state weight		
Lung cancer	0.653	Nafees et al, 2008
Decrement from average QoL		
COPD	0.0142	Sullivan et al, 2005
AMI	0.0627	Henriksson et al, 2014
CHF	0.0700	Granström et al, 2012
IHD	0.0900	Granström et al, 2012
Stroke	0.1384	Henriksson et al, 2014

For peer review only - http://bmjopen.bmj.com/site/about/guidelines.xhtml

Univariate analyses

Univariate analyses have been performed on all model parameters:

A. disease risks: +100%, -50%

B. death risks: +-10%. (As the unrelated death risks for those aged over 84 years are so high they had to be adjusted by deducting 0.05 for the diseases stroke, IHD and AMI, and omitted for lung cancer, to enable the simulation.)

C. risk fractions of disease after quitting: +-0.1

D. all disease costs: +-25%

E. QoL weights: QoL weight 1 during healthy years

Multivariate analyses

Two sets of multivariate analyses have been performed:

F. high risk – low risk: death risks +100%, disease risks +10% and risk fractions +0.1 *vs* death risks -50%, disease risks –10% and risk fraction –0.1

G. high risk, high costs – low risk, low costs: death risks +100%, disease risks +10%, risk fraction +0.1 and all costs +25% *vs* death risks -50%, disease risks –10%, risk fractions –0.1 and all costs –25%

Analyses on methodological issues

Three analyses have been performed on methodological issues:

H. discount rate: 5%, 0%

I. perspective: healthcare and personal social services perspective (UK NICE perspective); excludes informal care and other patient and relatives' costs and productivity costs

J. recent Swedish data: only includes data from a Swedish context from year 2005 onwards. Excludes the data from Andersson & Kartman (1995) on institutional care and patient and relatives' costs for IHD, from Gridelli et al (2007) on lung cancer patient and relatives' care, from Ford et al (1999) for lung cancer productivity costs and from Zethraeus et al (1999) on CHF productivity costs

Probabilistic analysis

A bootstrap sampling was performed using the smoker and quitter Monte Carlo simulations of 10 000 runs. A sample of 1 000 from each simulation was drawn, with replacement, performed in Microsoft Excel. The mean of the difference in costs and QALYs between smokers and quitters was then calculated. This was replicated 1 000 times. The bootstrap is represented as a scatterplot in the cost-effectiveness plane.

For peer review only

Results

In this chapter, the model estimates of QALYs, YLS and societal costs are presented for men and women in some selected ages, mainly for validation purposes. More detailed simulation outcomes as well as the results of the sensitivity analyses are presented for men and women at age 50 years. Model estimates can be obtained for men and women for all ages between 15 and 95 years.

The model estimates

In table 20 the simulation results for QALYs (quality-adjusted life-years) experienced until the age of 95 years are presented, for the selected ages 15, 30, 50 and 70 years at the start of the simulations. As can be expected, the number of QALYs are highest in the younger age groups, and somewhat higher for women in most age groups. In the selected age groups, the differences between smokers and quitters are at a maximum at age 30; 0.68 for females and 0.81 for males. The confidence intervals, calculated via the mean and standard deviation (sd) from the 10 000 model runs, indicate that there are differences in QALYs between smokers and quitters.

The YLS (life-years saved) lost before the age of 95 years are presented in tables 21 and 22, discounted 3% and undiscounted. The differences in discounted YLS between smokers and quitters are somewhat higher than the differences in QALYs. The undiscounted YLS in table 22 show the number of years that smokers and quitters are expected to lose before the age of 95 years. For the ages 15, 30, and 50 the number of lost life-years is estimated at around 6 years for women smokers and 9 years for men, implying that the female smokers are estimated to live until age 89 and the male until age 86. In the oldest age group presented here, age 70, the number of lost life-years are only 1-2 years. The quitters are estimated to lose considerably fewer life-years; 1-4 years for the women and 3-5 years for

Table 20. QALYs, until age 95 years, discounted 3%.

age	smoker		quitter		difference			
	mean	sd	mean	sd	mean	95% CI		
women								
15	23.20	2.26	23.70	2.28	0.50	0.44	-	0.57
30	20.02	2.85	20.71	2.82	0.68	0.60	-	0.76
50	14.15	4.19	14.76	4.15	0.61	0.49	-	0.73
70	8.24	3.75	8.50	3.82	0.26	0.16	-	0.37
men								
15	23.21	2.84	23.83	2.70	0.63	0.55	-	0.70
30	19.65	3.20	20.46	3.19	0.81	0.72	-	0.90
50	13.18	4.34	13.95	4.47	0.77	0.65	-	0.89
70	6.78	3.61	7.15	3.76	0.37	0.27	-	0.48

For peer review only - http://bmjopen.bmj.com/site/about/guidelines.xhtml

Table 21. Life years lost (YLS), before age 95 years. Discounted 3%.

age	smoker		quitter		difference			
	mean	sd	mean	sd	mean	95% CI		
women								
15	0.97	1.90	0.23	0.87	0.74	0.70	-	0.78
30	1.55	3.02	0.51	1.83	1.04	0.97	-	1.11
50	2.35	4.82	1.49	4.09	0.86	0.74	-	0.99
70	1.22	3.31	0.92	2.98	0.30	0.22	-	0.39
men								
15	1.42	2.25	0.43	1.21	0.99	0.94	-	1.04
30	2.18	3.44	0.79	2.15	1.40	1.32	-	1.48
50	3.51	5.57	2.09	4.69	1.41	1.27	-	1.56
70	2.22	4.30	1.68	3.94	0.53	0.42	-	0.65

the men. As expected, the difference between smokers and quitters diminish with age, with a maximum at around 5 years for the females and around 6 years for the males at age 15.

The societal costs estimated for the smokers and quitters for the selected age groups are presented in table 23. The highest costs are found for age 50; 200 000 SEK and 250 000 SEK for the smokers and 130 000 and 170 000 for the quitters, in both cases higher among the men. The highest difference between smokers and quitters is however found at age 30, with a difference of 100 000 among the females and 120 000 among the males. The difference among the eldest, at age 70, is around 20 000 SEK. These cost differences reflect the amount that tobacco cessation interventions could spend on achieving one quitter and still be cost-saving.

Table 22. Life years lost (YLS), before age 95 years. Undiscounted.

age	smoker		quitter		difference			
	mean	sd	mean	sd	mean	95% CI		
women								
15	6.46	11.80	1.68	5.86	4.78	4.52	-	5.04
30	6.58	11.93	2.22	7.25	4.37	4.09	-	4.64
50	5.67	10.94	3.55	9.19	2.12	1.84	-	2.40
70	1.97	5.18	1.47	4.64	0.50	0.37	-	0.64
men							-	
15	9.25	13.51	3.05	7.89	6.20	5.89	-	6.50
30	9.21	13.39	3.51	8.68	5.70	5.39	-	6.02
50	8.42	12.57	5.01	10.53	3.40	3.08	-	3.73
70	3.56	6.70	2.68	6.11	0.87	0.70	-	1.05

For peer review only - http://bmjopen.bmj.com/site/about/guidelines.xhtml

Table 23. Societal costs. In SEK 2014 and discounted 3%.

age	smoker		quitter		difference			
	mean	sd	mean	sd	mean	95% CI		
women								
15	113 097	278 446	40 761	207 879	72 337	65 526	-	79 147
30	170 047	386 905	71 569	293 477	98 478	88 960	-	107 996
50	201 760	415 452	133 902	366 313	67 858	57 002	-	78 714
70	85 818	189 827	63 824	171 358	21 994	16 981	-	27 006
men								
15	145 233	320 143	54 148	227 222	91 085	83 390	-	98 779
30	216 626	453 147	92 782	349 085	123 844	112 632	-	135 055
50	254 279	484 787	168 598	434 603	85 681	72 920	-	98 442
70	101 358	188 991	80 927	184 794	20 431	15 250	-	25 611

Selected model outcomes

The underlying estimated disease outcome is presented in figures 2 and 3, for the age 50 years. For both women and men, there is a marked decrease for quitters in the number of diseased and dead in the model diseases, which is somewhat offset by an increase in the number of deaths in unrelated diseases. The number of diseased and deaths are higher for

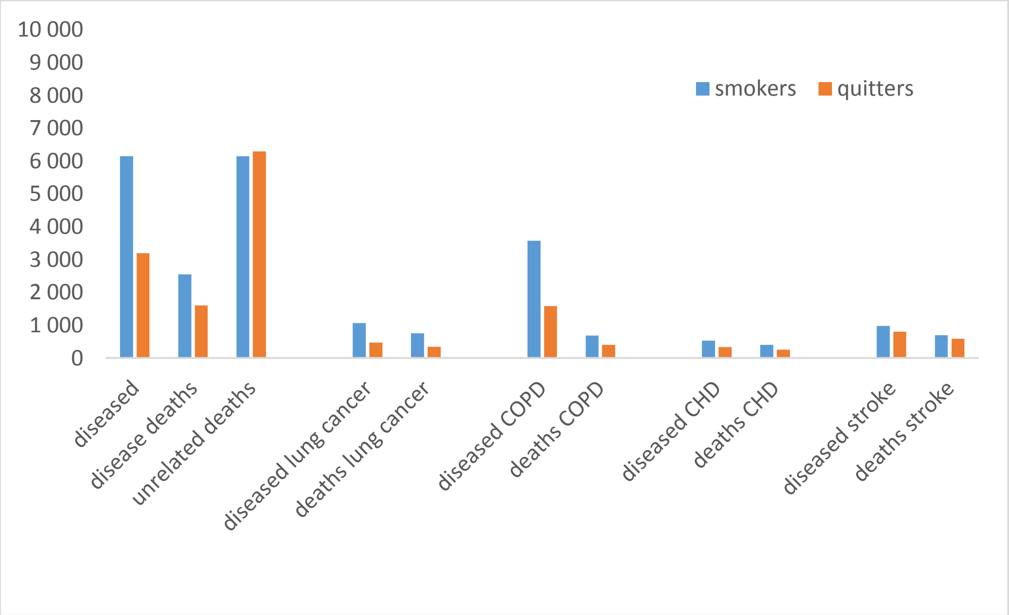

Figure 2. The disease outcome, number of diseased and dead per 10 000 for smokers and quitters, women aged 50 years.

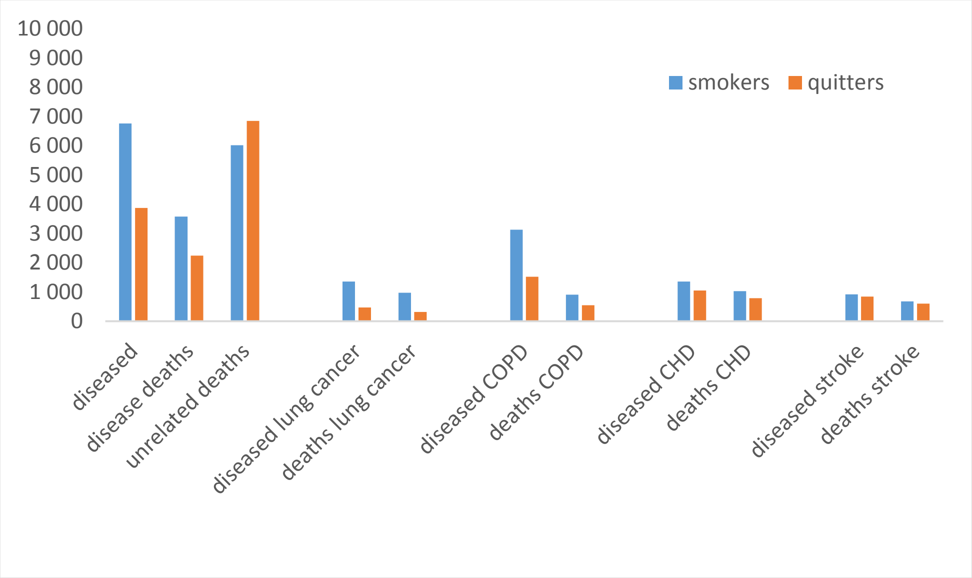

Figure 3. The disease outcome, number of diseased and dead per 10 000 for smokers and quitters, men aged 50 years.

men, mainly originating from CHD. The model disease with the highest smoking-related incidence is COPD, for both genders. The increase in unrelated deaths for the quitters is an example of competing risks, which decreases the difference in life-years and QALYs between smokers and quitters.

Table 24 and 25 shows the full model simulation results of the societal cost savings because of tobacco quitting at age 50 years. For women, the highest estimated savings are found in lung cancer, COPD and stroke at around 15-20 000 SEK per quitter. For men the cost savings because of lung cancer are considerable higher, at around 35 000, due to the higher incidence among the men. The cost item with the largest cost savings are medical treatment costs for both genders, at around 30 000 SEK. Most of the difference in savings between men and women originate from the productivity costs, possibly reflecting disease onset at younger ages among men. Note that a cost saving of zero means that no cost is being modelled, as cost data was lacking.

Table 24. Societal cost savings, in SEK 2014. Women aged 50 years.

	Lung cancer	COPD	AMI	CHF	IHD	Stroke	Sum
Medical treatment	5 171	13 573	2 337	439	3 410	5 500	30 430
Institutional care and technical aids	0	0	365	29	408	4 880	5 681
Pharmaceuticals	0	0	361	109	838	306	1 615
Informal care and other patient and relatives' costs	9 569	0	44	12	282	1 673	11 580
Productivity costs	3 971	6 456	192	243	3 228	4 462	18 552
Sum	18 711	20 029	3 300	832	8 166	16 821	67 858

For peer review only - http://bmjopen.bmj.com/site/about/guidelines.xhtml

Table 25. Societal cost savings, in SEK 2014. Men aged 50 years.

	Lung cancer	COPD	AMI	CHF	IHD	Stroke	Sum
Medical treatment	8 477	11 478	3 203	596	4 738	3 907	32 399
Institutional care and technical aids	0	0	456	39	596	3 379	4 470
Pharmaceuticals	0	0	473	148	1 165	214	2 000
Informal care and other patient and relatives' costs	15 685	0	59	16	377	1 164	17 301
Productivity costs	13 002	8 357	319	400	3 785	3 649	29 511
Sum	37 164	19 835	4 510	1 199	10 661	12 312	85 681

For peer review only

Sensitivity analyses

The results of the sensitivity analyses are presented on women and men at starting age 50 years. Figure 4 shows the results for women and figure 5 for men.

All analyses show a similar pattern between men and women, and also similar ranges. The univariate sensitivity analyses on the model parameters, analyses A to E, result in small changes in costs and QALYs. Also the multivariate analyses F and G, which are constructed as scenarios that allow the risk parameters to vary consistently upwards or downwards, and along with the costs in analysis G, show moderate changes from the base case estimates. The methodological choices have a more pronounced effect, as the largest difference in QALYs is achieved by varying the discount rate (analysis H) between 0 and 5%, which also affects the costs substantially. The two analyses that reflect the choices of which costs to include in the estimates, analysis I that reflects the UK NICE health care and social services perspective and analysis J that only include Swedish data published since the year 2005, both decrease the cost differences between smokers and quitters.

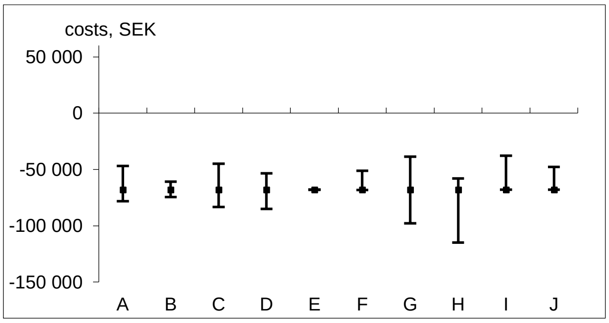

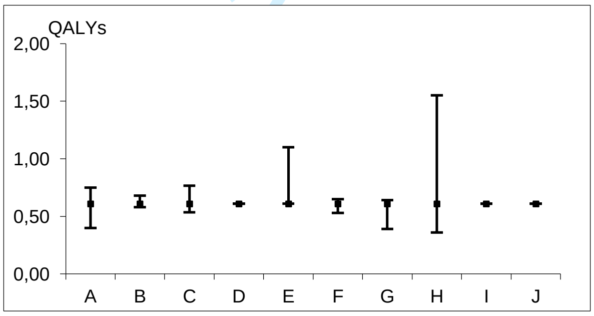

Figure 4. Sensitivity analyses on societal cost and QALY differences between smokers and quitters, women aged 50 years.

Notes: **A**. disease risks. **B**. death risks. **C**. risk fractions of disease after quitting. **D.** all costs. **E**. QoL weights. **F**. high risk – low risk. **G**. high risk, high costs – low risk, low costs. **H**. discount rate. **I**. perspective. **J.** recent Swedish data.

For peer review only - http://bmjopen.bmj.com/site/about/guidelines.xhtml

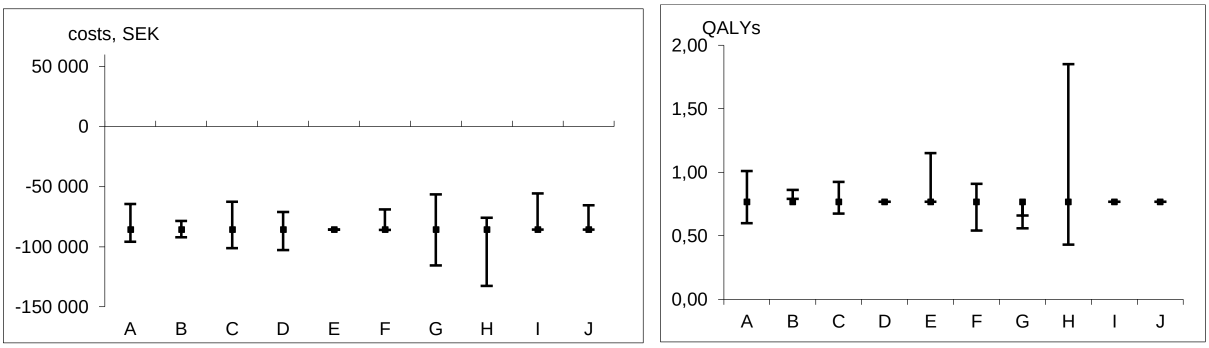

Figure 5. Sensitivity analyses on societal cost and QALY differences between smokers and quitters, men aged 50 years.

Notes: **A**. disease risks. **B**. death risks. **C**. risk fractions of disease after quitting. **D.** all costs. **E**. QoL weights. **F**. high risk – low risk. **G**. high risk, high costs – low risk, low costs. **H**. discount rate. **I**. perspective. **J.** recent Swedish data.

The scatter plot of the bootstrap analysis based on the microsimulation results for women and men aged 50 are shown in figures 6 and 7. The uncertainty is higher for the men, as the plots are more scattered. All plots are however situated in the cost decrease and QALY increase quadrant, with costs below -20 000 SEK and QALYs over 0.2.

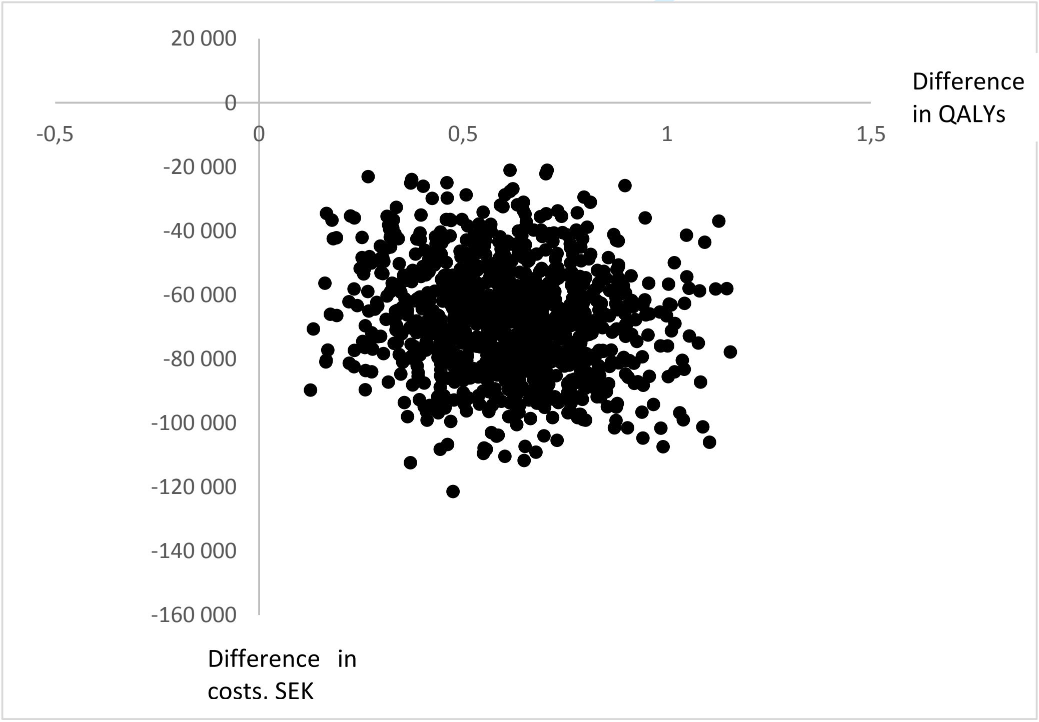

Figure 6. The cost-effectiveness plane with resultat från bootstrap, women aged 50 years.

For peer review only - http://bmjopen.bmj.com/site/about/guidelines.xhtml

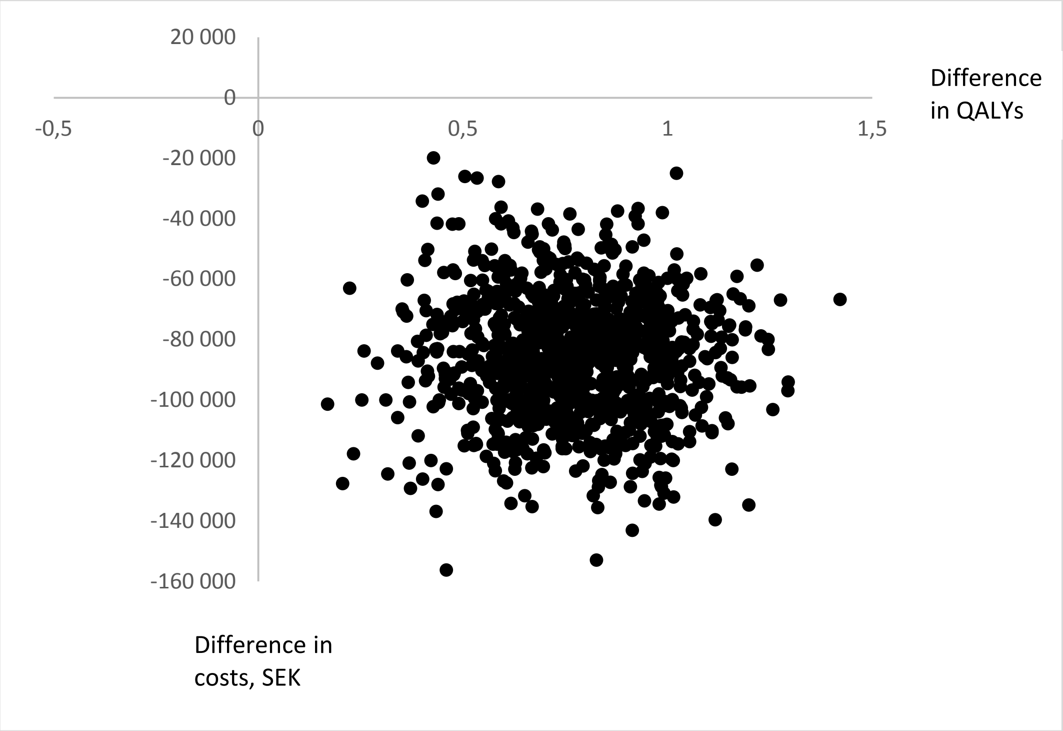

Figure 7. The cost-effectiveness plane with resultat from bootstrap, men aged 50 years.

For peer review only - http://bmjopen.bmj.com/site/about/guidelines.xhtml

Discussion: Model validity

The discussion of the model validity is structured around four aspects as proposed by McCabe & Dixon (2000): the structure of the model, the inputs to the model, the results of the model and the value of the model to the decision-maker.

The structure of the model

The structure of the model is a Markov model constructed for microsimulations, on the three most smoking-related disease groups; lung cancer, COPD, and CVD including stroke and CHD. The present updated version of the model includes one less CHD disease compared to the first version of the model, as unrecognized acute myocardial infarction now is included in the IHD disease, mainly because the disease definition is rarely used nowadays. Choosing only three disease groups is a clear simplification as smoking is known to cause hundreds of different diseases. The effects from smoking, and thus quitting, are furthermore confined to the individuals themselves; no side-effects on other individuals such as environmental tobacco smoke or smoking uptake are included. These two features leads to an underestimate of the true effects of tobacco quitting.

The same disease-specific approach has been taken by most other tobacco cessation models (Bolin, 2012), even though some of them include more diseases, such as asthma. Another approach would be to use the overall differences in mortality between current, former, and never-smokers taken from large US studies, as some early tobacco cessation models did (Secker-Walker et al, 1997; Tengs et al, 2001). In order not to overestimate the effects of quitting tobacco, we chose to model the smoking-related risk for certain diseases instead, as it is improbable that all differences in mortality and morbidity between smokers and former smokers are due to the smoking habit (Doll et al, 1994).

The model aims to reflect disease onset related to smoking tobacco. As disease in all the three disease groups included in model may be caused by other factors than smoking only the excess risks for smokers are modelled. For the diseases lung cancer and COPD this implies that the risk for smokers found in epidemiological studies is adjusted by the risk found for non-smokers. For the disease group CHD and stroke, where a large fraction of disease onset is caused by other factors than smoking, this adjustment for smokers´ excess risk was performed by setting the other risk factors in the risk function at minimal risk levels. This is an underestimate, as the risk factor levels among smokers can be expected to be at least as elevated as among the general population. The underestimate is aggravated by the fact that the functional form of the risk function results in a multiplier effect of the risk factors.

The present version of the model includes seven health states: lung cancer, COPD, stroke, and CHD divided into four diseases. This is a clear simplification, as the costs and QoL can be expected to vary considerable between patients with different severity levels within the diseases. This is particularly true for COPD which is a chronic progressive disease, i.e. the

For peer review only - http://bmjopen.bmj.com/site/about/guidelines.xhtml

diseased get more severely ill over time. However, a model with 7 health states with accompanying disease-specific death risks, costs and QoL weights is fairly complex as well as data-demanding. For the purposes of this study´s model, the division of diseases into severity levels was not deemed necessary.

An obvious problem with the model, inherent in all Markov models, are the mutually exclusive health states; any individual can only contract one disease, and once diseased the individual never recovers (apart from the very rare 5 year survivors in lung cancer). This feature implies both an overestimate and an underestimate of the true effects. The underestimate stems from the fact that co-morbidity is very common, especially among the individuals with the chronic diseases COPD, CHD, and stroke. The overestimate of costs and effects arise as individuals stay in the health states until death. If the costs and outcomes associated with the health states are taken from severely ill individuals, then these become grossly overestimated. This overestimate is partly offset by the use of separate costs for the first and subsequent years, for all societal costs due to AMI and stroke. In order not to overestimate the numbers of years spent in disease states, the possibility of dying in unrelated diseases is present in all health states. This feature is also included in the CHD Policy Model (Weinstein et al, 1987).

Most tobacco cessation models are built for cohort estimation (Bolin, 2012), but this model is constructed for individual-level estimation using the microsimulation methodology. As the data available admitted a microsimulation structure, e.g. the risk functions, the methodology was chosen as the advantages to model and to obtain a richer data set with results that reflect the heterogeneity of outcomes between individuals was deemed to offset the disadvantages of calculation burden. The use of the software Treeage also facilitates the use of microsimulation. Age- and gender-specific estimates can thus be obtained from the model, between ages 15 and 95 years.

The model stages are one-year long, which seems accurate given the risk estimates and the long time horizon of the model. The reason for the model maximum age of 95 years is the lack of risk estimates for older ages. Some extrapolations of risk estimates to the age of 95 years indeed resulted problematic, as some disease-specific death risks expressed as multipliers of the average age-specific death risk resulted in risks above 1. Further extrapolations beyond the age of 95 years were deemed unnecessary, as most of the relevant differences between smokers and quitters would have arisen by that age.

The inputs of the model

The second aspect of model validity is the inputs of the model. The model contains a large number of data taken from different sources. This is of course a threat to the internal validity of the model, shared with most models. However, the data have been chosen to reflect current Swedish circumstances. The current updated version of the model has exchanged almost all cost data, if more recent estimates were available, and all death risks to recent Swedish register data. As the number of studies on any particular data items are few, no meta-analysis or any other synthesis of data was carried out.

For peer review only - http://bmjopen.bmj.com/site/about/guidelines.xhtml

The disease risks are of course are pivotal for the result. The lung cancer disease risks are probably the best that can be obtained, from a large epidemiological study (Peto et al, 2000). The risk equation used for CHD and stroke is taken from the Framingham studies, and even though there are more recent risk scores developed from the study (D'Agostini et al, 2008), the Anderson et al (1991) risk functions are still frequently employed. The disease COPD has been the subject of a large long term epidemiological study in Sweden, The Obstructive Lung Disease in Northern Sweden (OLIN) (Lundbäck et al, 1991), which is thus the most relevant data source for the model.

In the model, there is an increased risk for a smoking-related disease remaining for some years after the tobacco cessation, in accordance with epidemiological evidence (Surgeon General, 1990; Omenn et al, 1990). The feature is also considered a marker of high quality tobacco cessation models (Bolin et al, 2012).

The majority of the cost data are taken from Swedish studies published during the 2010s. To take fully advantage of the microsimulation structure and to obtain stochastic estimates, the preferred data sources were the ones reported as distributions, i.e. as Gamma parameters or bootstrapped 95 percent confidence intervals. If no Swedish data was found, an international estimate was instead used in order to seek to represent the full societal costs. However, apart from certain cost items and for some of the diseases, the lack of data results in considerable underestimates of the true societal costs. This is particularly true in the cases of the costs for care, both institutional and informal. The institutional care could amount to considerable costs, exemplified by the costs for stroke and AMI patients, see table 14. In particular for lung cancer the lack of data results in considerable underestimates of the true disease-related costs. This is why the possible overestimate of the informal care for the disease, obtained from an Italian study, probably does not bias the overall result. To investigate the issue, one sensitivity analysis only included recent Swedish data. The analysis lead to decreases in cost savings for quitters aged 50 years of around 30%.

The QoL estimates are constructed as disease-specific decrements from the average age- and gender-specific QoL, except for lung cancer for which no QoL decrement could be found (De Geer et al, 2013). The average population age- and gender-specific QoL weights, which are certainly not 1, are also used during healthy years for the base case estimates. This means that the model assumes that an individual that avoids the smoking-related diseases is not having perfect health, but the health of an average Swede at the same age, as recommended (Gold et al, 1996).

The stated purpose of the model is to reflect the societal perspective, which for Sweden includes the morbidity productivity costs, but not the productivity costs resulting from mortality. All the model data on productivity costs value them according to the human capital approach for individuals under the age of 65, the customary Swedish age of retirement.

A full societal perspective might also include other aspects, considering that this is a model on individuals that are participating in an intervention that aims to change their lifestyle. The previous version of the tobacco cessation model, version 1 (Johansson, 2004), reported

For peer review only - http://bmjopen.bmj.com/site/about/guidelines.xhtml

sensitivity analyses that modelled some effects on the tobacco quitters, by including savings from cigarette purchases and a decreased QoL because of withdrawal effects during the first year. When that analysis was applied to an intervention, a decreased QoL during the first year was also deducted for the smokers that failed to quit, as the failure to achieve a personal goal might to lead to a decrease in QoL.

The results of the model

The third aspect of model validity is the results of the model, e.g. a comparison with reality or with other study results. A direct comparison with reality is not possible, since the model covers the ages 15-95 years, with a follow-up time of 80 years for the youngest age group.

The model estimates that around 60% of the women and 70% of the men aged 50 at the start of the simulations will contract one of the modelled diseases, and that around 50% of those will die in the diseases before the age of 95 years. The disease risks for the quitters at age 50 are not eliminated; 30-40% of them will still contract the smoking-related diseases because of remaining disease risks after quitting. As expected, the unrelated deaths increase among the quitters, in sum leading to an increase in YLS (undiscounted) of 2-3 years for those quitting at age 50, compared with continuing smokers. The increases in QALYs (discounted 3%) are smaller because of less-than-perfect health among those aged 50 years and above; 0.61 for women and 0.77 for men. The disease outcomes are fairly similar to the estimates from the previous versions of the model, but because of decreased death risks, the outcomes in terms of YLS and QALYs are considerably higher. The 2004 version of the model estimated an increased YLS of 0.93 and of 1.66 for women and men aged 50-54 years, and QALY gains of 0.36 and 0.71, respectively. The differences are due to the longer time perspective of the present version, 95 years versus 85 years, and the somewhat decreased case-fatality risk (i.e. the mortality risk among those with disease) because of improvements in medical technologies during the past decade.

Apart from increases in health, the societal cost savings because of quitting smoking are considerable. For men, the cost savings amount to around 100 000 SEK for quitters aged between 15 and 50 years, and around 70-90 000 SEK for women. Even in the age group 70 years there are estimated cost savings of around 20 000 SEK per individual quitter. This implies that substantial funds could be invested in smoking cessation interventions, and the interventions would still be cost-effective, or even cost-saving. The cost savings in the present model are considerably higher than those of the previous model, in part due to changes in price year.

Comparisons of model estimates with other models' are difficult to perform, as the time horizon, costs included, jurisdiction, and the diseases included differ. Among the recently reported model estimates (Bolin, 2012), there are two Australian models. The model developed within ACE (Bertram et al, 2007) report estimates of life-years saved that are considerable higher than the present model's; 5.7 years for men and 6.6 years for women in age group 50-54 years. That model time horizon is however 100 year, but it is unlikely

For peer review only - http://bmjopen.bmj.com/site/about/guidelines.xhtml

that the feature fully explains the difference between the model estimates. The estimates of average health care cost saved per quitter (inferred from table 3) however seems to be very similar to the present model's; around 33 000 SEK. The other Australian model, the Quit Benefits Model (Hurley & Mathews, 2007), reports considerably lower estimates of both life-years and health care costs saved, e.g. 0.1 – 0.2 YLS and QALYs saved for men and women quitters. The lower estimates, in comparison with both the present model and the ACE model, are probably partly explained by the time horizon of only ten years.

There have been two, to my knowledge, reports of tobacco cessation model estimates for Sweden, one using the Benesco model (Bolin et al, 2007) and one using an extended version of the HECOS model (Bolin et al, 2006). Comparison with those model estimates are unfortunately not possible, due to lack of reporting detail. However, estimates from the previous version of this model were fairly consistent with the HECOS model estimates (Orme et al, 2001) for Sweden, available at the time (Johansson, 2004).

The value of the model to the decision-maker

The fourth aspect of validity is the value of the model to the decision-maker. There are several models on tobacco cessation that conforms to international recommendations on how to perform cost-effectiveness analyses (Bolin, 2012). This model however reflect Swedish circumstances, with Swedish cost and QoL data, why the model might be useful for Swedish decision-makers.

We hope that the model will be used to perform economic evaluations of a range of tobacco cessation interventions. For tobacco prevention interventions, i.e. prevention of initiation of smoking, another model version, version 2, has been constructed and is available for analyses. The use of these models will in time enable incremental and marginal calculations of the cost-effectiveness of different tobacco interventions and their components and suitable target groups. The basis for decisions on which tobacco cessation and prevention interventions to implement will then be more comprehensive.

Another frequent use of models is to forecast future events. This model is not suitable for estimating what the costs of smoking will be in the future. The reason is that the model does not incorporate any adjustments of possible future developments. The risk of smoking is based on studies with follow-up periods of sometimes 30 years, which means that the risks are reflecting the smoking behaviour among smokers 30 years ago. The changes in cigarette content and in the frequency of smoking might lead to changes in disease risk in the future. Also the costs for the smoking-related diseases might change in the future, because of changes in health care technology. Another example would be the value of the morbidity productivity costs, as well as informal care, as wages and productivity often are expected to increase in the future.

Nevertheless, the model actually forecasts what the costs for smokers and quitters will be in 80 years' time, for the youngest age group. That implies that we know that the model forecasts will be wrong, but it is of minor significance as the model is constructed to be used for comparisons between two groups, smokers and quitters, thus eliminating some

For peer review only - http://bmjopen.bmj.com/site/about/guidelines.xhtml

of the biases. Furthermore, the model is constructed to be used now, for present-day decisions, which have to be based on present-day information.

The uncertainty

Another aspect of model validity is the uncertainty surrounding the model estimates.

The univariate sensitivity analyses on the model parameters (analyses A-F in figures 4 and 5 for men and women aged 50) show minor deviations from the base case result, while the multivariate analysis on costs and risks combined (analysis G) affects in particular the cost estimates. The methodological choices affect the results to a greater extent, with the discount rate (H) heavily influencing the QALYs and the more restricted perspective (I) decreasing the cost-savings. The multivariate analysis that only include higher-quality data (J) also imply decreases in the cost differences between smokers and quitters, but the difference remains substantial; around 50 000 SEK for females aged 50 years and 60 000 SEK for men, respectively. The overall conclusion from the parameter sensitivity analyses is that the QALY gains are at least 0.35 and 0.40 and the cost savings at least SEK 35 000, for female and male quitters aged 50, respectively.

The probabilistic analysis shows no uncertainty whether quitting tobacco leads to cost-savings and increases in QALYs, as all bootstraps are placed in the southeast quadrant of the cost-effectiveness plane. The bootstrap results exhibit a mixture of first and second order uncertainty, as it reflects both the probabilistic structure of the Markov model and the simulation of some parameter values (Briggs, 2000).

Another measure of uncertainty is the confidence intervals around the estimated mean differences, reported in tables 20-23. However, that measure is not fully appropriate as the large sample sizes of the Monte Carlo simulation (10 000 runs) diminishes the standard error of the mean (Briggs, 2000).

The structural uncertainty of the model, i.e. whether the results would be different if the model would have been constructed in another way, have not been studied. Alternatives to the chosen model structure could have been deterministic or discreet event simulations, more or less health states, other functional forms of risk functions, and other subgroups than men and women and five-year age-groups model results. The flaw is however shared with most tobacco quitting models (Bolin, 2012).

Checking for technical errors

The model contains a large number of trackers, i.e. variables that count events, to enable checking for technical errors. Tentative runs were executed after the introduction of every new variable, with cost items undiscounted, and the simulation results examined manually. Thus, the model has been thoroughly checked for technical errors.

For peer review only - http://bmjopen.bmj.com/site/about/guidelines.xhtml

Conclusions

The aim of this study is to develop a model predicting health and economic consequences of smoking cessation, to be used for cost-effectiveness analyses of smoking cessation interventions. The updated model strives to incorporate data that is recent, accurate and appropriate for Sweden in year 2015. The model also adhere to Swedish recommendations on how to perform cost-effectiveness analyses within the health care sector. Data is however lacking to completely fulfil these requirements. Many model parameters are based on very few studies. Some information just does not exist, at least not accessible to us.

These are issues shared with most model, however. The purpose of modelling is to assemble the most accurate information at a point of time, to enable decision-making at that particular point of time. This is in accordance with one of the fundamentals of economics: decision-making under uncertainty, which implies that decisions have to be made even if there is no full information. We hope that the model will be applied to a range of different tobacco cessation interventions, which in time will enable a more comprehensive basis for decision-making.

For peer review only

For peer review only - http://bmjopen.bmj.com/site/about/guidelines.xhtml

References

Agvall B, Borgquist L, Foldevi M, Dahlström M. Cost of heart failure in Swedish primary healthcare. Scandinavian Journal of Primary Health Care 2005;23:227-232.

Anderson KM, Odell PM, Wilson PWF, Kannel WB. Cardiovascular disease risk profiles. American Heart Journal 1991;121:293-298.

Andersson F, Kartman B. The cost of angina pectoris in Sweden. Pharmacoeconomics 1995;8:233-44.

Bertram MY, Lim SS, Wallace AL, Vos T. Costs and benefits of smoking cessation aids: making a case for public reimbursement of nicotine replacement therapy in Australia. Tobacco Control 2007; 16:255-260.

Bolin K. Economic evaluation of smoking-cessation therapies -A critical and systematic review of simulation models. Pharmacoeconomics 2012;30: 551-556.

Bolin K, Lindgren B, Willers S. The cost utility of bupropion in smoking cessation health programs. Simulation model results for Sweden. Chest 2006;129:651-660.

Bolin K, Wilson K, Benhaddi H, de Nigris E, Marbaix S, Mörk A-C, Aubin H-J. Cost-effectiveness of varenicline compared with nicotine patches for smoking cessation –results from four European countries. European Journal of Public Health 2007;19:650-654.

Briggs A, Sculpher M. An introduction to Markov modelling for economic evaluation. Pharmacoeconomics 1998;13:397-409.

Briggs AH. Handling uncertainty in cost-effectiveness models. Pharmacoeconomics 2000;17:479-500.

Burström K, Johannesson M, Diderichsen F. Swedish population health-related quality of life results using the EQ-5D. Quality of Life Research 2001;10:621-635.

Caro JJ, O´Brien JA, Hollenbeak CS, Spackman E, Ben-Joseph R, Okamoto LJ, Paramore LC. Economic burden and risk of cardiovascular disease and diabetes in patients with different cardiometabolic risk profiles. Value in Health 2007;10:S12-S20.

D'Agostino RB, Vasan RS, Pencina MJ, Wolf PA, Cobain M, Massaro JM, Kannel WB. General cardiovascular risk profile for use in primary care: The Framingham Heart Study. Circulation 2008;117:743-753.

De Geer A, Eriksson J, Finnern HW. A cross-country review of data collected on non-small cell lung cancer (NSCLC) patients in cancer registries, databases, retrospective and non-randomized prospective studies. Journal of Medical Economics 2013:16; 134–149.

Doll R, Peto R, Wheatley K, Gray R, Sutherland I. Mortality in relation to smoking: 40 years' observation on male British doctors. BMJ 1994;309:901-911.

Drummond MF, Sculpher MJ, Torrance GW, O´Brien B, Stoddart GL. Methods for the economic evaluation of health care programmes. (3rd edition). Oxford: Oxford University Press; 2005.

For peer review only - http://bmjopen.bmj.com/site/about/guidelines.xhtml

Ford ES, Kelly AE, Teutsch SM, Thacker SB, Garbe PL. Radon and lung cancer: A cost-effectiveness analysis. American Journal of Public Health 1999;89:351-357.

Ghatnekar O, Asplund, K, Glader E-K, Persson U. Costs for stroke in Sweden 2009 and developments since 1997. International Journal of Technology Assessments in Health Care 2014;30:203–209.

Gold MR, Siegel JE, Russell LB, Weinstein MC, eds. Cost-effectiveness in health and medicine. New York: Oxford University Press; 1996.

Granström O, Levin L-Å, Henriksson M. Cost-effectiveness of candesartan versus losartan in the primary preventive treatment of hypertension. ClinicoEconomics and Outcomes Research 2012;4:313–322.

Gridelli C, Ferrara C, Guerriero C, Palazzo S, Grasso G, Pavese I, Satta F, Bajetta E, Cortinovis D, Barbieri F, Gebbia V, Grossi F, Novello S, Baldini E, Gasparini G, Latino W, Durante E, Giustini L, Negrini C. Informal caregiving burden in advanced non-small cell lung cancer: The HABIT study. Journal of Thoracic Oncology 2007;2:475–480.

Henriksson M, Nikolic E, Ohna A, Wallentin L, Janzon M. Ticagrelor treatment in patients with acute coronary syndrome is cost-effective in Sweden and Denmark. Scandinavian Cardiovascular Journal 2014;48:138–147.

Holm LE, Gunnarskog J, Stenbeck M. In Stenbeck M, Rosén M (eds). Cancer survival in Sweden in 1961-1991. Acta Oncologica 1995; 34(suppl 4): 39-47.

Jansson S-A, Backman H, Stenling A, Lindberg A, Rönmark E, Lundbäck B. Health economic costs of COPD in Sweden by disease severity –has it changed during a ten years period? Respiratory Medicine 2013;107:1931-1938.

Johansson P. A model for economic evaluations of smoking cessation interventions – technical report. Version 1. Centre for Public Health, Stockholm county council; 2004. Available at: http://www.publichealtheteconomics.se/wp-content/uploads/2016/06/smoking-cessation-cea-model.pdf

Lindberg A, Eriksson B, Larsson L-G, Rönmark E, Sandström T, Lundbäck B. Seven-year cumulative incidence of COPD in an age-stratified general population sample. Chest 2006;129:879–885.

Lundbäck B, Nyström L, Rosenhall L, Stjernberg N. Obstructive lung disease in northern Sweden: respiratory symptoms assessed in a postal review. European Respiratory Journal 1991; 4:257–266.

Lundbäck B, Eriksson B, Lindberg A, Ekerljung L, Muellerova H, Larsson L-G, Rönmark E. A 20-year follow-up of a population study-based COPD cohort -Report from the Obstructive Lung Disease in Northern Sweden Studies. COPD 2009;6:4,263-271.

McCabe C, Dixon S. Testing the validity of cost-effectiveness models. Pharmacoeconomics 2000;17:501-513.

Mourad G, Alwin J, Strömberg A, Jaarsma T. Societal costs of non-cardiac chest pain compared with ischemic heart disease –a longitudinal study. BMC Health Services Research 2013;13:403.

For peer review only - http://bmjopen.bmj.com/site/about/guidelines.xhtml

Nafees B, Stafford M, Gavriel S, Bhalla S, Watkins J. Health state utilities for non small cell lung cancer. Health and Quality of Life Outcomes 2008;6:84.

Omenn GS, Anderson KW, Kronmal RA, Vlietstra RE. The temporal pattern of reduction of mortality risk after smoking cessation. American Journal of Preventive Medicine 1990;6:251-257.

Orme ME, Hogue SL, Kennedy LM, Paine AC, Godfrey C. Development of the health and economic consequences of smoking interactive model. Tobacco Control 2001;10:55-61.

Peto R, Darby S, Deo H, Silcocks P, Whitley E, Doll R. Smoking, smoking cessation, and lung cancer in the UK since 1950: combination of national statistics with two case-control studies. BMJ 2000;321:323-9. (Data supplement).

Secker-Walker RH, Worden JK, Holland RR, Flynn BS, Detsky AS. A mass media programme to prevent smoking among adolescents: costs and cost effectiveness. Tobacco Control 1997;6:207-212.

Sonnenberg FA, Beck JR. Markov models in medical decision making: A practical guide. Medical Decision Making 1993;13:322-38.

Statistics Sweden (SCB). Statistics database. www.statistikdatabasen.scb.se Accessed October 2015.

Sullivan PW, Lawrence WF, Ghushchyan V. A national catalogue of preference-based scores for chronic conditions in the United States. Medical Care 2005;43:736-749.

Surgeon General. The health benefits of smoking cessation. U.S. Department of Health and Human Services, Public Health Service, Centres for Disease Control, Center for Chronic Disease Prevention and Health Promotion, Office on Smoking and Health. DHHS Publication No. (CDC) 90-8416. 1990.

Swedish Association of Local Authorities and Regions (SALAR). KPP- database. Accessed 2015-10-01 at https://stat.skl.se/kpp/index.htm

Swedish National Board of Health and Welfare. Statistics database. Diagnoses in inpatient care from the Hospital Discharge Register. Accessed 2015-10-01 at www.socialstyrelsen.se/statistik/statistikdatabas/diagnoserislutenvard

Swedish National Board of Health and Welfare. The Swedish AMI Statistics. Accessed 2015-10-01 at www.socialstyrelsen.se/statistik/statistikdatabas/hjartinfarkter

Swedish National Board of Health and Welfare. The Swedish Stroke Statistics. Accessed 2015-10-01 at www.socialstyrelsen.se/statistik/statistikdatabas/stroke

Swedish National Board of Health and Welfare. The Swedish Causes of Death Register. Accessed 2015-10-01 at www.socialstyrelsen.se/statistik/statistikdatabas/dodsorsaker

Swedish National Heart Failure Register. Accessed 2015-10-01 at http://www.ucr.uu.se/rikssvikt/

Talbäck M, Rosén M, Stenbeck M, Dickman PW. Cancer patient survival in Sweden at the beginning of the third millennium -Predictions using period analysis. Cancer Causes and Control 2004;15:967-976. Accessed 2015-10-10 at www.socialstyrelsen.se/statistics/cancersurvival

For peer review only

Tengs TO, Osgood ND, Chen LL. The cost-effectiveness of intensive national school-based anti-tobacco education: Results from the Tobacco Policy Model. Preventive Medicine 2001;33:558-570.

TLV (Swedish Dental and Pharmaceutical Benefits Agency). General guidelines for economic evaluations from the Pharmaceutical Benefits Board (LFNAR 2003:2). Accessed 2016-04-24 at http://tlv.se/Upload/English/Guidelines-for-economic-evaluations-LFNAR-2003-2.pdf

Weinstein MC, Coxson PG, Williams LW, Pass TM, Stason WB, Goldman. L. Forecasting coronary heart disease incidence, mortality and cost: The Coronary Heart Disease Policy Model. American Journal of Public Health 1987;77:1417-1426.

Zethraeus N, Molin T, Henriksson P, Jönsson B. Costs of coronary heart disease and stroke: the case of Sweden. Journal of Internal Medicine 1999;246:151-159.

For peer review only

For peer review only - http://bmjopen.bmj.com/site/about/guidelines.xhtml

Consolidated Health Economic Evaluation Reporting Standards (CHEERS) statement

Section/item	Item No	Recommendation	Reported on page No/ line No
**Title and abstract**			
Title	1	Identify the study as an economic evaluation or use more specific terms such as "cost-effectiveness analysis", and describe the interventions compared.	Title, page 1
Abstract	2	Provide a structured summary of objectives, perspective, setting, methods (including study design and inputs), results (including base case and uncertainty analyses), and conclusions.	Abstract, page 3
**Introduction**			
Background and objectives	3	Provide an explicit statement of the broader context for the study. Present the study question and its relevance for health policy or practice decisions	Page 6, lines 1-17
**Methods**			
Target population and subgroups	4	Describe characteristics of the base case population and subgroups analysed, including why they were chosen.	Page 6-8
Setting and location	5	State relevant aspects of the system(s) in which the decision(s) need(s) to be made.	Page 3 lines 14-17<br>Page 9, lines 7-8
Study perspective	6	Describe the perspective of the study and relate this to the costs being evaluated.	Page 9, lines 17-24
Comparators	7	Describe the interventions or strategies being compared and state why they were chosen.	Page 7. lines 3-8,
Time horizon	8	State the time horizon(s) over which costs and consequences are being evaluated and say why appropriate.	Page 12, lines 2-13
Discount rate	9	Report the choice of discount rate(s) used for costs and outcomes and say why appropriate.	Page 9, line 24
Choice of health outcomes	10	Describe what outcomes were used as the measure(s) of benefit in the evaluation and their relevance for the type of analysis performed.	Page 10, lines 17-23
Measurement of effectiveness	11a	*Single study-based estimates:* Describe fully the design features of the single effectiveness study and why the single study was a sufficient source of clinical effectiveness data.	Page 8, table 1

For peer review only

Section/item	Item No	Recommendation	Reported on page No/ line No
	11b	*Synthesis-based estimates*: Describe fully the methods used for identification of included studies and synthesis of clinical effectiveness data.	Not applicable
Measurement and valuation of preference based outcomes	12	If applicable, describe the population and methods used to elicit preferences for outcomes.	Not applicable
Estimating resources and costs	13a	*Single study-based economic evaluation:* Describe approaches used to estimate resource use associated with the alternative interventions. Describe primary or secondary research methods for valuing each resource item in terms of its unit cost. Describe any adjustments made to approximate to opportunity costs.	Not applicable
	13b	*Model-based economic evaluation:* Describe approaches and data sources used to estimate resource use associated with model health states. Describe primary or secondary research methods for valuing each resource item in terms of its unit cost. Describe any adjustments made to approximate to opportunity costs.	Page 11, lines 21-25<br>Page 12, lines 1-14<br>Appendix 1
Currency, price date, and conversion	14	Report the dates of the estimated resource quantities and unit costs. Describe methods for adjusting estimated unit costs to the year of reported costs if necessary. Describe methods for converting costs into a common currency base and the exchange rate.	Page 9, lines 17-20
Choice of model	15	Describe and give reasons for the specific type of decision-analytical model used. Providing a figure to show model structure is strongly recommended.	Page 11, lines 21-25<br>Page 12, lines 1-6<br>Appendix 1
Assumptions	16	Describe all structural or other assumptions underpinning the decision-analytical model.	Appendix 1
Analytical methods	17	Describe all analytical methods supporting the evaluation. This could include methods for dealing with skewed, missing, or censored data; extrapolation methods; methods for pooling data; approaches to validate or make adjustments (such as half cycle corrections) to a model; and methods for handling population heterogeneity and uncertainty.	Page 12, lines 15-20<br>Appendix 1
**Results**			

For peer review only

Section/item	Item No	Recommendation	Reported on page No/ line No
Study parameters	18	Report the values, ranges, references, and, if used, probability distributions for all parameters. Report reasons or sources for distributions used to represent uncertainty where appropriate. Providing a table to show the input values is strongly recommended.	Appendix 1
Incremental costs and outcomes	19	For each intervention, report mean values for the main categories of estimated costs and outcomes of interest, as well as mean differences between the comparator groups. If applicable, report incremental cost-effectiveness ratios.	Page 15, Table 3<br>Page 17, Table 4
Characterising uncertainty	20a	*Single study-based economic evaluation:* Describe the effects of sampling uncertainty for the estimated incremental cost and incremental effectiveness parameters, together with the impact of methodological assumptions (such as discount rate, study perspective).	Not applicable
	20b	*Model-based economic evaluation:* Describe the effects on the results of uncertainty for all input parameters, and uncertainty related to the structure of the model and assumptions.	Page 17, lines 14-24<br>Page 18, lines 1-9<br>Page 18, figure 1
Characterising heterogeneity	21	If applicable, report differences in costs, outcomes, or cost-effectiveness that can be explained by variations between subgroups of patients with different baseline characteristics or other observed variability in effects that are not reducible by more information.	Not applicable
**Discussion**			
Study findings, limitations, generalisability, and current knowledge	22	Summarise key study findings and describe how they support the conclusions reached. Discuss limitations and the generalisability of the findings and how the findings fit with current knowledge.	Pages 18-20
**Other**			
Source of funding	23	Describe how the study was funded and the role of the funder in the identification, design, conduct, and reporting of the analysis. Describe other non-monetary sources of support.	Page 21 "Funding"
Conflicts of interest	24	Describe any potential for conflict of interest of study contributors in accordance with journal policy. In the absence of a journal policy, we recommend authors comply with International Committee of Medical Journal Editors recommendations.	Page 21 "Competing interests"

The CHEERS statement checklist format is based on the format of the CONSORT statement checklist

For peer review only

For peer review only
